# Accurate structure models and absolute configuration determination using dynamical effects in continuous-rotation 3D electron diffraction data

**DOI:** 10.1038/s41557-023-01186-1

**Published:** 2023-04-20

**Authors:** Paul B. Klar, Yaşar Krysiak, Hongyi Xu, Gwladys Steciuk, Jung Cho, Xiaodong Zou, Lukas Palatinus

**Affiliations:** 1 Institute of Physics, Czech Academy of Sciences, Prague, Czech Republic; 2 Department of Geosciences, University of Bremen, Bremen, Germany; 3 Institute of Inorganic Chemistry, Leibniz University Hannover, Hannover, Germany; 4 Department of Materials and Environmental Chemistry, Stockholm University, Stockholm, Sweden

**Keywords:** Materials chemistry, Techniques and instrumentation, Analytical chemistry

## Abstract

Continuous-rotation 3D electron diffraction methods are increasingly popular for the structure analysis of very small organic molecular crystals and crystalline inorganic materials. Dynamical diffraction effects cause non-linear deviations from kinematical intensities that present issues in structure analysis. Here, a method for structure analysis of continuous-rotation 3D electron diffraction data is presented that takes multiple scattering effects into account. Dynamical and kinematical refinements of 12 compounds—ranging from small organic compounds to metal–organic frameworks to inorganic materials—are compared, for which the new approach yields significantly improved models in terms of accuracy and reliability with up to fourfold reduction of the noise level in difference Fourier maps. The intrinsic sensitivity of dynamical diffraction to the absolute structure is also used to assign the handedness of 58 crystals of 9 different chiral compounds, showing that 3D electron diffraction is a reliable tool for the routine determination of absolute structures.

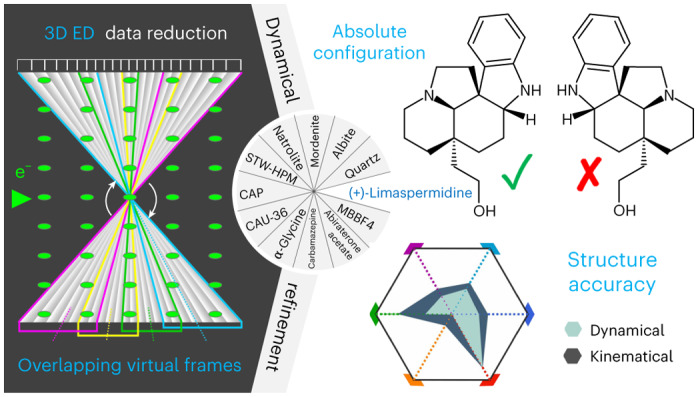

## Abstract (web summary)

Continuous-rotation 3D electron diffraction is a powerful technique to determine structures from nanocrystals. A data treatment method that takes into account dynamical diffraction effects has now been shown to lead to more accurate structure models, better sensitivity to weak signals and a reliable determination of absolute configuration—even for materials containing only light atoms.

## Main

Knowledge of the atomic structure of a material is the basis for understanding and optimizing its properties. This is underlined by the fact that the most cited paper of the twenty-first century describes a tool to solve and refine crystal structures from X-ray, neutron and electron diffraction (ED) experiments^1^. In the past decade, three-dimensional electron diffraction (3D ED) made huge technological and methodological steps forward^2–4^ and is a rapidly growing method to determine the crystal structure of submicrometric crystals by means of transmission electron microscopy (TEM). The 3D ED has proven its utility for all classes of crystalline materials, including metal–organic frameworks (MOFs), zeolites, minerals, pharmaceutical compounds and proteins^5^. Since 2019, continuous-rotation 3D ED is the predominant data acquisition mode (Supplementary Fig. 1), which allows fast data collection at very low electron dose levels and has a simple experimental setup (Supplementary Fig. 2) analogous to the standard rotation method^6^ commonly used in X-ray diffraction (XRD) and neutron diffraction (ND).

Commonly applied structure refinement routines apply the kinematical approximation, which assumes that electrons are scattered by the crystal once at most and that the diffracted intensity of reflection **h** is proportional to the square of the structure-factor amplitude (*I*_**h**_ ~ |*F*_**h**_|^2^). However, inelastic scattering, defects and especially multiple elastic scattering cause deviations from this proportionality, leading to worse figures of merit (R-factors) and limited quality of the structure models^4^. The dynamical theory of ED describing multiple scattering was established almost a century ago^7^ and nowadays is implemented in many tools applying either multislice or the Bloch-wave approach to calculate, for example, high-resolution (scanning) TEM images, convergent-beam ED patterns and precession-assisted 3D ED intensities^8,9^. Basically, the theory describes how simultaneously excited beams interfere as they propagate through the crystal and predicts non-linear deviations from intensities expected by the kinematical approximation including non-zero intensities for systematically absent reflections, which is commonly observed in 3D ED experiments^10^ (Supplementary Fig. 3). These deviations depend on the crystal thickness, its orientation and the structure factors of all interfering beams. A valuable side effect is the intrinsic sensitivity of the dynamical intensities to the absolute structure of non-centrosymmetric crystals^11^. This geometric property is of highest relevance for catalysis and pharmacology, for example, because it is directly related to the absolute configuration of chiral molecules forming the crystal.

Despite several successful demonstrations^12–14^, the analysis of dynamical effects in ED patterns is not yet considered a routine approach for the reliable determination of absolute structure. Furthermore, the computation of integrated dynamical diffraction intensities has never been reported for continuous-rotation 3D ED data and therefore structure refinements on these data were limited by the kinematical theory of diffraction, which removes any information on the absolute structure.

In this work, we present a data reduction and dynamical refinement routine for the analysis of all commonly applied 3D ED measurement protocols (see Supplementary Fig. 2 for their systematic overview). No modification of established experimental procedures is needed, and the method can thus also be applied to existing experimental data. Based on both new and previously published 3D ED data sets of 13 organic and 6 inorganic compounds, we demonstrate the power of this method for routine structure analysis. We show the straightforward determination of the absolute structure based on experimental data from 58 crystals of 9 different chiral compounds. Furthermore, the method provides improved fits to the data, significantly improved accuracy of refined atomic positions and large reduction of noise in the Fourier maps, allowing better distinction of fine structural features, such as hydrogen atoms.

## Results and discussion

### Calculation of integrated intensities

**Extended Data Fig. 1:**
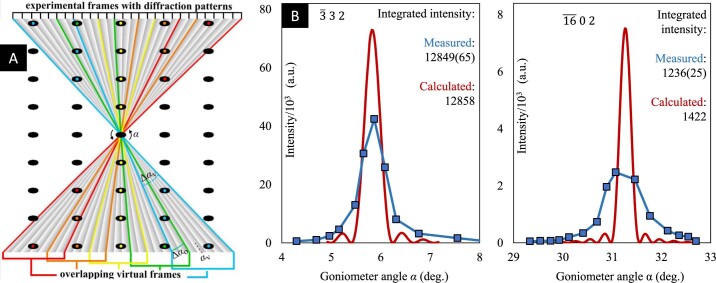
Virtual frames. (**a**) Combination of experimental frames into overlapping virtual frames. In this illustration, seven frames contribute to one virtual frame, which overlap by two experimental frames. Reflections are marked by a dot with the colour of the corresponding virtual frame to which the reflection is assigned. **(b**) Experimental (blue) and calculated (red) rocking curve of reflections −3 3 2 and −16 0 2 of mordenite. Though experimental and calculated rocking curve shapes differ substantially due to the crystal mosaicity, integrated intensities are in good agreement. Source data

Experimental intensities in a single static ED pattern are very difficult to model because of their non-linear dependence on the crystal shape, mosaicity, bending, defects and, in particular, the exact crystal orientation relative to the primary beam. Therefore, refinements need to be based on integrated intensities determined by integration of the intensity profile as a function of the scattering vector, also referred to as rocking curve. Dynamical intensities in our method are calculated for a finite number of orientations assuming a perfect block-shaped crystal. The influence of different shapes was tested by assuming certain thickness-distribution functions^15^, but this substantially increases the computational cost and in our tests had only a negligible effect on the final result. The range of experimental crystal orientations is subdivided into a set of so-called overlapping virtual frames (OVFs, Extended Data Fig. 1a). Calculated integrated intensities are then determined by numerical integration of the corresponding rocking curve (Extended Data Fig. 1b). This approach is the crucial methodological development that made the dynamical refinement applicable also to continuous-rotation 3D ED data. The details of the generation of OVFs are given in Methods.

### Improved models with dynamical refinement

To evaluate the performance of the method, we analysed in detail the crystal structures of α-quartz; albite; the zeolite structures mordenite, natrolite and STW_HPM-1; the framework compound cobalt aluminophosphate (CAP); the MOF CAU-36; in situ crystallized α-glycine; the pharmaceutical compounds carbamazepine, (+)-limaspermidine and in situ crystallized abiraterone acetate; and the methylene blue derivative MBBF4 (Table 1). We compare the results with the kinematical refinements performed on the same data. To ensure a fair comparison, we benchmarked our kinematical results against refinements obtained with other established software packages (Supplementary Table 3).

**Table 1:** Sample overview

Data source	Compound	Empirical formula	Space group	*V* (Å^3^)	*T* (K)	Detector	*N*_data_
This work	α-Quartz	SiO_2_	*P*3_2_21	113.3	293	CCD	1
Ref. ^19^	Albite	NaAlSi_3_O_8_	*P-*1	324	293	HPD	3
Ref. ^31^	Mordenite	SiO_2_	*Cmcm*	2,850.4	293	HPD	1
This work	Natrolite	Na_2_Al_2_Si_3_O_10_(H_2_O)_2_	*Fdd*2	2,251.7	293	CCD	1
This work	STW_HPM-1	(C_8_N_2_H_15_)F|[Si_10_O_20_]	*P*6_1_22	3,642.6	100	CMOS	1^a^
This work	CAP	Co_1.14_Al_2_P_4_O_20_H_10.72_	*P*2_1_/*n*	712.0	100	CCD	3
Ref. ^16^	CAU-36	C_56_H_48_Co_2_N_8_NiO_18_P_4_	*P*-42*c*	4,214.8	100	HPD	4
Ref. ^24^	α-Glycine	C_2_H_5_NO_2_	*P*2_1_/*n*	304.7	100	HPD	1
Ref. ^32^	Carbamazepine	C_15_H_12_N_2_O	*P*2_1_/*n*	1,144.8	100	CMOS	2
Ref. ^32^	(+)-Limaspermidine	C_19_H_26_N_2_O	*P*2_1_2_1_2_1_	1,594.4	100	CMOS	2
This work	Abiraterone acetate	C_26_H_33_NO_2_	*P*2_1_2_1_2_1_	2,186.0	100	CCD	5
Ref. ^26^	MBBF4	C_90_H_91_B_4_F_16_N_21_S_3_	*C*2/*c*	8,973.0	293	HPD	8

*V* is the unit cell volume, *N*_data_ is the number of measured data sets. HPD, hybrid pixel detector (also known as direct electron detector). CAP samples were measured with static 3D ED, all other compounds with continuous-rotation 3D ED.

^a^Another sample of STW_HPM-1 was measured and analysed independently at *T* = 293 K (HPD detector) as a control test for the absolute structure determination.

We also compared the present method with dynamical refinements against precession-assisted 3D ED data of α-quartz and natrolite. The comparison shows that obtained structural models are of similar quality. However, detailed analysis indicates that continuous-rotation 3D ED is more suitable for beam-sensitive materials and requires shorter computing times than precession-assisted 3D ED (Supplementary Fig. 7, Supplementary Table 4).

As the data processing is different for dynamical and kinematical refinement, we also made sure that the observed improvements are indeed due to the inclusion of the dynamical refinement and not because of the differences in data processing. We developed a method, ‘frame-based kinematical refinement’ (Supplementary Information), for performing dynamical and kinematical refinements on exactly the same data. The comparison of the results shows that the observed improvement can indeed be predominantly attributed to the better description of dynamical effects (Supplementary Fig. 8, Supplementary Table 5).

**Fig. 1:**
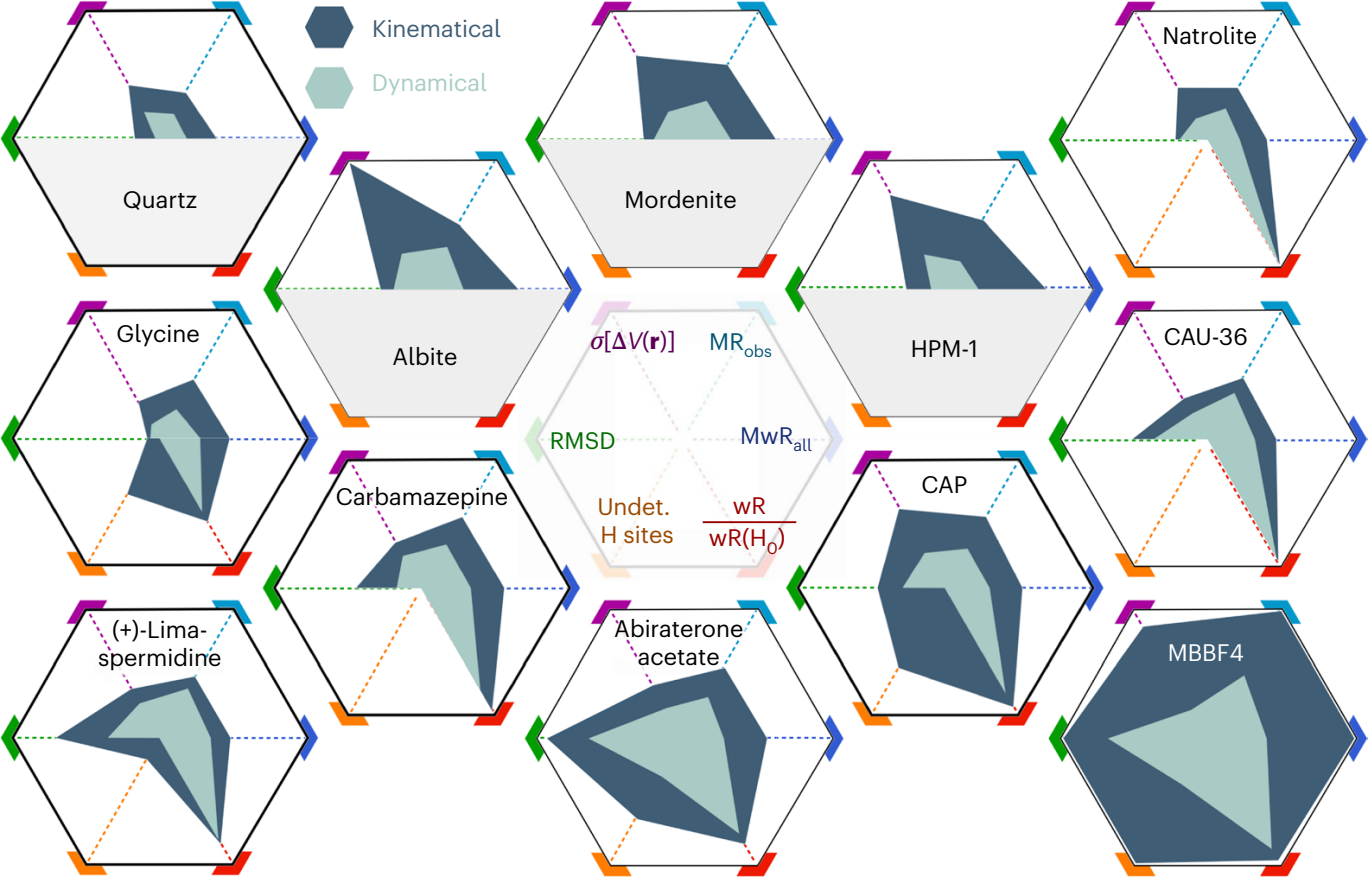
Comparison of kinematical and dynamical refinements. Radar charts of key results of kinematical (dark teal blue, outer polygon) and dynamical (pale green, inner polygon) refinements. Smaller values closer to the centre are better. *σ*[Δ*V*(**r**)] is the standard uncertainty of the difference Fourier map (purple; range: 0–0.372 e Å^–1^), MR_obs_ (light blue; range: 0–0.294) and MwR_all_ (dark blue; 0–0.333) are the respective R-factors, RMSD is the root-mean-square deviation of the bond lengths (green; 0–0.603 Å), wR/wR(H_0_) is the ratio of wR_all_ of the refinement with and without hydrogen atoms (red; 0–0.997), ‘undet. H sites’ refers to the fraction of hydrogen atoms that could not be confirmed from difference Fourier maps (orange; 0–0.891). The latter two parameters are not applicable to quartz, albite, mordenite and STW_HPM-1. Source data

In the following sections we illustrate the superiority of the dynamical refinement approach over the kinematical approach by comparing agreement factors (R-factors), the overall accuracy of bond lengths in the refined models and the noise level in Fourier maps (Fig. 1, Supplementary Tables 6 − 17). Furthermore, we demonstrate that the dynamical refinement allows a straightforward determination of the absolute structure based on the diffraction data alone, which is impossible within the kinematical approximation as the calculated intensities of Bijvoet pairs are identical.

**Extended Data Fig. 2:**
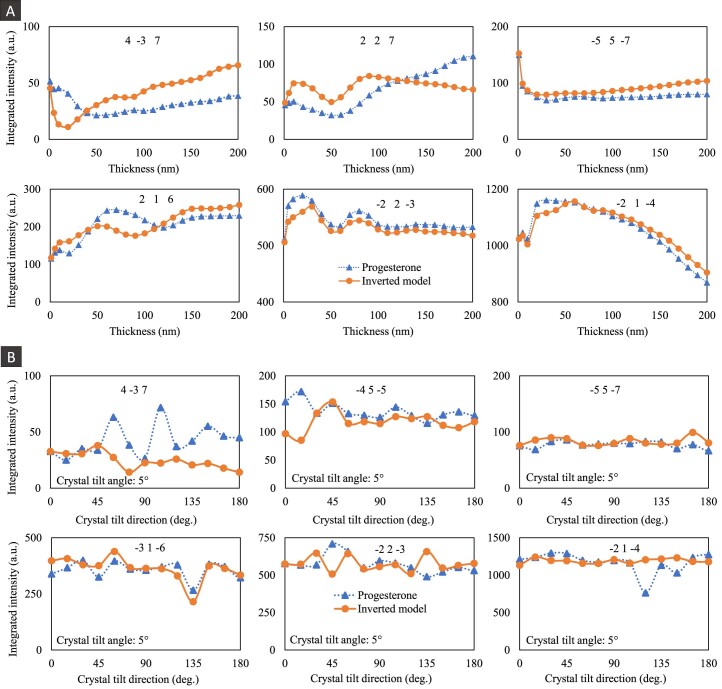
Non-linear behaviour of calculated intensities with thickness and orientation. Illustration of non-linear dependence of calculated intensities on modelled crystal thickness and crystal orientation relative to the primary beam applying the dynamical theory of diffraction. Reflections are assigned to the same virtual frame and integrated intensities were normalised so that the average intensity of all reflections of the virtual frame is 100. Calculations based on crystal structure model of progesterone (blue) and the inverted model of progesterone (orange) without refining any parameter. (**a**) Calculated (integrated) intensities for six reflections of progesterone as a function of the thickness. (**b**) Calculated (integrated) intensities for six reflections of progesterone as a function of the crystal orientation. Here, the rotation axis is tilted by 5° into a direction given by the crystal tilt direction. Source data

The most apparent improvement of applying dynamical refinement is the reduction of the R-factors (typically by a factor of around two). Note that symmetry-averaging of reflections within a single data set or across multiple data sets is not possible in the dynamical refinement, because calculated intensities non-linearly depend on, for example, the crystal shape and the geometric relationship between the primary beam and measured reflection (Extended Data Fig. 2). This, however, does not prevent a dynamical refinement against a combination of data sets. To allow for an unbiased comparison between the dynamical and kinematical refinement, we perform symmetry-averaging of experimental and calculated intensities after the refinement and calculate refinement figures of merit based on these averaged intensities. We refer to these values as MR-factors (see Methods and Supplementary Information for details). They are the best dynamical equivalent to the standard R-factors crystallographers are used to.

We evaluated the model accuracy using the root-mean-square deviation (RMSD) of refined covalent bond lengths from the respective reference values for all non-hydrogen atoms. The RMSD was in all cases lower for the dynamical refinement, on average by a factor of 1.5 (Fig. 1).

**Fig. 2:**
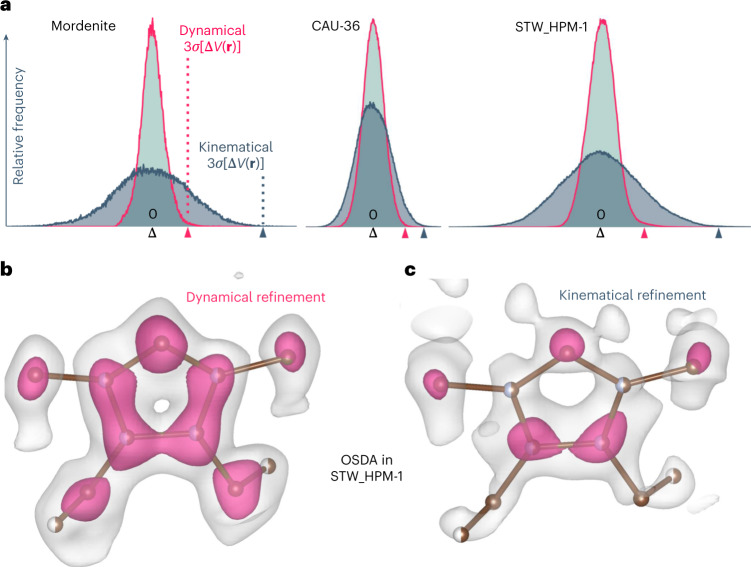
DESP maps. **a**, Histogram of DESP maps Δ*V*(**r**) of selected kinematical (grey) and dynamical (pink) refinements. The bins (from left to right) that contain the values Δ*V*(**r**) = 0 e Å^–1^ (black), 3*σ*[Δ*V*(**r**)] of the dynamical refinement (pink) and 3*σ*[Δ*V*(**r**)] of the kinematical refinement (blue) are marked on the horizontal axis. Each bin has a width of 0.0033 e Å^–1^. **b**,**c**, DESP map from dynamical (left) and kinematical (right) refinement of STW_HPM-1 calculated after the refinement without the organic structure-directing agent (OSDA) guest molecule 2-ethyl-1,3,4-trimethyl-imidazolium. Overlay of the structure of the guest molecule is shown as a visual guide. Occupancy of C (brown) and N (light blue) atoms indicated by half (50%) or full spheres (100% occupancy). Isosurface levels are shown at 2*σ*[Δ*V*(**r**)] (white) and 4*σ*[Δ*V*(**r**)] (pink). Source data

The correctness and completeness of a model is commonly inspected using difference Fourier maps of the electrostatic potential Δ*V*(**r**). Compared with kinematical refinements, the dynamical refinement based on the same experimental data reduced the noise level of Δ*V*(**r**) by a factor of 1.5 to 4 (Figs. 1 and 2, Supplementary Tables 6–17). Hence, weak structural features are more easily observed in difference maps obtained by dynamical refinement. This implies an easier detection of hydrogen atoms. Furthermore, it allows a more reliable and precise location of guest molecules in zeolites and MOFs. We illustrate the improvement with two examples, a cobalt MOF, CAU-36 (ref. ^16^), and a chiral zeolite, STW_HPM-1 (ref. ^17^), which is a pure silica zeolite with STW topology, first discovered as germanosilicate SU-32 (ref. ^18^). In both cases, the dynamical refinement provided significantly improved difference electrostatic potential (DESP) maps (Fig. 2, Supplementary Fig. 9). In STW_HPM-1 the dynamical refinement allowed an unambiguous detection of all individual atoms of the guest molecule (Fig. 2b), while the molecule was much less well defined in the kinematical result (Fig. 2c). In CAU-36, the dynamical DESP map allowed the location of a second orientation of the guest molecule, with the occupancy subsequently refined to 5.9(4)% (Supplementary Fig. 9).

Another interesting case is the mineral albite. In a previous study using kinematical refinement, 16 data sets were combined to identify the correct out of 16 possible Al/Si distribution patterns exclusively from the R-factors^19^. Using dynamical refinement, the reinvestigation of only 3 of the 16 data sets achieves a clearer identification of the correct element assignment from a more pronounced difference in R-factors between the candidate structures and from the analysis of the displacement parameters (Supplementary Figs. 10 and 11).

### Hydrogen atoms

**Fig. 3:**
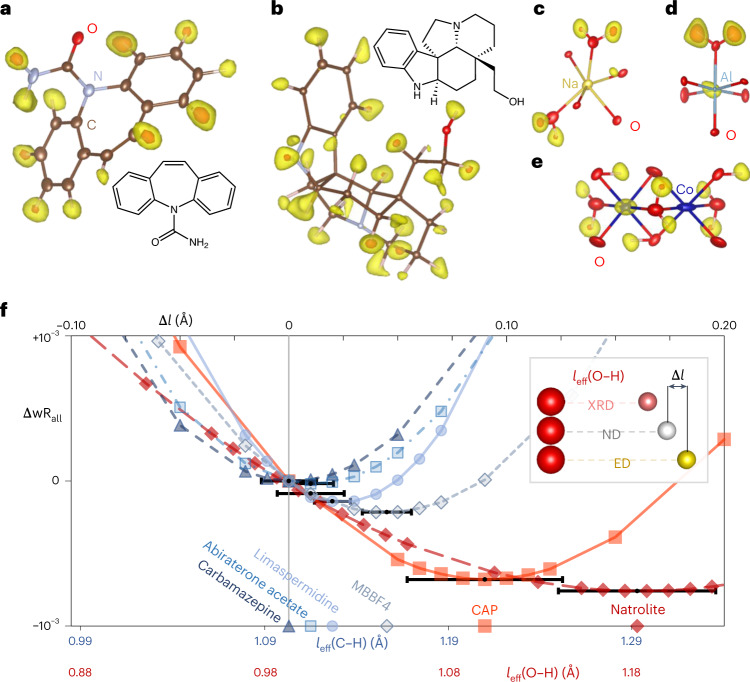
Visibility and position of hydrogen atoms in electrostatic potential maps. **a**–**e**, Difference potential maps of carbamazepine (**a**), (+)-limaspermidine (**b**), the vicinity of the Na site in natrolite (**c**), the vicinity of the Al site in CAP (**d**) and the vicinity of Co sites in CAP (**e**) based on the final models with hydrogen atoms removed. Yellow isosurfaces correspond to the 3*σ*[Δ*V*(**r**)] level, orange isosurfaces to the 5*σ*[Δ*V*(**r**)] level. The maps are superimposed with structure models with expected locations of hydrogen sites indicated by pale-pink lines from the respectively bonded C or O atom. Anisotropic displacement ellipsoids shown at the 50% probability level. **f**, Dependence of wR_all_ on the C–H and O–H distances. Δ*l* is the difference between the constrained and the internuclear distance^33^. Datapoints on the horizontal axis indicate the optimized difference of the apparent bond length that minimizes wR_all_. Horizontal error bars (3*σ*) are plotted at the optimal Δ*l* for each compound. At the bottom, effective internuclear distances for C–H and O–H used in the respective refinements are indicated. Source data

A low noise level is also a prerequisite for the successful detection of the weakest scatterers (Fig. 3). Although the observation of hydrogen atoms in difference Fourier maps has been reported from kinematical refinements, it is by no means routine (Supplementary Figs. 12–15). An illustrative example is the disordered framework structure CAP with partially occupied Co and H sites. The difference ESP map from dynamical refinement revealed all seven hydrogen positions with occupancies ranging from 0.5 to 1.0 as maxima larger than 4*σ*[Δ*V*(**r**)]. Another hydrogen site with an expected occupancy of about 0.28 is detected close to the 3*σ* level (Supplementary Information). Likewise, hydrogen atoms were easily detected in the organic compounds. All the 5, 12 and 26 hydrogen sites were clearly detected above 3*σ*[Δ*V*(**r**)] in the dynamical refinements of α-glycine, carbamazepine and (+)-limaspermidine, respectively (Fig. 3d–f). In the cases of the more complex structures of abiraterone acetate and methylene blue derivative (MBBF4), most H atoms were still found (Fig. 1).

In the young history of 3D ED, the constraints on hydrogen atoms in the refinement were mostly based on bond length statistics derived either from XRD or ND (Supplementary Fig. 17). Even though the electrostatic potential is dominated by the positively charged nuclei, chemical bonds on average shift the negative potential of an electron and thus cause a shift of the observed overall potential away from the bond. Within the independent atom model, we thus expect that interatomic distances involving hydrogen atoms are longer than the true internuclear distance. Recently, this was briefly indicated in a cryo-EM single-particle study^20^, but a quantitative evaluation of this effect has not yet been done. The accuracy and sensitivity of the dynamical refinement provides the means to analyse the hydrogen positions. We performed a series of dynamical refinements varying the constrained C−H, N−H and O−H distances and compared the corresponding wR_all_ (Fig. 3f). At *T* = 100 K, the C−H distances in carbamazepine, abiraterone acetate and (+)-limaspermidine are less than 0.03 Å longer than the respective internuclear distances. This difference is in excellent agreement with a theoretical analysis of the observed bond lengths within the independent atom model (compare with figure 13 in ref. ^21^). At room temperature, the C−H distances in MBBF4 are longer by 0.05 Å. The O−H distances in CAP (*T* = 100 K) are longer by about 0.09 Å and in natrolite (*T* = 293 K) by 0.15 Å. The refinements thus reveal a clear trend towards longer refined bond lengths with a deviation that increases with the polarity of the covalent bond and the temperature, which thus reveals the limitations of the independent atom model.

### Determination of absolute structure

Dynamical diffraction theory predicts a strong sensitivity of diffracted intensities to the absolute structure because of the interference of multiple excited beams. For example, the calculated intensities of (+)-limaspermidine and (−)-limaspermidine differ by 11.7% (first quartile) to 61.1% (third quartile) (Supplementary Fig. 18). Even though inelastic scattering and crystal imperfections tend to diminish these differences, they still remain strong. As a control test of the presented method, the chiral zeolite STW_HPM-1 was chosen as a suitable case study because crystals grown from the same batch are chiral, with 50% crystallizing in space group *P*6_1_22, and 50% in *P*6_5_22 (racemic conglomerate). A relatively large, random sample was chosen for single-crystal XRD measurement. Subsequently, the same crystal was crushed and prepared for continuous-rotation 3D ED (Supplementary Fig. 19g). Dynamical refinements of the two possible enantiomorphs converged to wR_all_ of 0.306 (space group *P*6_1_22) and 0.234 (space group *P*6_5_22). The latter, with the significantly lower R-factors, was then confirmed as the correct enantiomorph by the determination of the Flack parameter of 0.06(2) from single-crystal XRD data (Supplementary Tables 18 and 19).

**Fig. 4:**
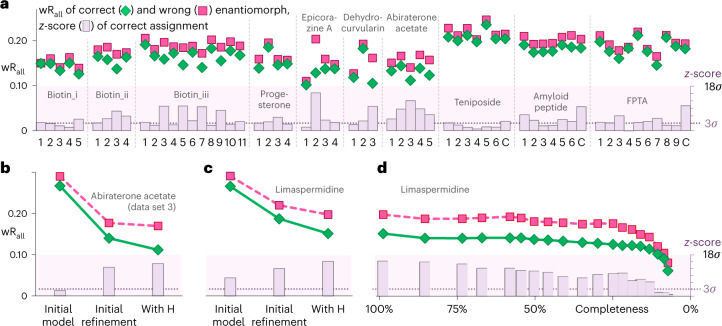
Confidence and robustness of absolute structure determinations. wR_all_ of the dynamical refinement of the correct enantiomorph (green diamonds) and wrong enantiomorph (pink squares). *z*-score (purple bars) expresses the confidence of the absolute structure assignment. The dotted purple line indicates the 3*σ* limit (99.7% probability). **a**, Absolute structure assignment of 56 crystals from 8 different organic compounds (Extended Data Fig. 3). Biotin_i, biotin_ii and biotin_iii refer to three sets of data measured in three laboratories (refs. ^34–36^). Numbers under the horizontal axis label individual data sets. The label C refers to the wR_all_ and *z*-score obtained from a combination of all data sets from the same source. **b**,**c**, Absolute structure assignment based on one data set of abiraterone acetate (**b**) and limaspermidine (**c**) as a function of structure determination step. ‘Initial model’: unrefined model based on coordinates from structure solution. ‘Initial refinement’: coordinates and isotropic displacement parameters refined. ‘With H’: includes constrained hydrogen atoms. **d**, Absolute structure determination of limaspermidine for refinements with gradually decreased completeness. Source data

**Extended Data Fig. 3:**
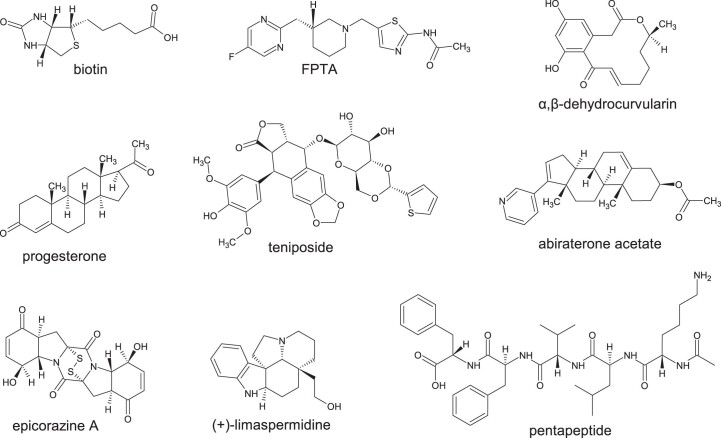
Structural formulas of molecular compounds used for absolute structure determination. Structural formulas of the 9 molecules relevant for the demonstration of absolute structure determination.

As a demonstration of the robustness of the method and applicability to high-throughput routine determination of absolute structure, we investigated—in addition to (+)-limaspermidine and abiraterone acetate—all continuous-rotation 3D ED measurements of chiral non-protein structures publicly available in the 3D ED/MicroED community on raw-data repository Zenodo (Extended Data Fig. 3). Dynamical refinements of both enantiomorphs were carried out against individual data sets of (+)-limaspermidine (1 crystal), abiraterone acetate (5 crystals), (+)-biotin (20 crystals), progesterone (4 crystals), epicorazine A (4 crystals) and α,β-dyhydrocurvularin (3 crystals), each with a completeness ranging from about 40% to 90% and a resolution in the range 0.77 to 1.43 Å (Supplementary Table 20). Refinements against a combination of data sets were performed for teniposide (Supplementary Table 21), an amyloid peptide fragment (Supplementary Table 22), and (*R*)-*N*-(5-((3-((5-fluoropyrimidin-2-yl)methyl)piperidin-1-yl)methyl)thiazol-2-yl)acetamide (Supplementary Table 23). The latter is a triclinic pseudo-centrosymmetric structure with *Z*′ = 2 (hereafter abbreviated as FPTA)^22^. In all 58 cases, without exception, a simple comparison of the R-factors indicated the correct enantiomorph as the wR_all_ of the model with wrong handedness is on average by a factor of 1.13 higher than the wR_all_ of the correct model. In the cases of epicorazine A and α,β-dyhydrocurvularin, the absolute structure assignment of this study was confirmed by XRD experiments by Novartis, who provided the samples. For other samples, their absolute structure was known in advance. To quantitatively assess the reliability of the assignment of the enantiomorph, we adopted an approach inspired by the work of Le Page et al.^23^ (Supplementary Information). The analysis provides both the confidence level in terms of *σ* of the normal distribution (*z*-score) as well as the probability that the estimation of the absolute structure is correct. The absolute structure assignments typically achieved a confidence level of 3.4*σ* (median), with an average over all data sets of 4.3*σ*. Confidence levels are often also high for incomplete low-resolution data sets (for example, data set 4 of progesterone or data set 1 of dehydrocurvularin), but inferior data sets that additionally show reflection splitting tend to give a lower *z*-score and in one case yielded a null result (FPTA 4). Importantly, the dynamical refinement never favoured the wrong enantiomorph (Fig. 4, Supplementary Tables 20–24). Determination of the Flack parameter through the refinement of the inversion twin fraction was tested, but the refined twin fraction and its uncertainty were less conclusive than the approach presented above. Many parameters influence the refinement of the twin fraction, including precise orientation angles of the OVFs, which in our implementation are optimized based on the final input model and handedness. A reliable determination of the Flack parameter will be the subject of further development. The currently presented approach can be reliably applied to enantiopure materials.

Recently, the potential of 3D ED for polymorph screening of in situ grown molecular crystals has been investigated^24^. With a similar approach, crystals of abiraterone acetate were grown directly on a TEM grid from an aqueous solution of abiraterone acetate citrate. Crystals diffracted up to a resolution of about 1 Å at a temperature of *T* = 100 K. Continuous-rotation 3D ED experiments were performed at different spots of selected crystals, which all showed signs of strong mosaicity (Supplementary Fig. 19e,f). The identification of the correct enantiomorph was investigated at different stages of the structure determination for one of the data sets. The unrefined initial model based solely on the structure solution already identified the correct enantiomorph at the 2.4*σ* confidence level. The subsequent refinement first without H atoms and then with constrained H atoms further increased the confidence level to 12.5*σ* and 14.1*σ* (Fig. 4b, Supplementary Table 20). Likewise, the absolute structure of (+)-limaspermidine was determined based on the unrefined model from the structure solution at 7.9*σ* level (Fig. 4c). Note that the strongest scatterer of abiraterone acetate and (+)-limaspermidine are O atoms. As the computational cost of the dynamical calculations scales with the number of refined parameters, the calculations for the initial absolute structure determination without refinement of structure model take only a few minutes with a standard personal computer (Supplementary Table 4), making the determination of absolute structure in these cases very quick.

In a further test, the robustness of the absolute structure determination of (+)-limaspermidine was investigated by gradually decreasing the number of OVFs used in the refinement, thus decreasing the data completeness. In the completeness range 100% to 25% the observed R-factor difference is almost constant around 0.05, while the confidence level for the enantiomorph identification gradually decreases from 15.1*σ* to 9.7*σ*. Even with a completeness of only 13%, corresponding to an effective goniometer rotation of 9.2°, the refinement with constrained displacement parameters is stable and correctly identifies the enantiomorph at the 6.1*σ* level (Fig. 4c, Supplementary Tables 24).

This analysis illustrates that 3D ED combined with dynamical refinement is a very reliable method for absolute structure determination. Furthermore, among the available methods to determine the handedness or chirality of single nanocrystals in a TEM^12,13,22,25^, the presented approach has to the best of our knowledge the least requirements in terms of TEM hardware, sample preparation and crystallite orientation as, for example, no alignment of zone-axis patterns is needed and modern 3D ED setups measure full data sets in only a few minutes^26,27^. With further automation, the presented approach is suitable for routine and high-throughput applications for all kinds of enantiopure compounds without the need for a time-consuming and potentially expensive growth of larger single crystals. We argue that 3D ED may also be considered an attractive alternative to the established XRD-based approach even if single crystals of appropriate size are available, because the requirements for a successful determination in terms of crystal quality, measurement conditions, resolution, completeness and elemental composition are strikingly reduced. This is especially relevant for pharmaceuticals, if the absolute structure determination by XRD is inconclusive or challenging^28,29^. However, if multiple limiting factors are combined, that is, low completeness together with low resolution and inferior crystal quality, the absolute structure determination by 3D ED may also yield a null result.

## Conclusions

A new data reduction and structure refinement procedure based on the concept of virtual frames was developed for static and continuous-rotation 3D ED data collection methods like ADT, RED, cRED and MicroED. Various structural features of a broad range of 19 compounds, including minerals, zeolites, molecular crystals, a MOF, several pharmaceutical compounds and an amyloid peptide, were studied applying the dynamical theory of ED. A comparison with the commonly applied kinematical refinement revealed that in all cases the dynamical refinement gives significantly improved results. Alongside better agreement factors, the benefits include an overall enhanced accuracy of atomic positions, decreased level of noise in the DESP maps and clearer detection of hydrogen atoms as well as guest molecules. The improved sensitivity of the analysis of H atoms showed that, within the independent atom model, the commonly applied constraint schemes in electron crystallography do not fit to the ED data. This observation must be considered for accurate modelling of H atoms.

In the case of chiral structures, the dynamical refinement easily identifies the correct enantiomorph by a simple comparison of corresponding R-factors, which is decidedly lower when refining the correct absolute structure. A thorough analysis of 58 data sets of molecular crystals with the correct identification of the known absolute structure establishes dynamical refinement and 3D ED as a reliable and generally applicable tool for the investigation of the absolute configuration of molecules forming submicrometric and nanosized crystals.

Our procedure for structure determination is compatible with all available 3D ED data collection methods. In total, 79 data sets from 10 different microscopes and from various laboratories were investigated. This work thus makes an accurate and deeper structure analysis accessible to a broad range of electron crystallography laboratories. As such this work is an important step towards closing the gap in the quality and accessibility between 3D ED-based and XRD-based structure determinations. Nevertheless, this gap is evidently not fully closed yet, as can be seen from the still relatively high R-factors and related lower accuracy with respect to XRD results. However, we are convinced that it is possible to narrow this gap even more by taking into account yet unmodelled aspects like inelastic scattering^30^, crystal imperfections or the crystal shape. The dynamical refinement method presented in this work forms a basis for further investigation of these effects and for further improvement of the quality of structure analysis from 3D ED data.

## Methods

### Experiments

#### Sample preparation

Samples of α-quartz were provided by the authors of ref. ^37^, in which the synthesis is described. A TEM sample grid (Cu square grid G300 from ‘SPI supplies’ with holey carbon film) was prepared by softly pressing the grid against the nanocrystalline white powder using a pair of tweezers. A natural natrolite sample from Marianska skala, Usti nad Labem, Czech Republic, was first crushed in a mortar before the grid was prepared in an analogous manner. The sample of CAP was prepared like the natrolite sample and originates from the same batch as the crystallites used in a previous study^38,39^.

A batch of chiral zeolite crystals STW_HPM-1 was synthesized using 2-ethyl-1,3,4-trimethylimidazolium as the structure-directing agent according to a previously reported procedure^17^ with some modifications. While the final product of STW_HPM-1 is racemic, each crystal (≈50 μm in size) is a single enantiomorph. One crystal was used for single-crystal XRD, and the same crystal was subsequently dismounted by dissolving the attached paraffin wax in acetone. The isolated crystal was crushed and ground between two glass slides before carefully inserting a copper EM grid covered by holey carbon film to collect the ground crystal fragments.

Another crystal of HPM-1 was crushed, dispersed in absolute ethanol, treated by sonication for 1 minute and then transferred onto a copper EM grid covered by a holey carbon film. The TEM grid was plunge frozen in liquid ethane cooled by liquid nitrogen before cryo-transferring them to a Titan Krios using an autoloader.

#### Microscope synchronization, automation and control

The microscope used for the measurements of α-quartz, natrolite, CAP and abiraterone acetate was an FEI Tecnai G2 20 (LaB_6_ filament, 200 kV acceleration voltage) equipped with a charge-coupled device (CCD) camera (SIS Veleta) and a DigiSTAR precession unit from Nanomegas. The goniometer tilt, beam blanking and exposure of the CCD detector were synchronized similar to a previously reported approach^40^ by accurately determining the respective initialization times and the angular velocity of the goniometer rotation. A crystal-tracking routine analogous to Fast-ADT^41^ was also implemented for an optimized automation of the data acquisition. The microscope and detector were controlled by scripts within the detector software iTEM.

#### Continuous-rotation and precession-assisted 3D ED measurements of quartz and natrolite

Each TEM grid was carefully placed and fixed on a single tilt sample holder and inserted into the sample stage of the TEM. With a promising nanocrystal in the field of view (Supplementary Fig. 19a,b), a series of 21 TEM images was recorded to track the crystal position in *α* steps of 5° across the range −50° to +50°, from which the idealized movement of the crystal during the rotation of the goniometer was derived by interpolation. Diffraction patterns were recorded sequentially. Each sequence step starts with the recording of a diffraction pattern with beam precession (Supplementary Tables 6 and 8). After that, the CCD recorded the next diffraction pattern (without beam precession) while the *α* angle slowly rotates by defined steps of 1.0° for quartz and 0.6° for natrolite. Without changing to image mode, the step finishes by adapting the beam position according to the expected crystal position determined by the tracking procedure.

#### Continuous-rotation 3D ED measurement of abiraterone acetate

Five crystals (Supplementary Fig. 19e,f) were measured at *T* = 100 K with a single tilt cryoholder. The crystals were tilted in steps of 0.4° with a tilt speed of 0.465° s^–1^ while the diffraction patterns were recorded for 844 ms, and the goniometer stopped for the CCD readout and initialization of the next step.

#### Static 3D ED measurement of CAP

Two crystals (Supplementary Fig. 19c,d) were measured at *T* = 100 K using the automated data acquisition routine with crystal tracking using a single tilt cryoholder. Static diffraction patterns were exposed for 350 ms and separated by fine mechanical steps of Δ*α* = 0.1°. The microscope synchronization was not used so that during the detector dead time of about 621 ms the measured crystals were exposed to the electron beam. Hence, only 36% of the effective exposure time were used to record the diffraction patterns.

#### Single-crystal XRD and 3D ED on STW_HPM-1

Single-crystal XRD and continuous-rotation 3D ED data were collected on the same crystal. The XRD measurement of an STW_HPM-1 single crystal with a diameter of 50 µm was performed with a Bruker D8 Venture four-circle diffractometer (Cu Kα radiation, PHOTON II detector) and analysed with the software APEX3. Structure solution and refinement were performed with SHELXT and SHELXL^1^ in space group *P*6_5_22. The space group and absolute structure were confirmed by the refined Flack parameter of 0.06(2). The 3D ED experiments were performed at room temperature on a fragment of the crushed crystal from the XRD measurement with a JEOL JEM-2100 transmission electron microscope operated at 200 kV equipped with a Timepix detector (Amsterdam Scientific Instruments) using Instamatic software^42^. The data were collected by continuously rotating the crystal at a constant speed of 0.46° s^–1^. An exposure time of 0.5 s per frame was used, so that each frame covers an angular range of 0.23°. Another sample was measured at low temperature (100 K) on a Titan Krios G3i equipped with a CETA-D detector (CMOS, complementary metal-oxide-semiconductor) by using software EPU-D. The data were collected by continuously rotating the crystal at a constant speed of 0.6° s^–1^. An exposure time of 0.5 s per frame was used. An extremely low electron dose was applied to minimize possible beam-induced movement of the guest molecules. The entire data set was collected with an accumulated electron dose of less than 5 e^−^ Å^–^^2^. This measurement was used for detailed structure analysis, especially of the guest molecules in the pores.

### Data analysis

#### Overlapping virtual frames

Each OVF is characterized by its average goniometer angle *α*_v_ and the angular range Δ*α*_v_ covered by the virtual frame (Extended Data Fig. 1a). Dynamical calculations based on the structure model are then performed for dozens of crystal orientations covering the angular range of the OVF, resulting in idealized rocking curves for all reflections assigned to the OVF. Numerical integration of these rocking curves then yields the model integrated intensities *I*_calc_ for each OVF. However, for reflections that are in exact diffraction condition close to one of the limiting Ewald spheres only incomplete rocking curves are calculated. Such partial intensities should not be used in the refinement and geometric filters are used to exclude them^15^. Only integrated intensities of reflections with a minimum distance *D*_Sg_ from the limiting Ewald spheres and with a small ratio *R*_Sg_ = |*S*_g_|/(*D*_Sg_ + |*S*_g_|) are considered in the refinement, where *S*_g_ is the excitation error of a reflection relative to the average goniometer position of the respective OVF (Extended Data Fig. 1a). To include in the refinement as many measured reflections as possible, the *α* offset between two consecutive OVFs must be smaller than Δ*α*_v_ so that there is an overlap Δ*α*_o_ corresponding to at least one experimental frame between them (Extended Data Fig. 1a, Supplementary Tables 1 and 2). Typical values for Δ*α*_v_ used in this study are 1.5° to 3°, achieved by combining between 2 and 50 experimental frames to one virtual frame, with an overlap Δ*α*_o_ between 0.5° and 1°. Optimal values of *R*_Sg_^max^ are typically between 0.5 and 0.8, and appropriate values of *D*_Sg_^min^ are between 0.0015 and 0.0030 Å^−1^ (Supplementary Fig. 4). The concept of virtual frames ensures that both experimental and calculated intensities are properly integrated.

#### Data processing of data sets from former studies

Data sets of albite^19^, amyloid peptide fragment^43^, biotin^34–36^, CAU-36^16^, α,β-dehydrocurvularin^44^, epicorazine A^44^, mordenite^24^, MBBF4^26^, progesterone^36^, teniposide^36^ and (*R*)-*N*-(5-((3-((5-fluoropyrimidin-2-yl)methyl)piperidin-1-yl)methyl)thiazol-2-yl)acetamide^22^ were downloaded from Zenodo. Data sets of carbamazepine^32^, (+)-limaspermidine^32^ and α-glycine^24^ were obtained from the authors of the respective publications. Diffraction pattern files of carbamazepine and (+)-limaspermidine were converted to tagged image file format (TIF) with ImageJ. Diffraction patterns of MBBF4 were converted to TIF files using the ‘eiger2cbf’ and ‘FabIO’ tools^45^. Other data sets not provided in TIF format were converted to TIF format using the ‘FabIO’ package.

Diffraction patterns of α,β-dehydrocurvularin and epicorazine A were flipped along the vertical axis before processing because the raw detector images were not written in consonance with the conventional geometry (Supplementary Information).

#### Data reduction

Each data set was processed separately using PETS2 software^3^. The most important steps comprise the peak search, determination of the orientation matrix, optimization of the frame orientation and determination of integrated reflection intensities. The detector distance (camera length) was adapted so that the unit cell volume agrees with the expected unit cell volume found in the literature. Frame scales were optimized based on the Laue class of the crystal and applied to the intensities in the list of reflections used for structure solution and kinematical refinement. The program also applies a Lorentz correction. Multiple output reflection files of the same compound were scaled and merged with an in-house program that minimizes the internal R-factor *R*_int_. Reflection intensities *I*_obs_ that were symmetrically equivalent within the corresponding point group were averaged and assigned new uncertainties $\sigma \left( {{\bar{I}}_{{{{\mathrm{obs}}}}}} \right) = \sqrt {\frac{{{\sum} {\left( {I - {\bar{I}}_{{{{\mathrm{obs}}}}}} \right)^2} }}{{n(n - 1)}}}$ with Jana2006.

Output files for dynamical refinement based on beam precession (quartz, natrolite) were generated based on integrated intensities determined from single frames in the standard way^3^. As preparation for the dynamical refinement based on static 3D ED (CAP) and continuous-rotation 3D ED measurements (α-quartz, albite, natrolite, mordenite, STW_HPM-1, CAU-36, α-glycine, carbamazepine, (+)-limaspermidine, abiraterone acetate, MBBF4 and the additional seven compounds for absolute structure determination), the integrated intensities *I*_**h**_ from the initial data reduction were assigned to virtual frames without applying scale factors or a Lorentz correction. Parameters of each virtual frame are the average goniometer angles (*α*_v_, *β*_v_) and sum of the covered tilt range (Δ*α*_v_ = *N*_F_Δ*α*) of a series of *N*_F_ contributing experimental frames. *N*_F_ was chosen so that Δ*α*_v_ is typically between 1.2° and 3°. The number of overlapping frames *N*_O_ was in general about 0.5*N*_F_. Details for each compound are given in Supplementary Tables 6–17.

#### Structure solution and kinematical refinement

All structures of Table 1 were solved with the charge-flipping algorithm as implemented in Superflip^46^ and refinements within the kinematical approximation were performed with Jana2006^47^. Electron scattering form factors were taken from table 4.3.1.1 of the International Tables of Crystallography, Volume C^6^. The least-squares refinement of a structural model was performed in the usual way by minimizing the weighted difference between *I*_calc_ and *I*_obs_ (refs. ^15,48^). An extinction correction was applied with one refined extinction parameter^1,31^, except for STW_HPM-1, where the refinement of extinction was unstable and the extinction coefficient was fixed to give the best *I*_obs_ − *I*_calc_ plot. In the final models of albite and MBBF4 this correction had a negligible effect and therefore it was not applied. If not stated otherwise, coordinates of hydrogen atoms were constrained assuming typical atomic distances as determined by ND experiments^33^ and riding isotropic displacement parameter (factor 1.2).

#### Dynamical refinement

Dynamical refinements were performed with Jana2006 in combination with a new version of the in-house developed and publicly available program Dyngo^15^. The latter calculates intensities expected from the dynamical diffraction theory by applying the Bloch wave formalism. Furthermore, Dyngo determines derivatives of the calculated diffracted intensities with respect to the refinement parameters. Intensities and derivatives are then used by Jana2006 to build the normal equations and perform the least-squares refinement. The parametrization of the structure factor is identical for the kinematical and the dynamical refinement, but they differ in the calculation of the diffracted amplitudes which in the case of the dynamical refinement depend on the crystal shape, its geometric orientation relative to the primary beam and the structure factors (Extended Data Fig. 2). This dependence inhibits the merging of reflections that are symmetrically equivalent because their intensities are not expected to be the same. Nevertheless, several data sets can be combined within the same least-squares refinement to increase the completeness and redundancy. In the cases of albite, CAP, CAU-36, carbamazepine, (+)-limaspermidine, abiraterone acetate, MBBF4, teniposide and the amyloid peptide, one crystal structure model was refined for each compound against a combination of at least two data sets. One thickness parameter per data set and one scale factor per virtual frame were refined. Initial values of these additional parameters were estimated in one refinement cycle with other parameters of the structural model fixed to values obtained from the kinematical refinement of from the structure solution. For non-centrosymmetric structures, the inverted model was also refined and kept if R-factors improved. If not stated otherwise, hydrogen coordinates were constrained with distances taken from reference^33^ and with riding isotropic displacement parameter (factor 1.2). Before the final refinement cycles of structures in Table 1 (except for the twinned structure of CAU-36), the orientation of each virtual frame was optimized with a downhill simplex algorithm that minimizes the wR of the reflections assigned to the frame and passing the filters as a function of the correction angles^49^. If only the absolute structure was of interest (biotin, progesterone, teniposide, epicorazine A, α,β-dehydrocurvularin, amyloid peptide, FPTA), the models published alongside the data sets were used as starting model and both enantiomorphs were refined with isotropic displacement parameters without optimizing the frame orientations.

#### Absolute structure determination

The absolute structure was determined at different stages by refining both enantiomorphs in two independent refinements but against the same data set. This approach is comparable to refining a model with the Flack parameter fixed to a value of 0 or 1, respectively. Initial absolute structure determination was based on a model with correctly assigned atomic types located at the coordinates derived from the structure solution without any hydrogen atoms. Only frame scale parameters, one thickness parameter per data set and one global isotropic displacement parameter were refined. The final absolute structure determination was based on a refinement of the coordinates and displacement parameters with constrained H positions where applicable. Refinements were mostly stable against individual data sets (biotin, progesterone, epicorazine A, α,β-dehydrocurvularin, abiraterone acetate, (+)-limaspermidine) and thus (if not stated otherwise) absolute structure determinations are each based on one data set from one crystal. In the cases of teniposide, amyloid peptide, and the pseudo-centrosymmetric compound FPTA, refinements were only stable against a combination of data sets from more than one crystal. In addition to the analysis of the overall refinements, relevant parameters (R-factors, *z*-score) are also determined for each subset of reflections originating from one crystal so that the absolute structure of each measured crystal is still individually assessed.

In most cases the distinction between the correct and incorrect absolute structure can be made directly by comparing the refinement R-factors, which are usually visibly higher for the wrong enantiomorph. To obtain a more rigorous and quantitative estimate of the probability of the correct determination, we propose an approach inspired by the method due to Le Page et al.^23^. We may evaluate the probability that the better match of the experimental data by one of the enantiomorphs is only the result of random variation of the model fit by the two enantiomorphs. To avoid the sensitivity to outliers and statistical quality of the fit, we consider only the qualitative differences. Let *N* be the number of reflections in the data set and *k* be the number of reflections for which |*I*_calc,1_ − *I*_obs_| < |*I*_calc,2_ − *I*_obs_|, that is, for which the enantiomorph labelled 1 gives a better match to the observed data. If the differences are purely random, the probability of a reflection **h** better matching the first enantiomorph is 0.5, and the probability distribution of finding *k* such matches is given by the binomial distribution with *P* = 0.5:

$$P\left( k \right) = \left( {\begin{array}{*{20}{c}} N \\ k \end{array}} \right)2^{ - N}$$

This distribution can be very well approximated by the normal probability distribution *N*(*N*/2, *N*/4) if *N* is large. We can then do a standard test of the hypothesis that the value *k* obtained from the data is a sample from this distribution. The hypothesis that *k* is a sample from this distribution can be rejected at the confidence level of *zσ* if

$$\frac{{k - \frac{N}{2}}}{{\frac{{\sqrt N }}{2}}} = z$$

The value *z* is commonly referred to as *z-*score. The probability that the enantiomorph assignment is correct is then given by the value of the cumulative distribution function of the standard normal distribution Φ(*z*).

This is, however, a very conservative estimate of the probability for two reasons. First, it does not consider the magnitude of the differences, that is, how much better does one enantiomorph fit each intensity than the other. Second, due to the experimental noise, even for a perfect model we do not expect all reflections to match better the correct enantiomer, and the probability estimate needs to be adjusted for it. Here, we will not consider the first problem, because it is difficult to incorporate quantitatively due to possible effect of model imperfections. We take ignoring this effect as a safety margin that renders our estimates of the *z*-scores and probabilities conservative.

The second problem can be approximately corrected for. If the experimental uncertainty of an intensity measurement *I*_obs_ is *σ*(*I*_obs_), assuming normally distributed experimental errors, the probability that the sign of |*I*_calc,1_ − *I*_obs_| − |*I*_calc,2_ − *I*_obs_| will indicate the wrong enantiomorph due to the experimental noise (as opposed to model errors), is given by

$$1 - \Phi \left( {\frac{{\left| {I_{{{{\mathrm{calc}}}},1} - I_{{{{\mathrm{calc}}}},2}} \right|}}{{2\sigma \left( {I_{{{{\mathrm{obs}}}}}} \right)}}} \right)$$

Thus, statistically we can expect *w* wrongly indicating assignments in the data set with *N* reflections, with

$$w = \mathop {\sum}\limits_i^N {1 - \Phi \left( {\frac{{\left| {I_{{{{\mathrm{calc}}}},1} - I_{{{{\mathrm{calc}}}},2}} \right|}}{{2\sigma \left( {I_{{{{\mathrm{obs}}}}}} \right)}}} \right)}$$

This number represents the ‘background noise’. To obtain a better estimate of the real excess of *k* above the mean value *N*/2, we may compare the excess with the total number decreased by the number of statistically wrongly assigned reflections. The adjusted *z*-score is thus obtained from

$$z = \frac{{k - \frac{N}{2}}}{{\frac{{\sqrt {N - w} }}{2}}} = \frac{{2k - N}}{{\sqrt {N - w} }}$$

## Online content

Any methods, additional references, Nature Portfolio reporting summaries, source data, extended data, supplementary information, acknowledgements, peer review information; details of author contributions and competing interests; and statements of data and code availability are available at 10.1038/s41557-023-01186-1.

## Supplementary information

Supplementary Information Supplementary text, Tables 1–24 and Figs. 1–19.

Supplementary Data 1 An archive with the CIF files of all refinements (kinematical and dynamical) of compounds analysed in detail in the Article.

Supplementary Data 2 The CIF file for the X-ray reference structure of HPM_STW.

Supplementary Data 3 The CIF file for the 3D ED refinement of the amyoid peptide.

Supplementary Data 4 Source data for Supplementary Fig. 1.

Supplementary Data 5 Source data for Supplementary Fig. 4b.

Supplementary Data 6 Source data for Supplementary Fig. 5.

Supplementary Data 7 Source data for Supplementary Fig. 6.

Supplementary Data 8 Source data for Supplementary Fig. 8.

Supplementary Data 9 Source data for Supplementary Figs. 10 and 11.

Supplementary Data 10 Source data for Supplementary Fig. 16.

Supplementary Data 11 Source data for Supplementary Fig. 17.

Supplementary Data 12 Source data for Supplementary Fig. 18.

## Data Availability

Raw experimental data (quartz, natrolite, CAP, abiraterone acetate, STW_HPM-1) are available together with data reduction files for PETS2 at 10.5281/zenodo.5579792, a data repository hosted by Zenodo^50^. Relevant PETS2 output files and JANA refinement files for all compounds listed in Table 1 are available at the same repository. Source data are provided with this paper.

## Source data

Source Data Fig. 1 Source data for the spider plots in Fig. 1.
Source Data Fig. 2 Files (in xplor format) containing the electrostatic potential maps, from which the histograms in Fig. 2a were calculated.
Source Data Fig. 3 Source data for the plot in Fig. 3f.
Source Data Fig. 4 Source data for all panels of Fig. 4.
Source Data Extended Data Fig. 1 Source data for Extended Fig. 1b,c.
Source Data Extended Data Fig. 2 Source data for all plots in Extended Fig. 2.

## Code availability

All used programs are available free of charge for academic purposes and all their features reported in this Article are included in the publicly available versions. The PETS2 software can be downloaded from http://pets.fzu.cz. The software package for structure solution (Superflip) and (dynamical) refinement with Dyngo and Jana2006/Jana2020 can be downloaded from http://jana.fzu.cz. Introductory step-by-step tutorials for data processing and structure refinement are available at http://pets.fzu.cz. The source code of Dyngo is available from L.P. upon request.
