# Supplementary material for: Accurate structure models and absolute configuration determination using dynamical effects in continuous-rotation 3D electron diffraction data
Source: Nat Chem. 2023 Apr 20;15(6):848–55. doi: 10.1038/s41557-023-01186-1 (PMC10239730; doi:10.1038/s41557-023-01186-1)
Supplement: Supplementary file 1 — Supplementary text, Tables 1–24 and Figs. 1–19. [file 41557_2023_1186_MOESM1_ESM.pdf]

nature chemistry

Article https://doi.org/10.1038/s41557-023-01186-1

Accurate structure models and absolute configuration determination using dynamical effects in continuous-rotation 3D electron diffraction data

In the format provided by the authors and unedited

Supplementary Materials for

Accurate structure models and absolute configuration determination using dynamical effects in continuous-rotation 3D ED data

Paul B. Klar, Yaşar Krysiak, Hongyi Xu, Gwladys Steciuk, Jung Cho, Xiaodong Zou, Lukas Palatinus

Correspondence to: palat@fzu.cz

This PDF file includes:

Page 2: Further Materials and Methods

Page 4: Supplementary Text

Page 17: Figs. S1 to S19

Page 36: Tables S1 to S24

Page 62: References 1−72

Further Materials and Methods

Diffraction geometry and reflection selection

Diffraction patterns are oriented as seen from the incoming beam, not from behind the detector. The right-handed diffraction geometry is described in such a way that **x** is the tilt axis and **z** points towards the incoming wave vector $\mathbf{K_0}$ of the primary beam. Geometry-based data selection was performed similar to the precession geometry[15]. The excitation error S_g of a reflection with lattice vector **g** is the distance between the point **g** and the Ewald sphere surface at the average goniometer angle α_v.

$$S_{\mathbf{g}} \cong \frac{|\mathbf{K}|^2 - |\mathbf{K}+\mathbf{g}|^2}{2|\mathbf{K}|}$$

The limiting Ewald spheres of a virtual frame correspond to the goniometer angles $\alpha_v \pm \Delta\alpha_v/2$. $S_{\mathbf{g}}^{\max}$ is half the distance between the limiting Ewald spheres through the point **g**. The geometric relationship between a reflection and the limiting Ewald spheres is defined by an absolute parameter D_{Sg} (in Å^{-1}) and a relative parameter R_{Sg}.

$$S_{\mathbf{g}}^{\max} = |\mathbf{g}_{yz}|\varphi_v$$

$$D_{S\mathbf{g}} = S_{\mathbf{g}}^{\max} - |S_{\mathbf{g}}|$$

$$R_{S\mathbf{g}} = \frac{|S_{\mathbf{g}}|}{S_{\mathbf{g}}^{\max}}$$

$\mathbf{g}_{yz} = \mathbf{g} - \mathbf{g}_x$ is the projection of the lattice vector **g** onto the plane perpendicular to the goniometer tilt axis. Reflections with $D_{Sg} < D_{Sg}^{\min}$ and $R_{Sg} > R_{Sg}^{\max}$ were excluded from the refinement (Fig. S4) to avoid partial intensities and the multiple inclusion of reflections **h** that are assigned to more than one virtual frame.

Integrated intensities are calculated for N_{int} orientations for each virtual frame. For each orientation, the structure matrix of the Bloch wave calculation[15] includes reflections with $|\mathbf{g}| < g_{\max}^{BW}$ and $|S_{\mathbf{g}}^{BW}| < 0.01$ Å^{-1}. Note that $S_{\mathbf{g}}^{BW}$ is thus a parameter relevant for each static orientation, whereas $R_{Sg}^{\max}$ and $D_{Sg}^{\min}$ depend on the angular range covered by the virtual frame.

Calculation of *R*-factors and *MR*-factors

For the kinematical and dynamical refinement, the residual factors R_{obs}, R_{all} and wR_{all} were calculated by Jana2006 based on the common definitions:

$$R = \frac{\sum\left|\sqrt{I_{obs}} - \sqrt{I_{calc}}\right|}{\sum\sqrt{I_{obs}}}$$

$$wR = \sqrt{\frac{\sum(w|I_{obs} - I_{calc}|)^2}{\sum(wI_{obs})^2}}$$

$$w = \left(\sigma\left(\sqrt{I_{obs}}\right)^2 + \left(u\sqrt{I_{obs}}\right)^2\right)^{-\frac{1}{2}}$$

The sum runs over all reflections in the case of R_{all} and wR_{all}, and only over observed reflections with $I_{obs} > 3\sigma(I_{obs})$ for the calculation of R_{obs}. The instability factor u was set to 0.01.

In the case of the dynamical refinement, the intensities with symmetry-related indices were not merged. "Merged" *R*-factors *MR* were calculated based on final refinements for comparing dynamical and kinematical refinements. Merged intensities $\overline{I_{\text{obs}}}$ and $\overline{I_{\text{calc}}}$ are determined as the arithmetic mean of symmetry-related reflections, and new uncertainties $\sigma_{\text{M}}(\overline{I_{\text{obs}}})$ are determined using the law of error propagation. New weights w_{M} and *MR*-factors are then calculated analogously to *w*, *R* and *wR*. In the case of the kinematical refinement $MR = R$ and $MwR = wR$.

$$MR = \frac{\sum \left| \sqrt{\overline{I_{\text{obs}}}} - \sqrt{\overline{I_{\text{calc}}}} \right|}{\sum \sqrt{\overline{I_{\text{obs}}}}}$$

$$MwR = \sqrt{\frac{\sum (w|\overline{I_{\text{obs}}} - \overline{I_{\text{calc}}}|)^2}{\sum (w_{\text{M}}\overline{I_{\text{obs}}})^2}}$$

$$w_{\text{M}} = \left(\sigma_{\text{M}} \left(\sqrt{\overline{I_{\text{obs}}}} \right)^2 + \left(u \sqrt{\overline{I_{\text{obs}}}} \right)^2 \right)^{-\frac{1}{2}}$$

Evaluation of the visibility of hydrogen atoms in kinematical and dynamical refinements

Difference electrostatic potential maps were calculated with a resolution of 0.1 Å based on the final structural model from which the hydrogen atoms were removed. A maximum in the potential map was considered to correspond to a hydrogen atom if it was found within 0.4 Å from the refined or constrained hydrogen position and if the difference potential at the maximum was larger than $2\sigma[\Delta V(\mathbf{r})]$.

Supplementary Text

Statistics on structure determinations by 3D ED methods

Although a few dozens of crystals structures determined with electron diffraction methods are deposited into the Cambridge Structural Database and are marked with the keyword "electron diffraction", the database is currently far from complete and does not contain sufficient details on the data acquisition. We therefore selected a non-canonical list of key papers from 2007 to 2018 on which the success of 3D electron diffraction is based [2,15,20,22,23,30,48,50–58], and which are likely to be cited as methodological reference:

- Kolb *et al.* (2007) Towards automated diffraction tomography: Part I - Data acquisition. *Ultramicroscopy* **107**, 507-513
- Kolb *et al*. (2008) Towards automated diffraction tomography. Part II - Cell parameter determination. *Ultramicroscopy* **108**, 763−772
- Mugnaioli *et al*. (2009) “Ab Initio” structure solution from electron diffraction data obtained by a combination of automated diffraction tomography and precession technique. *Ultramicroscopy* **109**, 758−765
- Zhang *et al*. (2010) Collecting 3D electron diffraction data by the rotation method. *Z. Kristallogr*. **225**, 94−102
- Kolb *et al*. (2011) Automated electron diffraction tomography – a new tool for nano crystal structure analysis. *Cryst. Res. Technol*. **46**, 542-554
- Shi *et al.* (2013) Three-dimensional electron crystallography of protein microcrystals. *eLife* 2013;2:e01345
- Nannenga *et al*. (2013) High-resolution structure determination by continuous-rotation data collection in MicroED. *Nat. Methods* **11**, 927−930
- Wan *et al*. (2013) Three-dimensional rotation electron diffraction: software RED for automated data collection and data processing. *J. Appl. Cryst*. 46, 1863-1873
- Yun *et al*. (2014) Phase identification and structure determination from multiphase crystalline powder samples by rotation electron diffraction. *J. Appl. Cryst*. **47**, 2048-2054
- Gemmi *et al*. (2015) Fast electron diffraction tomography. *J. Appl.Crystallogr*. **48**, 718−727
- Palatinus *et al*. (2015) Structure refinement using precession electron diffraction tomography and dynamical diffraction: theory and implementation. *Acta Cryst*. A**71**, 235-244
- Palatinus *et al.* (2015) Structure refinement using precession electron diffraction tomography and dynamical diffraction: tests on experimental data. *Acta Cryst*. B**71**, 740–751
- Cichocka *et al*. (2018) High-throughput continuous rotation electron diffraction data acquisition via software automation. *J. Appl. Cryst*. **51**, 1652-1661
- Gruene *et al*. (2018) Rapid Structure Determination of Microcrystalline Molecular Compounds Using Electron Diffraction. *Angew. Chem. Int. Ed*. **57**, 16313-16317

- Jones *et al*. (2018) The CryoEM Method MicroED as a Powerful Tool for Small Molecule Structure Determination. *ACS Cent. Sci*. **4**, 1587-1592
- Wang *et al*. (2018) On the quality of the continuous rotation electron diffraction data for accurate atomic structure determination of inorganic compounds. *J. Appl. Cryst*. **51**, 1094-1101

We then analysed all peer-reviewed articles published between January 2009 and December 2020 citing one (or more) of these key papers. Reported structure determinations were included in the statistics if one of the following criteria is satisfied:

- a 3D ED data set was recorded AND
- unit cell parameters were determined AND
- the measured crystal was not a protein crystal AND
- a structure was solved or refined against a (combination of) 3D ED data.

Structure determinations passing these criteria were then categorized:

1. Measurement method:
    a. static (ADT, RED, 3D-EDT) OR
    b. precession-assisted (PEDT, ADT with precession) OR
    c. continuous-rotation (MicroED, IEDT, cRED)
2. Highest level of structure determination:
    a. structure solution OR
    b. kinematical refinement OR
    c. dynamical refinement

Studies that used 3D ED only for the determination of unit cell parameters were not counted. If the structure was solved from 3D ED experiments and refined against XRD or ND experiments (e.g. Rietveld refinement), the structure determination was counted as "structure solution". 250 of the analysed papers reported at least one structure determination based on 3D ED experiments. In total 356 structure determinations are included in the statistics (Fig. S1).

Data reduction with PETS2

The software PETS2[3] with graphical user interface is available free of charge for academic use from http://pets.fzu.cz and many introductory examples, including cases from this study, are described in detail in the accompanying tutorial examples. The software was originally written for the data reduction of precession-assisted 3D ED data sets. Recent developments in the program include the support for other diffraction geometries, namely static and continuous-rotation 3D ED, and the generation of overlapping virtual frames.

PETS2 requires a list of frames, the goniometer angles α and β, the calibration constant of a pixel size in reciprocal space in units of $Å^{-1}$ (which is the physical pixel width divided by the product of the detector distance and the electron wavelength λ) and the approximate reflection diameter. Analysis of diffraction

patterns assumes the geometry described in the Methods section, i.e., a TIF file in the standard format shows the diffraction pattern as seen from the diffracting crystal and not as seen from behind the detector. After the automatic peak search, the orientation of the goniometer rotation axis is determined automatically. Identified peaks are then processed for the determination of the unit cell and orientation matrix. Symmetry constraints on the unit cell parameters were used where appropriate. If the unit cell volume deviated by more than about 0.5% from reference unit cell volumes based on XRD measurements at similar temperatures, the calibration constant was adapted accordingly. In the subsequent initial integration step, circular masks with a fixed diameter are placed at expected reflection positions on each frame and integrated intensities are determined by summation of the pixel counts corrected for the background determined from the pixels around the integration mask. The orientation of each frame was optimised by comparing the measured frames with simulated diffraction patterns. The orientation matrix was refined again with the optimised geometry parameters.

Two output files in the well-defined CIF format are generated. The hkl file for kinematical refinement is like the common output of other data reduction programs. For example, frame scale factors and Lorentz correction are applied to the overall integrated intensity of each unique reflection **h**. The hkl file for dynamical refinement includes all relevant geometric parameters describing the diffraction experiment. This includes the orientation matrix, the orientation parameters of the virtual frames and the covered angular range per virtual frame. The second block lists uncorrected, integrated reflection intensities $I_{\mathbf{h}}$ with the corresponding uncertainty $\sigma_{\mathbf{h}}$, reflection indices **h** and the virtual frame number to which this reflection is assigned. The currently implemented algorithm determines $I_{\mathbf{h}}$ as the simple sum of the partial intensities from the frames on which the reflection is expected without any further correction. A Lorentz correction is not needed because of the way the intensities are calculated at the refinement stage. Frame scales are determined at the refinement stage because they cannot be accurately estimated beforehand. Note that one reflection may be assigned to more than one virtual frame and thus may be present in the list more than once.

Parameters of virtual frames

As a final step of the data reduction of static and continuous-rotation 3D ED data with PETS2, the number of experimental frames that form one virtual frame N_{F} and the number of overlapping experimental frames N_{O} that are shared by two subsequent virtual frames define the virtual frames parameters. As described in the main text, parameters must be chosen in such a way that the angular range covered by a virtual frame $\Delta\alpha_{\mathrm{v}}$ is large enough so that the Bloch wave calculations result in complete rocking curves of the assigned reflections. N_{O} should be large enough so that each measured reflection is fully integrated on at least one virtual frame. Using the data sets of α-quartz ($\Delta\alpha = 1.0°$) and α-glycine ($\Delta\alpha = 0.3558°$), we compared different refinements based on different choices of N_{F} and N_{O} (Table S1 and S2). The refinements show little dependence on the parameters of the virtual frames for a broad range of parameters as long as the above considerations are approximately met, i.e., $\Delta\alpha_{\mathrm{v}}$ may not be too small. Consequently, 3D ED experiments with any typically used $\Delta\alpha$ step are suitable for dynamical refinement. Note that the measurement of static diffraction patterns, which is typical for ADT[58] without continuous goniometer rotation, requires a small step size $\Delta\alpha$ to provide sufficiently fine sampling of the reciprocal space. $\Delta\alpha$ should be roughly the same as the crystal mosaicity or, preferably, smaller. Otherwise, dynamical refinement against non-integrated reflection intensities will result in significantly worse figures of merit.

Dynamical refinement with JANA2006

The output file from PETS2 for dynamical refinement with the geometric parameters and reflection list is imported by JANA2006. Where applicable, several data sets were imported individually without merging

symmetrically related reflections to preserve the unique geometric relationship for each measured reflection. For the refinement, intensities of weak and negative observed reflection intensities with $I_{obs} < 0.01\sigma(I_{obs})$ were set to 0 and their uncertainties adapted so that $\sigma\left(\sqrt{I_{obs}}\right) = 5\sqrt{\sigma(I_{obs})}$. Note that this was also applied for the calculation of *MR* and *MwR*-factors. The dynamical refinement, just like the kinematical refinement, requires a starting model. Any representative model from, e.g., other diffraction experiments like X-ray powder diffraction or computational studies like structures optimised by density function theory are suitable starting points. In this study, either the model from the structure solution or from a kinematical refinement was used. If more than one data set is imported, the least-squares refinement uses the reflections from all data sets and benefits from the increased completeness and redundancy. Statistical parameters and descriptors in the well-defined fields in the provided CIF files and in Tables S7, S11, S12, S14, S15, S16, and S17 correspond to this set of reflections. Additionally, JANA2006 provides the *R*-factors and number of observed/all reflections for each subset of reflections originating from one data set. These parameters are provided in the CIF files as part of the field *_refine_special_details*.

Refinement cycles are handled by two programs. Dyngo is a standalone program that calculates dynamic intensities for a set of simultaneously excited reflections determined by the diffraction geometry, determines integrated intensities by numerical integration over a range of crystal orientations and calculates derivatives of dynamical intensities with respect to refinement parameters. Recent developments in Dyngo include the support for non-precession geometries including continuous-rotation 3D ED. JANA2006 handles the least-squares refinement, structural parameters, and geometry parameters. In each refinement cycle and for each virtual frame, JANA2006 determines the set of reflections that are assigned to the respective virtual frame and that pass the geometric filters described in the Methods section. This reflection list together with the relevant geometric parameters are used as input for Dyngo and the calculated intensities I_{calc} are returned together with the respective derivatives needed for the least-squares refinement. The setting of other parameters relevant for the dynamical calculations is discussed and analysed in subsequent sections.

Before the actual refinement, the thickness and frame scales are optimised without refining any structural parameter. Then, these parameters are refined together with the structural parameters for several cycles until convergence is observed. Estimated standard uncertainties and signals in the difference Fourier maps are used to evaluate the completeness and correctness of the refined model. Once the model is complete, the orientation of the virtual frames is optimised. This optimisation is like a refinement cycle during which two orientation parameters per virtual frame are refined. These parameters are not refined together with structural parameters because the number of reflections passing the geometric filters depends on them, leading to instabilities in the refinements. Furthermore, if two orientation parameters per virtual frame were refined, the ratio between observations and parameters would decrease significantly.

In several cases, especially STWP_HPM-1, the refinement was also tested with JANA2020, which is available from the same source as JANA2006 and provided with the same version of Dyngo. The results and refinement output obtained with JANA2020 were identical to those obtained with JANA2006.

Example input file for the Bloch wave program Dyngo

The input file is a fixed-format ACII text file with the file extension .eldyn. Comments are not allowed in the input file, and are included here for explanatory purposes. Comments begin with “%”. Line numbers are given on the left, the longest lines in the input file here extend over two lines.

Format description:

- Line 1: Commands set by Jana2006/2020 describing the task to be performed by Dyngo and, optionally, other settings.
  Main commands (only one allowed):
  - int: calculate intensities and their derivatives
  - scale: optimise frame scale factors
  - orient: find optimal frame orientation
  - thick: find optimal frame thickness
  - thickorient: find optimal frame thickness and frame orientation simulatenously

  Other importand commands (several allowed):
  - iedt: continuous-rotation 3DED
    Without 'iedt', geometry of precession-assisted 3DED is assumed.
  - thr N: number of CPU threads to be used by Dyngo (replace N by e.g. 8)
  - twobeam: apply two-beam formalism in the calculation
  - absorption: include absorption (inelastic scattering) through imaginary part of the electron scattering form factors
  - thickmodel: assume the crystal has a certain shape (thickness distribution)
    available shapes: wedge, cylinder, lens F, ribbon F
    (replace F by flatness parameter between 0.0 and 1.0)
- Line 2: Commands set by user. Same commands as above are available. In case of conflict, the commands of the second line have preference.
- Line 3: Frame number
- Line 4: Centro- or non-centroymmetric structure, number of structure parameters (Npar)
- Line 5: Refinement type F/F2. Refinement against diffracted amplitudes (F) or intensities (F2).
- Line 6−8: Orientation matrix
- Line 9: wavelength; F_0; (unused) placeholder; $g_{\max}^{\mathrm{BW}}$; $S_{\mathbf{g}}^{\mathrm{BW}}$; $S_{\mathbf{g}}^{\max}$; $R_{Sg}^{\max}$; N_{int};
- Line 10: frame orientation parameters. U, V, W, α_{V}, β_{V}, ½$\Delta\alpha_{\mathrm{V}}$
  Dyngo assumes that α_{V} and β_{V} describe the average frame orientation, and that the goniometer moved from $\alpha_{\mathrm{V}}-\frac{1}{2}\Delta\alpha_{\mathrm{V}}$ to $\alpha_{\mathrm{V}}+\frac{1}{2}\Delta\alpha_{\mathrm{V}}$. The "zone axis" UVW is not used by Dyngo.
- Line 11: frame scale; thickness; 2x (unused) placeholder; refinement keys
- Line 12: orientation correction angles[49] EDphi and EDtheta (and refinement keys)
- Line 13: Reflection list and parameters. h; k; l; I_{obs}; $\sigma(I_{\mathrm{obs}})$; A=Re(F_{calc}); B=Im(F_{calc}); usage key; $S_{\mathbf{g}}$
  The usage key defines if a reflection is used in the least-squares refinement or not.

Key	Reflections filters passed	Reflection assigned to frame	Intensity calculation requested	Use in least-squares refinement
1	FALSE	TRUE	FALSE	FALSE
2	TRUE	FALSE	FALSE	FALSE
5	TRUE	TRUE	TRUE	TRUE
6	FALSE	FALSE	FALSE	FALSE

- Line 14−15: dA/dp, partial derivatives of real part of structure factor with respect to structure parameters. There are Npar entries with a maximum of 10 entries per line.
- Line 16−17: dB/dp, partial derivatives of imaginary part of structure factor
- Lines 18−22 and 23−27 are like lines 13−17, but for other reflections. The example input file only contains 3 reflection blocks, but typically there are many more reflections.

```
int iedt thr 8                        % Commands set by Jana2006/2020
                                      % Commands set by user
Zone#  1                              % Frame number
Noncentrosymmetric        14          % centro-/noncentrosymmetric, Npar structure parameters
Refinement F                          % refinement against diffracted amplitudes or intensity
   -0.070180   -0.021251    0.176241  % orientation matrix (3 lines)
    0.221929    0.141614    0.053071
   -0.029104    0.185792   -0.020293
    0.025100   29.382000    0.000000    2.000000    0.010000    0.100000    0.850000   42
-1.000000 0.075640-0.284040-49.19700-0.130000 1.000000                      % orientation
 732.2343 437.8342 0.000000 0.000000                     1100       % thickness, scale
-98.37066 0.344869                                       00         % orientation correction
  -1  -2   3    0.53414E+02    0.16051E+01    0.22373E+01   -0.24447E+01    1   -0.12062E-01
   0.581825E+01  -0.111123E+01  -0.434239E+01  -0.701179E+01  -0.210361E+02   0.103876E+01
  -0.128771E+02   0.185571E+02  -0.198075E+01  -0.898722E+01
  -0.131236E+02   0.152137E+01  -0.165449E+02   0.123859E+02   % Npar derivatives dA/dp
   0.197711E+02  -0.931391E+00   0.545805E+01   0.582000E+01  -0.787313E+01  -0.203842E+02
   0.186951E+02   0.130983E+02   0.961098E+01   0.437499E+01
   0.161821E+02  -0.279971E+01   0.736595E+01   0.266540E+01   % Npar derivatives dB/dp
   1   2  -3    0.11716E+03    0.17151E+01    0.22373E+01    0.24447E+01    5   -0.53496E-02
   0.581825E+01  -0.111123E+01  -0.434239E+01  -0.701179E+01  -0.210361E+02   0.103876E+01
  -0.128771E+02   0.185571E+02  -0.198075E+01  -0.898722E+01
  -0.131236E+02   0.152137E+01  -0.165449E+02   0.123859E+02
  -0.197711E+02   0.931391E+00  -0.545805E+01  -0.582000E+01   0.787313E+01   0.203842E+02
  -0.186951E+02  -0.130983E+02  -0.961098E+01  -0.437499E+01
  -0.161821E+02   0.279971E+01  -0.736595E+01  -0.266540E+01
   1 -16  -8    0.00000E+00    0.00000E+00    0.42181E-01    0.13972E-04    6    0.00000E+00
  -0.138935E+01  -0.360471E+01  -0.298443E+01  -0.174981E+01   0.197432E+01  -0.612585E+00
   0.723789E+00  -0.421756E+00  -0.360771E+01  -0.281865E+01
  -0.949799E+00   0.569956E+01  -0.211119E+01   0.284715E+01
   0.627227E+00  -0.428719E+01   0.160581E+01  -0.712069E+00   0.480263E+01  -0.339341E+00
   0.180621E-01   0.157926E+00   0.274796E+01   0.120872E+01
   0.711175E+00  -0.255732E+01   0.823157E-02  -0.128506E+01
%  ... list of reflections and derivatives continues
```

Number of integration steps for dynamical calculations

The number of integration steps N_{int} is an important parameter that must be chosen before the dynamical refinement. It determines the number of orientations for which the Bloch wave calculations are performed for each virtual frame. It is thus related to the sampling of the calculated rocking curves which are subsequently integrated yielding the integrated calculated intensities of the contributing reflections. N_{int} should be high enough to ensure a good sampling, but also as low as possible to reduce the computational cost. Figure S5 shows the dependence of wR_{all} on $N_{int}/\Delta\alpha_v$ (continous-rotation 3DED) and $N_{int}/(2\varphi)$ (precession-assisted 3DED). φ is half the opening angle of the precession cone for precession-assisted 3D ED and has a similar geometric meaning as $\Delta\alpha_v/2$ in the case of continuous-rotation 3D ED. ΔwR_{all} is calculated relative to wR_{all} of the refinement with $N_{int} \rightarrow \infty$. The first observation is that a too small N_{int} has a strong impact on wR_{all}. A coarse sampling of the rocking curve obviously results in worse *R*-factors. The second observation is that convergence is observed for different data sets at different N_{int} normalized by the covered angular range. This depends on the refined thickness, which for the continuous-rotation data sets is 116(9) nm, 44(3) nm and 36(3) nm for abiraterone acetate, α-quartz and natrolite, respectively. A larger refined thickness thus requires more integration steps, which is expected because the increased thickness decreases the rocking curve width and thus requires a finer sampling. The refined thickness of 48(3) nm against the data set of quartz recorded with beam precession ($\varphi = 0.92°$) is in good agreement with the non-precession data ($\Delta\alpha_v/2 = 1.0°$), but convergence requires a much larger N_{int}. This is easily explained by the corresponding set of different orientations adopted by the crystal relative to the primary beam. The expected ratio of N_{int} with and without beam precession is $2\pi\varphi/\Delta\alpha_v$. This ratio simplifies to the constant π if $\varphi = \Delta\alpha_v/2$, which is in good agreement with the observation that the dynamical refinement of quartz against precession-assisted data requires ~3 times more integration steps than the refinement against continuous-rotation data.

For dynamical refinements against static and continuous-rotation 3D ED data, N_{int} may be initially set to 25 steps/°. If the refined thickness is significantly larger than 40 nm, the initial N_{int} should be set to about 30 to 40 steps/°. In the final refinement cycles, N_{int} was increased to at least 50 steps/° as a quality check. If the thickness increased upon increasing N_{int}, the number of integration steps was further increased until convergence was observed.

Resolution limit of dynamical calculations

Another parameter that has a strong impact on the computation times is the resolution limit g_{max}^{BW} which is related to the number of reflections contributing to the dynamical calculations. Obviously, g_{max}^{BW} must be equal or greater than g_{max}^{ref}, which is the resolution limit of reflections used in the refinement, because otherwise I_{calc} is not determined for all reflections in the least-squares refinement. g_{max}^{BW} should be equal to the highest resolution reflection which was excited during the experiment. In most cases, it is sufficient to set $g_{max}^{BW} = g_{max}^{ref}$. If g_{max}^{ref} is lower than the theoretically obtainable resolution of the data set, g_{max}^{BW} should be increased (Fig. S6). The latter is expected for example for data sets with a large detector distance so that high-resolution reflections are not measured by the area detector. However, a smaller g_{max}^{BW} and g_{max}^{ref} may drastically decrease the computational cost and duration of a dynamical refinement cycle. In the more time-consuming refinements for this study (limaspermidine, abiraterone acetate, CAU-36 and especially MBBF4), initially g_{max}^{BW} was set to g_{max}^{ref}. In the cases with smaller unit cells, g_{max}^{BW} was typically set to $g_{max}^{ref} + 0.1$ Å^{-1}. g_{max}^{BW} was further increased for the final refinement cycles to check that convergence was achieved. An exception was STW_HPM-1, for which g_{max}^{BW} larger than 1.35 Å^{-1} could not be used due to the technical limitations of Jana2006 (total allocated memory exceeded available memory). In this case, therefore, g_{max}^{BW} and g_{max}^{ref} were set to 1.35 Å^{-1}.

Comparison between PETS/Jana2006 and XDS/SHELXL

One of the most popular programs for the data reduction of continuous-rotation 3D ED data is XDS[59] (X-ray Detector Software). The most-used refinement software for small-molecule crystal structures is SHELXL[1]. All results presented in the main article are based on data reductions with the software PETS2 (Process Electron diffraction Tilt Series) and refinement with Jana2006. The only exception is the kinematical refinement of albite, for which the published data reduction based on XDS was used for comparison. Different structure determinations were compared for benchmark purposes (Table S3). Previously published kinematical refinements by the respective authors who provided the data sets are also included in Table S3 for the sake of completeness. Although the results for individual data sets differ slightly between the software packages, the comparison shows that the use of PETS2 and Jana2006 does not produce systematically worse results than the use of alternative software.

Comparison of 3D ED measurements with and without precession

Precession-assisted 3D ED is a very successful and powerful method, which is, however, based on the availability of a suitable hardware extension of the TEM for the accurate control of the beam precession. Experimental integrated intensities I_{obs} are obtained from a single frame. On the basis of these independent frames, dynamical integrated intensities I_{calc} can be calculated and compared with I_{obs}. This direct correspondence between experimental frames and dynamical calculations facilitated the development of software packages that take dynamical diffraction theory into account. In the last years, especially the combination of Dyngo with Jana2006 proved to be a versatile tool for accurate structure determination from precession-assisted 3D ED experiments[14,15,35,48].

There are several advantages of continuous-rotation 3D ED over precession-assisted 3D ED which become apparent by comparing the reciprocal space volumes $V^*_{precession}$ and $V^*_{continuous}$ sampled by a single

frame with precession and with continous-rotation, respectively (Fig. S7). Within the small-angle approximation, the ratio $V^*_{precession}/V^*_{continuous}$ is $\pi/2$ if $\varphi = \Delta\alpha/2$. Consequently, with beam precession a longer exposure time is needed to achieve the same integrated intensity as without precession. Although the larger volume also improves the completeness as more unique reflections are measured, the overall effect on the completeness of a measured data set is negligible. More importantly, the intensity information of a reflection **h** from two neighbouring frames cannot be combined, because their rocking curves do not have a simple relationship if precession is used. Finally, all reflections for which the exact Bragg condition is not fulfilled during one precession circle are incompletely integrated and must be excluded from the refinement.

These disadvantages do not hold if a static or continuous-rotation geometry is used. Every (partial) reflection on each frame (except for the very first and last frames) contributes to the final integrated intensity, which strongly improves the signal-to-noise ratio and further reduces the necessary electron dose per frame. This is a big advantage of continuous-rotation 3D ED because more appreciable reflection intensities are obtained from a single beam-sensitive crystallite with a weaker electron beam.

As an experimental assessment of the dynamical refinement against continuous-rotation 3D ED data, a measurement protocol was designed to record precession-assisted 3D ED and continuous-rotation 3D ED data sets in a quasi-simultaneous way. This protocol was used to determine the structures of α-quartz and natrolite with the aim to compare the respective dynamical refinements. Precession-assisted and continuous-rotation 3D ED measurements of quartz used the identical exposure time, but in the case of natrolite the exposure time of diffraction patterns recorded with beam precession was increased by 20%.

Very similar *R*-factors were achieved by dynamical refinements, at the level of ~0.06 for quartz and ~0.08 for natrolite (Tables S4 and S5). The coordinates from the new dynamical refinement against the continuous-rotation 3D ED data sets were visibly closer to X-ray-based reference coordinates than the refinement against precession-assisted 3D ED (Tables S6 and S7). Although with beam precession more observed reflections were used in the refinements, the geometric aspects described above apparently have a larger impact on the accuracy of the refinement.

Frame-based kinematical refinement

The dynamical refinement requires a different data processing than the kinematical refinement, and the refinement itself is also quite different. The two main differences are that in the dynamical refinement the intensities are not symmetry-averaged, and that scale factors of individual OVFs are refined. It is thus legitimate to ask, if the observed improvement from kinematical to dynamical refinement can indeed be attributed to the description of the dynamical effects, or if it is due to the different data processing and refinement strategy.

To answer this question, we have introduced a procedure that we name frame-based kinematical refinement. This refinement is in all aspect equivalent to the dynamical refinement, i.e., it uses the same data processing, same refinement strategy, same number of parameters. The only difference is that the site occupancy factors of all refined atoms are reduced to a small fraction of their full occupancy. In the kinematical limit, such operation has no effect, as it is perfectly correlated with the scale factor(s). In the dynamical refinement, this is, however, not the case. The dynamical effects depend on the thickness and on the strength of the interaction between electrons and the crystal. The reduction of the site occupancy factors within the framework of the Bloch wave formalism is equivalent to the reduction of the interaction strength, while preserving all other parameters the same, thus making the intensities less affected by dynamical effects. At the limit of very low occupancies, the diffraction becomes kinematical.

We have performed detailed calculations on α-quartz, natrolite, abiraterone acetate, and limaspermidine. In both cases, we performed a series of refinements with site occupancy factors between 1.0, corresponding to the normal dynamical refinement, and 0.01, which corresponds to hundred times reduced strength of the dynamical scattering yielding intensities very close to the kinematical limit. *R*-factors are plotted as a function of the site occupancy factor in Fig. S8. Table S5 then contains the *R*-values of the dynamical, frame-based kinematical and standard kinematical refinements for more compounds. The table shows that the MwR_{all} values of the frame-based kinematical refinement are similar or slightly higher than the wR_{all} values of the standard kinematical refinement, illustrating clearly that the improvement in the fit between dynamical and kinematical refinement can indeed be attributed to the more correct description of the dynamical effects.

Fig. S8 also contains the wR_{all} values of equivalent refinements against the inverted structures. It can be seen that the difference in *R*-factors reduces as the intensity of the dynamical effects decreases, until they become almost identical in the refinements with site occupancy factor 0.01. This further illustrates the fact that the differences between the refinement of the enantiomorphs can be entirely attributed to the enantiomorph-sensitive dynamical effects. These differences vanish in the kinematical limit.

Al/Si distribution in albite

From the 16 available data sets of albite[19], 3 data sets (author labels 000, 002 and 014) were selected for the dynamical refinement based on resolution-dependent reflection statistics with an overall completeness of 66%. Kinematical refinements using only these 3 data sets were not as conclusive as the published results based on 16 data sets from 9 different crystals[19]. Therefore, the kinematical refinement of albite with JANA2006 presented in this study was based on the published hkl file generated by data processing with XDS. All 16 possible Al/Si distributions were refined applying isotropic displacement parameters without specific restraints or constraints. Both the dynamical and the kinematical refinements identified the correct Al/Si distribution, which is also confirmed by the cation-oxygen distances as the refinement with the best *R*-factors. The absolute differences between the *R*-factors of models with different Al/Si distributions were only marginally larger in the kinematical than in the dynamical refinement, but as the dynamical *R*-factors are roughly by a factor of 2.3 smaller, the relative difference between the *R*-factors is more significant in the dynamical refinement (Fig. S10). Quantitatively, the second-best *R*-factor is 0.8% higher than the best for dynamical refinement, and 0.5% higher for the kinematical, for the third best the increase is already 2.7% for dynamical vs 1.0% for kinematical refinement. The worst *R*-factor is 9.5% larger in the dynamical refinement and only 3.5% in the kinematical refinement. Another clear indicator is the range of the displacement parameters of the Al and Si atoms (Fig. S11), which is much narrower for the correct atom type assignment than for all incorrect ones in the dynamical refinement. This difference, although still present, is weaker in the kinematical result. This analysis together with the lower *R*-factors in general significantly increases the confidence with which the dynamical refinement identified the Al and Si sites.

Absence and presence of guest molecules in mordenite and CAU-36

CAU-36 contains 1,4-diazabicyclo[2.2.2]octane, abbreviated as DABCO (Schoenflies symbol C_{3h}), as guest molecule. Both the dynamical and the kinematical refinement reveal all 8 atom sites in the DESP of a refinement without the guest molecule. However, additional maxima in the subsequent DESP of the dynamical refinement including the guest molecule suggest that the molecule position is disordered with two orientations, the second of which is related to the first by a 60° rotation around the 3-fold rotation axis of the DABCO molecule. This signal, while weak and noisy, is clearly visible in the DESP maps (Fig. S9). In the equivalent DESP of the kinematical refinement the second orientation could not be clearly identified (Fig. S9A,B). The second orientation was added with constrained geometry and fixed

orientation relative to the initially identified guest molecule. With the dynamical approach the occupancy of the second orientation refines to 0.064(4), in the kinematical refinement to 0.074(8).

The mordenite crystal (diameter ~250 nm) was measured after calcination with continuous-rotation 3D ED at room temperature[20]. The DESP maps of the dynamical and kinematical refinement were analysed to evaluate the presence of water molecules, which are expected to be absent due to the calcination step. The standard uncertainty of the difference electrostatic potential $\sigma[\Delta V(\mathbf{r})]$ is significantly lower in the case of the dynamical refinement (Tab. S9). Assuming a thermal displacement parameter of 0.15 Å², a disordered oxygen site with an occupancy of 10% is expected to be visible in the DESP map above the 3σ-level. In the DESP from the kinematical refinement, such an oxygen atom cannot be distinguished from spurious noise peaks, because the noise level is higher by a factor of 3.2. Hence, even 25% occupied oxygen atoms could well be hidden in noise (Fig. S9C). Only the dynamical refinement thus suggests that the calcination indeed removed any water molecules from the pores, which was confirmed by infrared spectroscopy.

Static 3D ED and beam-induced disorder in CAP

Data acquisition in continuous-rotation mode in general yields a better sampling of the rocking curve in comparison to static 3D ED. However, the presented approach is in general applicable to both and the results on the dynamical refinement of the structure model of CAP are considered a proof of principle. The two crystals of CAP were measured by sampling reciprocal space in 0.1°-steps. During the exposure, the beam and the sample stage (goniometer) are static, like in the first 3D ED data acquisition protocols like ADT[50,58] (Automated Diffraction Tomography) and RED[51] (Rotation Electron Diffraction). Data reduction of data sets with static diffraction patterns follows the same procedure like the data reduction of continuous-rotation 3D ED data. The only difference is that the input files for PETS2 use the keyword "geometry static" instead of "geometry continuous". The generation of virtual frames and the subsequent dynamical refinement are identical for both geometries.

The static 3D ED setup as implemented for the measurement of CAP has the drawback that the measurements take significantly longer (in this case, about 1 hour for an angular range of 100°). As crystals are constantly exposed by the electron beam, the potential for beam damage is significantly increased and in the case of CAP induces an occupancy disorder on the cobalt sites and related displacement disorder, which is discussed in more detail here. In the hypothetical ordered CAP structure with chemical composition $CoAl_2P_4O_{20}H_{12}$ and only fully occupied sites, the oxygens of the CoO_6 octahedron are part of two water molecules and two hydroxyl groups. The measured sample contains more Co so that two Co sites are partially occupied. XRD-based refinements indicate that the occupancy of Co1 is much larger than the occupancy of Co2, which is also confirmed by precession-assisted 3D ED experiments with optimized electron dose[35]. An increased exposure of CAP crystals to an electron beam triggers several correlated structural changes:

- Co atoms migrate from Co1 to Co2 site
- Hydroxyl groups migrate from O1 to O6 site
- Water molecules of O2 site adapt orientation depending on local occupancy of Co1 and Co2
- PO_4 tetrahedra and AlO_6 octahedra tilt by a few degrees

Consequently, the coordinates of almost all atoms change as a function of the occupancy of Co1 and Co2, but the change is most pronounced for oxygens coordinating the P atoms. For example, a linear

dependence of the z coordinate of O2 on the reduced occupancy difference $\frac{o_1 - o_2}{o_1 + o_2 - o_1 o_2}$ has been observed, where o_1 and o_2 are the occupancies of Co1 and Co2, respectively. This relationship is further confirmed by the presented dynamical refinement and fits better to the trend than the kinematical refinement (Fig. S16A).

The occupancy of the H atom of the hydroxyl group involving the O6 site, which is only occupied if Co1 is not occupied, is expected to be $1-o_1 = 0.28$. The large displacement parameter of O6 and the absence of potential hydrogen bond acceptors suggests that the orientation of the hydroxyl group may be not well defined, and no peak in the vicinity of O6 was identified in the difference potential map (Fig. S16B). The water molecule at the O2 site is expected to be present in three different orientations, corresponding to the three possible environments: a) only Co1 occupied, b) only Co2 occupied and c) both Co sites simultaneously occupied. Consequently, there may be up to 6 different hydrogen sites to describe the three corresponding water molecule orientations. However, the refinement revealed that the H3 site is part of a hydrogen bond with a donor-acceptor distance (O2−O4) of 2.77 Å (O2-H3-O4 angle is 160.4°). As no other potential hydrogen bonds involving O2 could be identified, it is reasonable to assume that there are in total 4 hydrogen sites in the vicinity of O2, of which three try to maximise the distance to the occupied Co site(s).

These considerations are in very good agreement with the observation in Fig. 4D: In the difference Fourier map, the potential at the H3 site is visibly stronger than that of the other hydrogen site (H4), suggesting that the latter is not fully occupied. Assuming that the identified H4 site corresponds to case a), two additional sites H4' and H4" are expected to be present corresponding to the respective environments of cases b) and c). The expected occupancies are $1-o_1=0.28$ and $o_1+o_2-1=0.14$, respectively. However, only one additional site close to O2 could be identified above $2\sigma[\Delta V(\mathbf{r})]$, which most likely corresponds to H4' with an occupancy of 0.28 (black arrows in Fig. S16). The (restrained) dynamical refinement including the H4' site improved the wR_{all} from 0.1185 to 0.1177, which is negligible and was therefore not used in the final refinement presented in the main article.

Survey on C-H distances in 3D ED-based structure determinations

Using the program ConQuest of the Cambridge Structural Data Center, the Cambridge Structural Database CSD[60] was searched for all close distances between C and H atoms limited to structures that were labelled with the keyword "electron diffraction". The database is far from complete and there are many more published structures (Fig. S1) than deposited structures. However, this quick survey clearly shows that in the 50 deposited structures fulfilling the search criteria there are two dominating distances (Fig. S11): The first around 0.95 Å corresponding to the default distance constraints used in many XRD refinement software packages for different temperatures, orbital hybridisation and chemical environments. The other region is just below 1.1 Å corresponding to the default C-H distances derived from ND-based structure determinations[26].

Computational cost for dynamical refinement cycles

The computational cost for dynamical refinements is much higher than for kinematical refinements. It is approximately proportional to the number of parameters and the cube of the primitive unit cell volume V_{P}. An overview of representative refinement cycle times is given in Table S5.

Absolute structure determination and assumed diffraction pattern orientation

For non-centrosymmetric structures, the absolute structure is determined initially by repeating the dynamical refinement starting with the inverted model and comparing the refinement figures of merit of the two final models as described in the Methods section.

As pointed out in several sections before, PETS assumes that the TIF files show diffraction patterns as seen from the crystal. If this convention is not followed or raw diffraction patterns are flipped, the signs of *hkl* indices are not correct and the absolute structure determination will identify the wrong enantiomorph. A rotation of diffraction patterns during any processing and data conversion step has no effect on the final structure determination.

A pattern flip or rotation can only be detected if the goniometer rotation direction and calibrated orientation of the rotation axis relative to the detector axes are known. A careful check at the data reduction stage then reveals an unexpected orientation of the goniometer rotation axis.

For the preparation of this manuscript, the initial absolute structure determination by dynamical refinement of α,β-dehydrocurvularin and epicorazine A was opposite to the expected one. The data sets of these two compounds stem from the same study and microscope[44]. A careful analysis of all file processing steps and a helpful discussion with the involved authors led to the finding, that the raw diffraction patterns written by the Timepix hybrid pixel detector (Amsterdam Scientific Instruments) by default were written not as seen from the crystal, but as seen from behind the detector. As the TEM setup with the used detector was only temporary, the "flip" was not noticed before our reinvestigation of the data.

Absolute structure determination of quartz, natrolite and CAU-36

The handedness of quartz was determined to correspond to right (dextro) quartz. The refinement of the other enantiomorph (left quartz) increased the wR_{all} from 0.186 to 0.192 based on the structure solution or from 0.084 to 0.104 for the refinement of the final model before optimisation of the orientation of the virtual frames. The z-score of the absolute structure assignment is 6.9σ. Absolute structure determination of the polar structure of natrolite identifies the absolute orientation of the crystal. With a striking drop of the wR_{all} from 0.217 to 0.174 the correct orientation was easily identified based on the model of the structure solution. The gap increased upon refinement of the coordinates and displacement parameters with respective wR_{all} of 0.172 and 0.104. The z-score of the assignment of the orientation of the measured natrolite crystal is 17.7σ. A similar approach did not reveal a striking difference in R-factors of refinements against individual data sets of CAU-36, although its space group P-4c2 is non-centrosymmetric. However, R-factors improved by refining the structure model as inversion twin using a method described in[61]. Refined twin fractions for the different data sets are between 0.4 and 0.6. Currently, the optimisation of virtual frame orientations of twinned structures is not supported within Dyngo and Jana2006.

Meaning of symbols and parameters used in supplementary text, tables and figures

λ: electron wavelength

T: sample temperature during the measurement

α_{min}, α_{max}: minimum and maximum goniometer angle of the measurement

$\Delta\alpha$: difference in goniometer angle between two subsequent experimental frames

#1, #2, … #8: data set identifier

η_{exp}: angle by which the sample is rotated during the exposure of the diffraction pattern

η_{gap}: angle by which the sample is rotated during detector readout and/or preparation of next frame

Z: number of formula units in the unit cell

mosaicity: apparent mosaicity as determined during the data reduction with PETS2

N_F: number of experimental frames composing one virtual frame

N_O: number of experimental frames that are used by two subsequent virtual frames

N_{OVF}: number of virtual frames used in the dynamical refinement

$\Delta\alpha_v$: angular range covered by a virtual frame

$\Delta\alpha_o$: overlapping angular range between two subsequent virtual frames

R_{Sg}^{max}: maximum R_{Sg} of reflections used in the refinement

D_{Sg}^{min}: minimum D_{Sg} of reflections used in the refinement

g_{max}^{BW}: maximum resolution of beams used in the Bloch wave calculation

$g_{max}^{ref} = 2\sin\theta_{max}/\lambda$: resolution limit of reflections used in the refinement (expressed in reciprocal space)

d_{min}: resolution limit of reflections used in the refinement (expressed in real space)

N_{obs}: number of reflections with $I_{obs} > 3\sigma(I_{obs})$

N_{all}: total number of reflections used in the refinement

R_{obs}: conventional R-factor ($R1$) based on N_{obs} observed reflections

wR_{all}: weighted R-factor based on all reflections

$\sigma[\Delta V(\mathbf{r})]$: standard uncertainty of the difference electrostatic potential $\Delta V(\mathbf{r})$ in e/Å

min[$\Delta V(\mathbf{r})$], max[$\Delta V(\mathbf{r})$]: minimum and maximum value $\Delta V(\mathbf{r})$ in e/Å

φ: precession angle (semi-angle of the opening cone)

Supplementary Figures

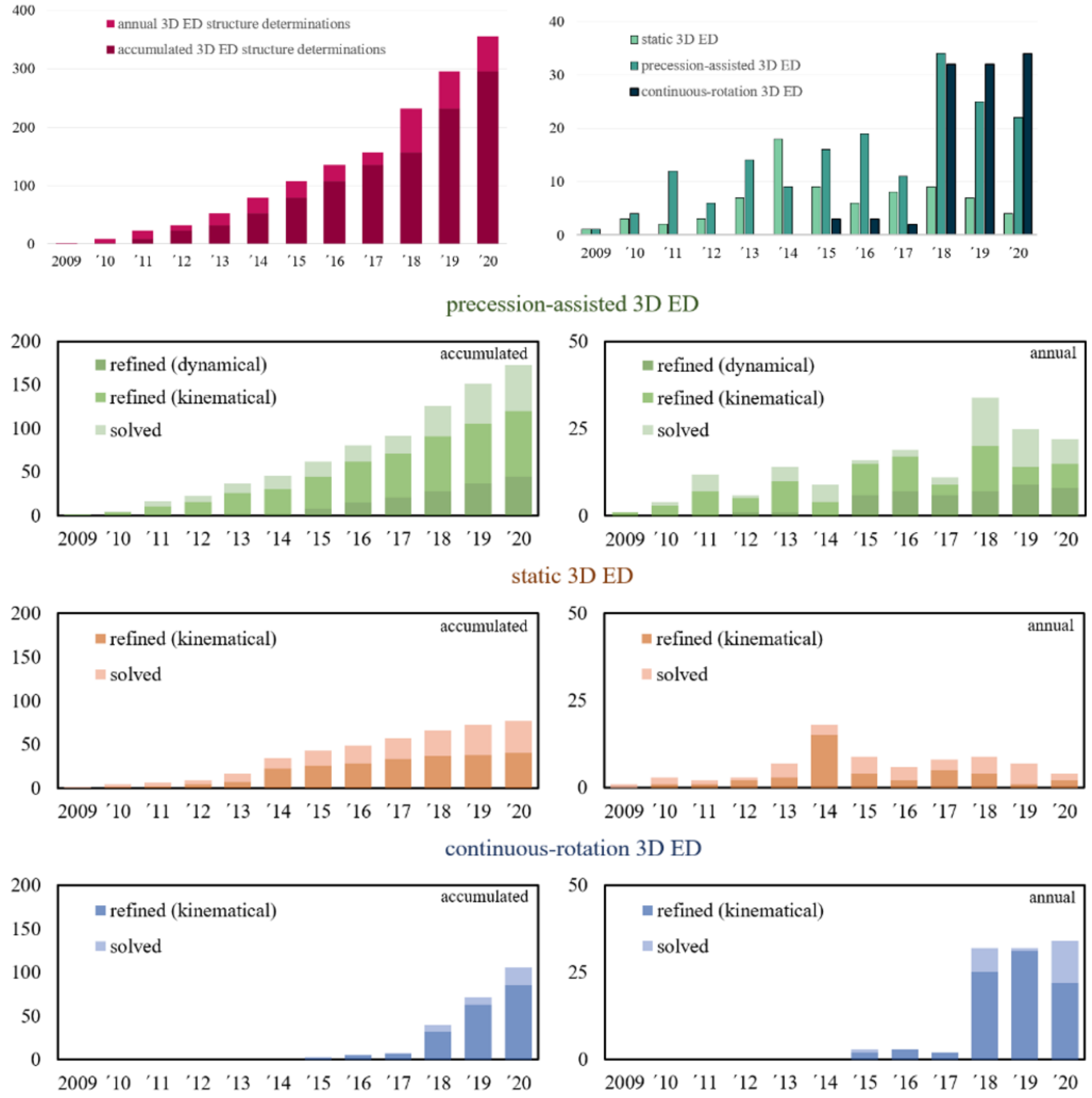

Fig. S1. Structure determination statistics.
Detailed statistics from the non-automated literature survey on the 3D ED data acquisition methods used for the different levels of structure determinations.

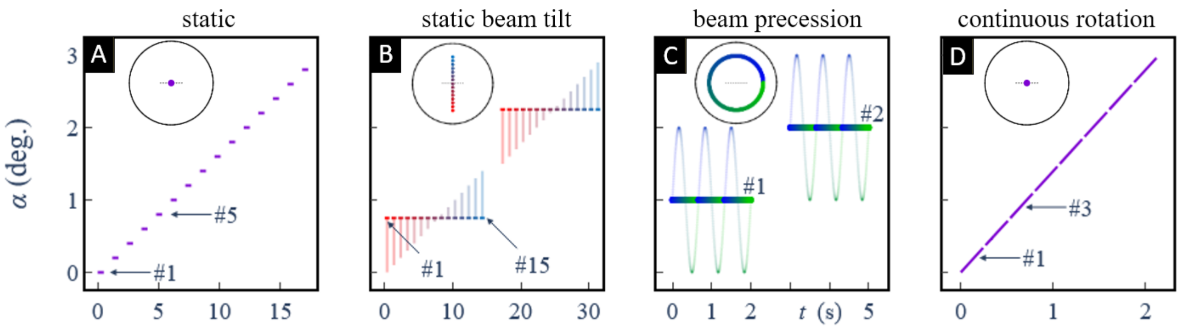

Fig. S2. 3D ED methods.

Goniometer and beam control as a function of typical time elapsed during 3D ED data acquisition covering a goniometer rotation of about 3°. Solid lines indicate the goniometer position during the exposure of a frame. The effective α angle due to a tilted beam is plotted with decreased opacity. Insets illustrate the beam tilt as pole figures (horizontal line is parallel to goniometer axis) with the outer circle representing an angle of about 1.5°. The beam precession in (**C**) is typically performed 100 times per second. Arrows point to individual frames with the corresponding frame number.

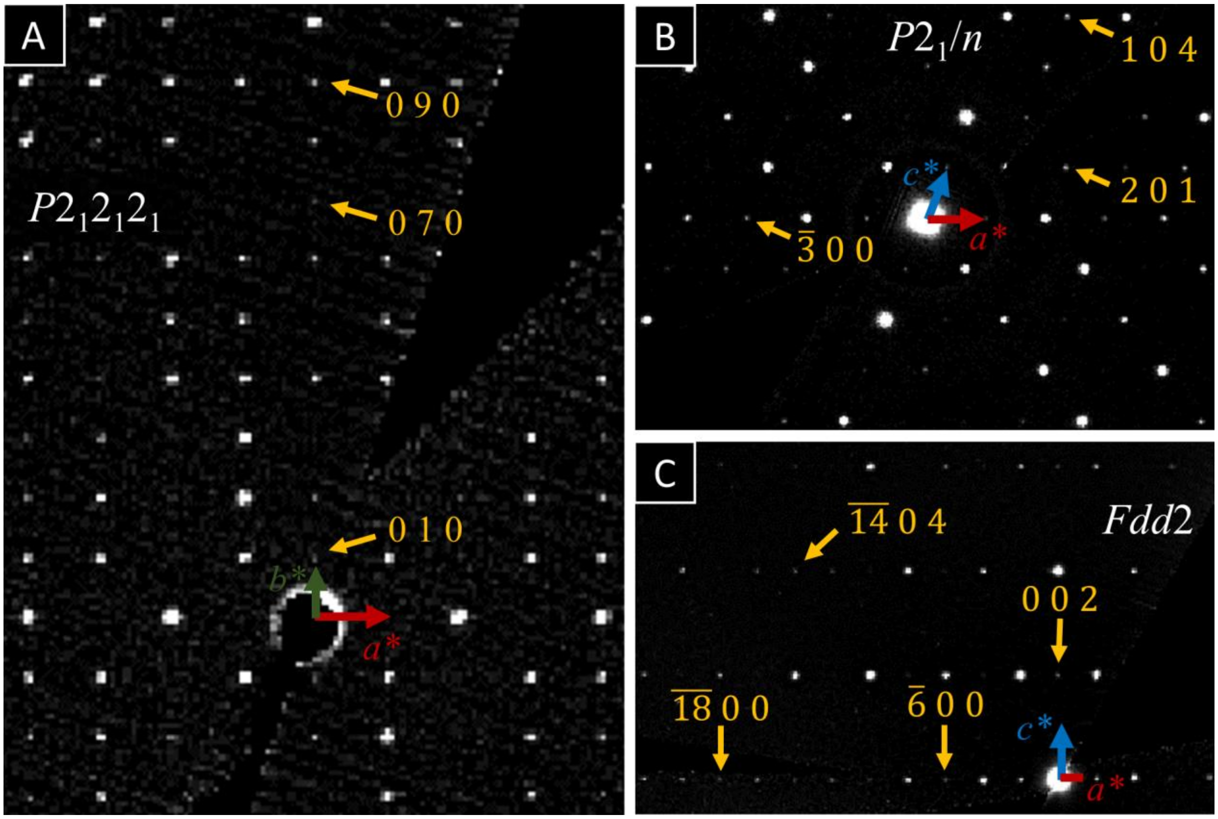

Fig. S3. Reciprocal space sections with violations of reflection conditions.
Selected reciprocal space sections reconstructed with PETS2. Selected reflections violating the reflection conditions of the space group are marked with an arrow. *a** direction is parallel to the horizontal direction pointing right. (**A**) progesterone, data set #4 (352382), space group $P2_12_12_1$, *hk*0 section, with violations of the reflection condition 0*k*0: *k*=2*n*. (**B**) α-glycine, space group $P2_1/n$, *h*0*l* section, with violations of the reflection condition *h*0*l*: *h*+*l*=2*n*. (**C**) natrolite, space group *Fdd*2, *h*0*l* section, with violations of the reflection condition *h*0*l*: *h*+*l*=4*n*.

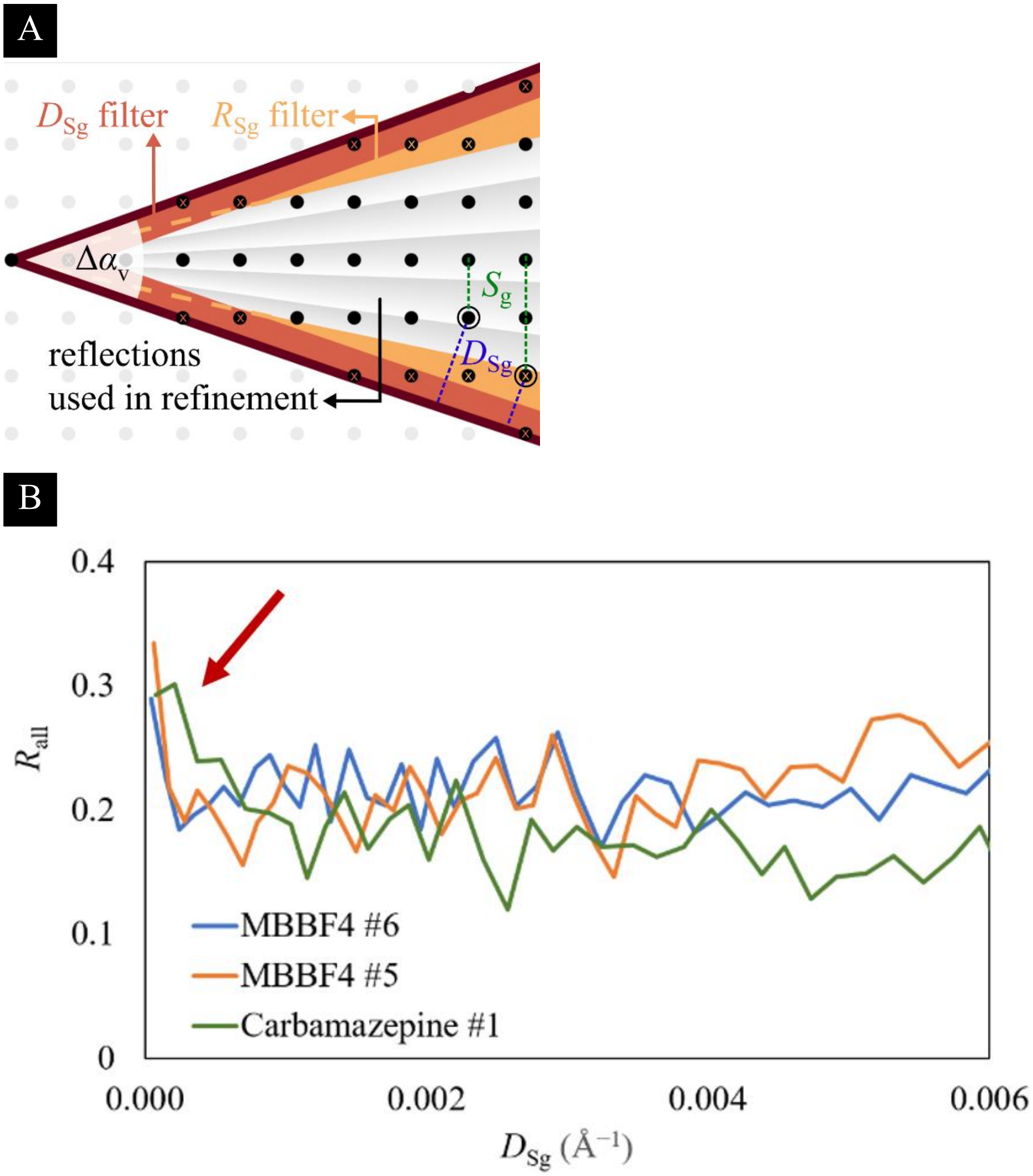

Fig. S4. Reflection selection filters.
(**A**) Effect of D_{Sg} (red areas) and R_{Sg} (orange areas) filters on selection of reflections (black disks) assigned to a virtual frame that are used in the dynamical refinement. Reflections that are filtered out are marked with an ×. Excitation error S_g and related D_{Sg} are drawn for two selected reflections (encircled). (**B**) R_{all} of subsets of 100 reflections sorted by D_{Sg} of 3 representative data sets for which the D_{Sg} filter was not used. The shells with lowest D_{Sg} (marked with an arrow) have a visibly increased R_{all} because only partial intensities are calculated for those reflections. The D_{Sg} filter removes reflections in the lowest shell according to a user-defined threshold.

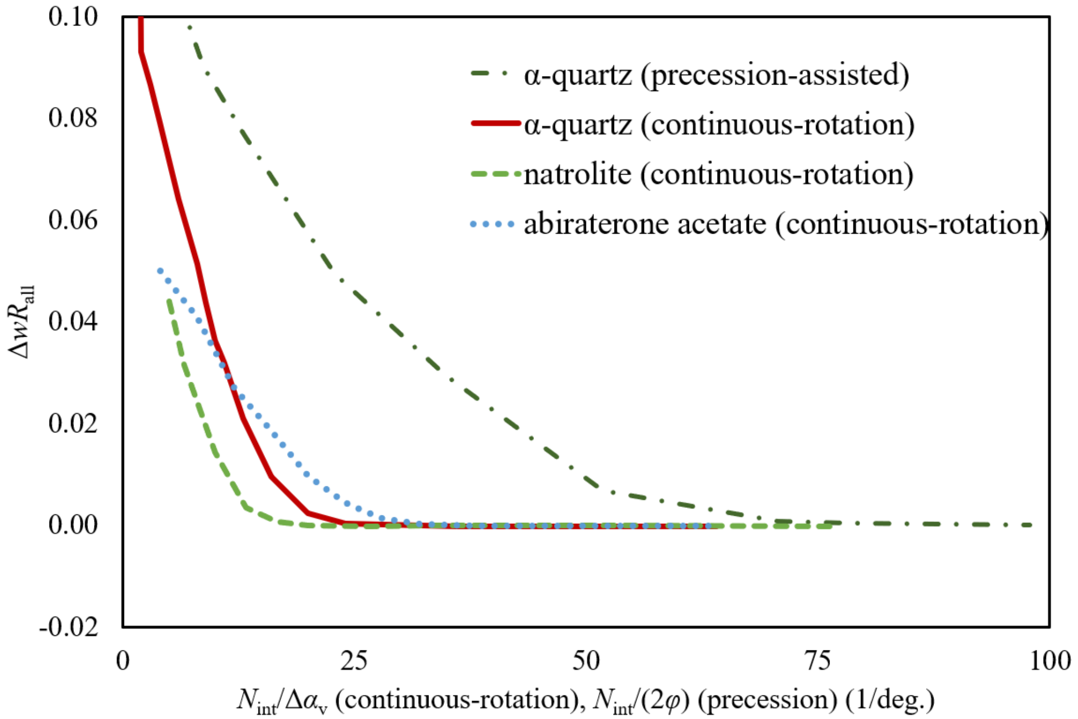

Fig. S5. Number of integration steps.
Dependence of wR_{all} on choice of number of integration steps N_{int} for the numerical integration of calculated rocking curves. $\Delta\alpha_v$ is the angular range covered by a virtual frame. φ is the cone-opening semi-angle (precession angle) for the precession-assisted data set of α-quartz.

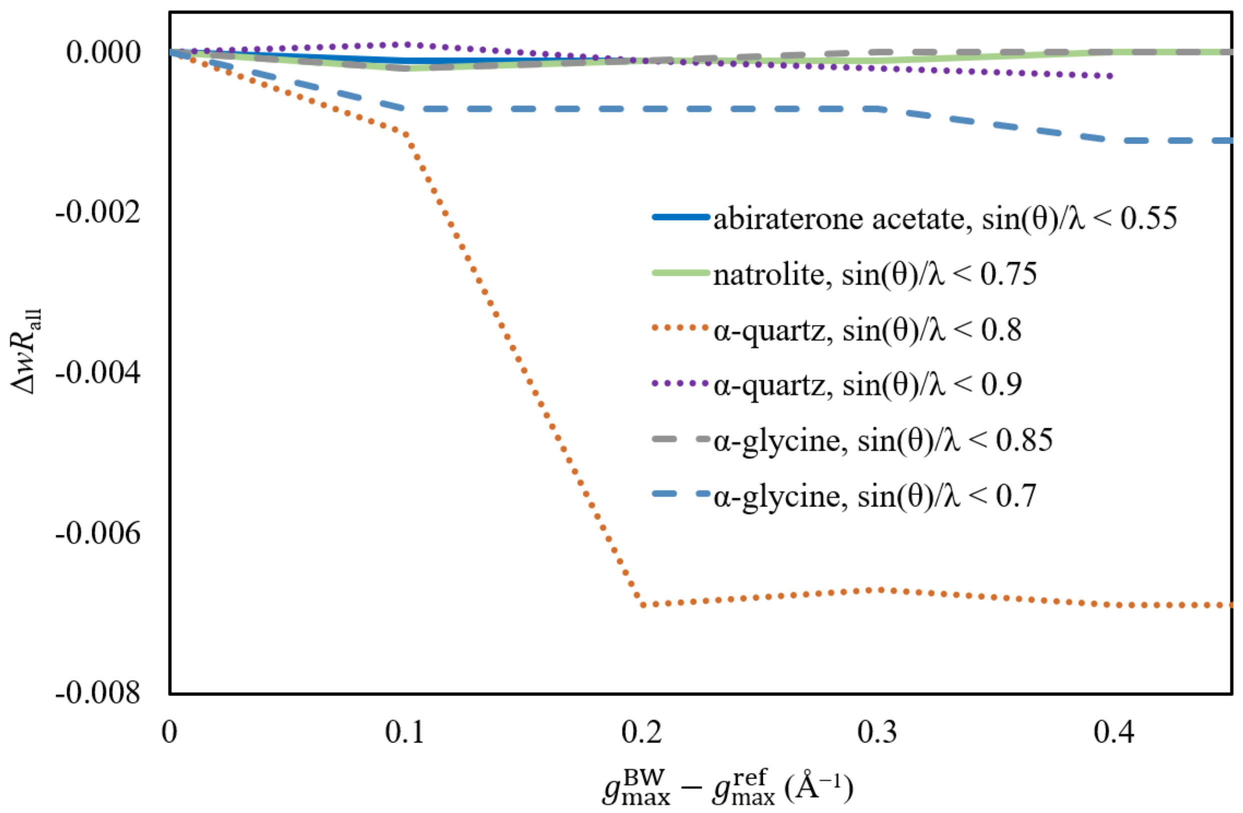

Fig. S6. Dependence of wR_{all} on choice of $g_{\max}^{\mathrm{BW}}$.
The change in ΔwR_{all} as a function of resolution of the dynamical calculations is shown. The term $g_{\max}^{\mathrm{BW}} - g_{\max}^{\mathrm{ref}}$ expresses by how much the resolution of the Bloch wave calculation is increased relative to the resolution of reflections used in the least-squares refinement. If excited high-resolution reflections are not used in the refinement, they should not be excluded from the dynamical calculations because otherwise their contribution to reflections at lower resolutions is neglected. Ignoring high-resolution reflections only has a negligible effect if their intensities are negligible.

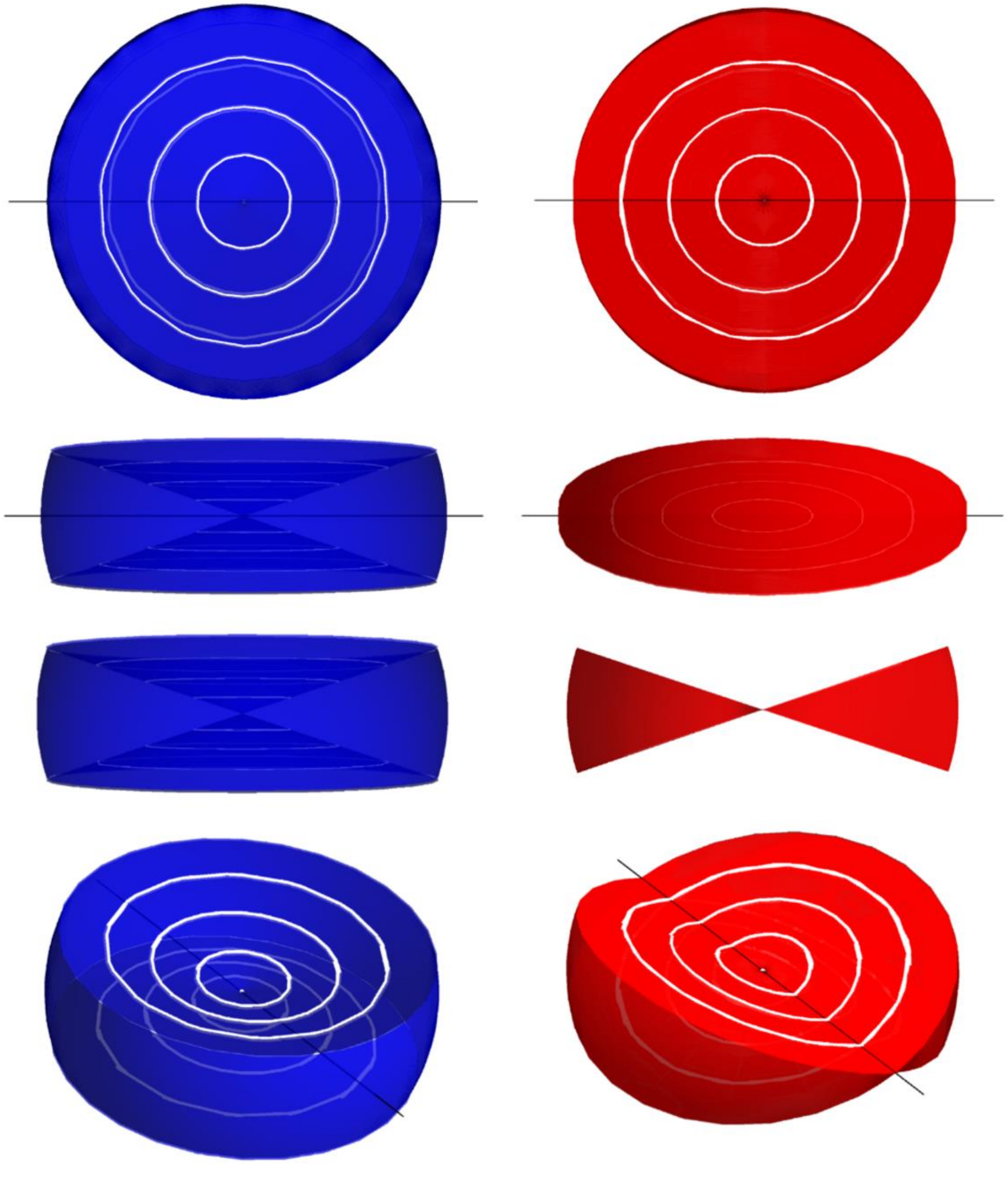

Fig. S7. Precession-assisted and continuous-rotation 3D ED in reciprocal space.
Different views of the reciprocal space volume sampled by the precession movement with a precession angle φ (left, blue volume, $V^*_{precession}$) and of the volume sampled by a goniometer rotation of $\Delta\alpha = 2\varphi$ (right, red, $V^*_{continuous}$). The black line represents the goniometer rotation axis. The ratio of the blue and red volume is $\pi/2$.

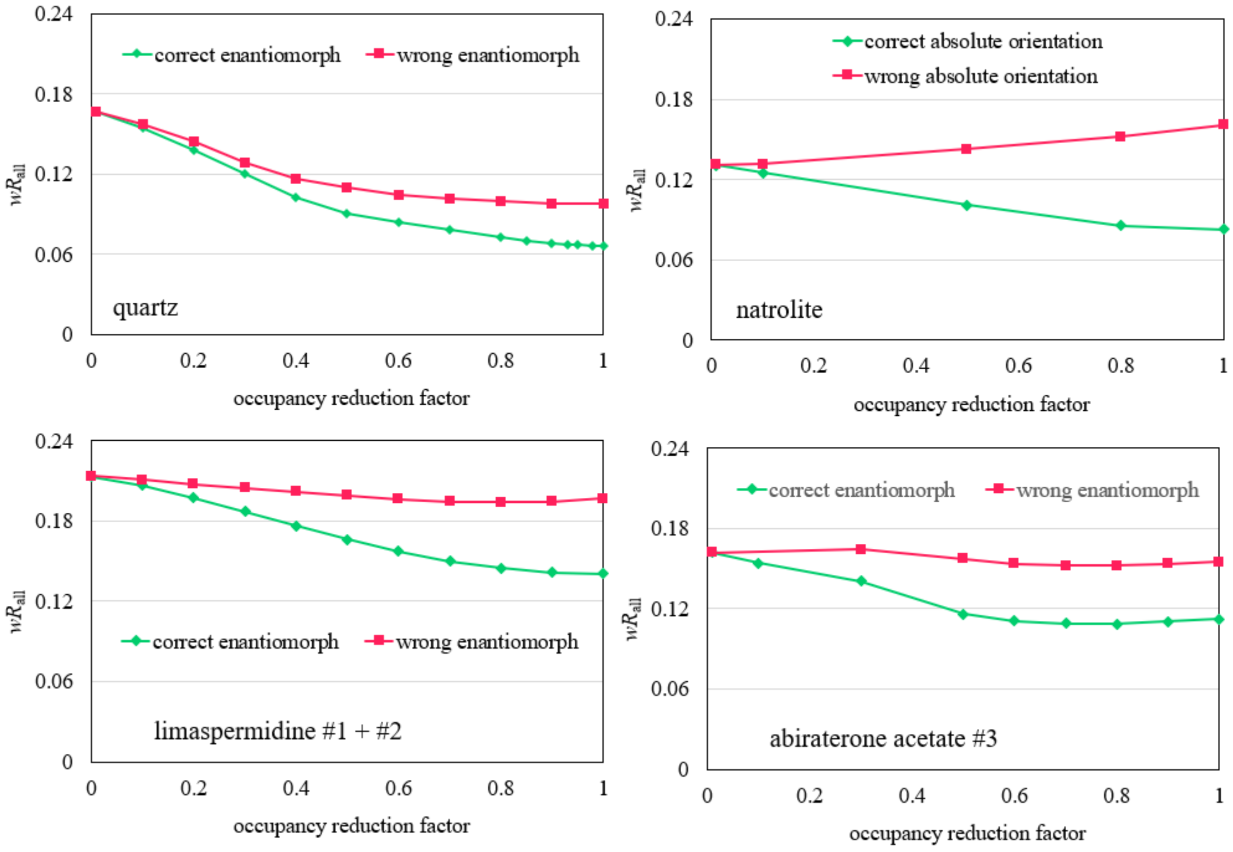

Fig. S8. Frame-based kinematical refinement.

The plots show wR_{all} values for various values of the occupancy reduction factors applied to all atoms in the dynamical refinement. The curves show that as the model intensities become more kinematical, wR_{all} values increase. At the same time, the wR_{all} values for the refinement with the wrong enantiomorph (or wrong absolute orientation in the case of natrolite) become closer to the correct enantiomorph, ultimately converging to the same value in the kinematical limit, as expected.

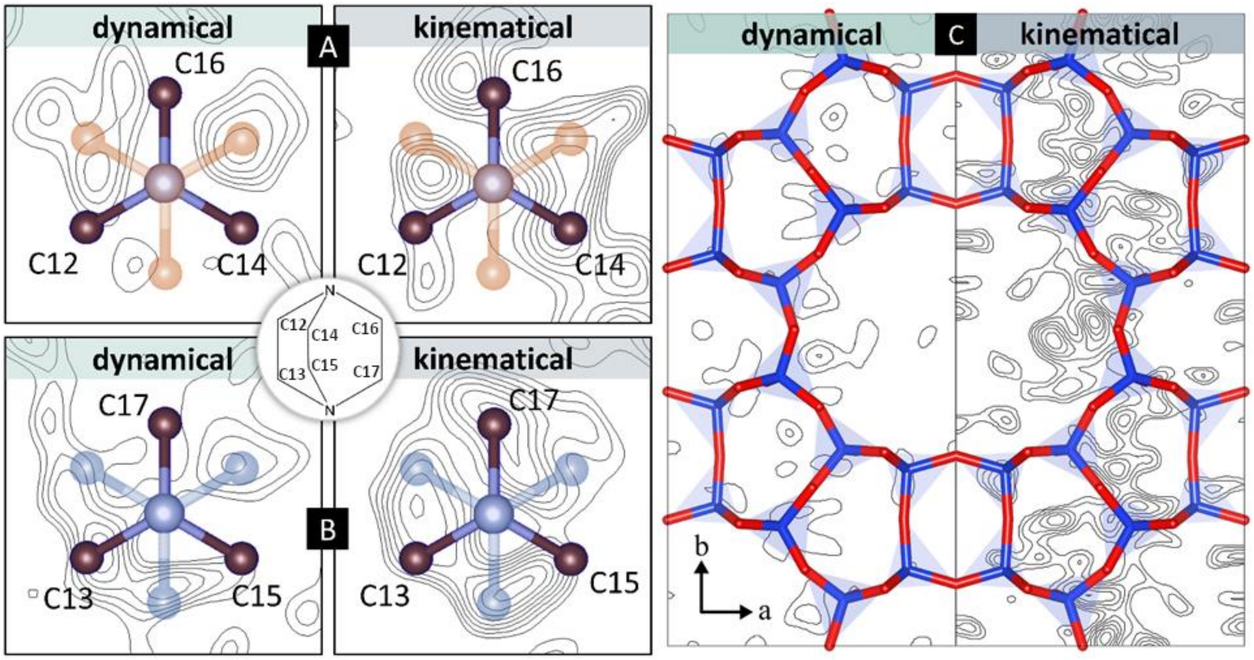

Fig. S9. Difference electrostatic potential maps of CAU-36 and mordenite.
(**A**), (**B**) DESP around DABCO guest molecule in CAU-36 in the refinement with only one orientation of the guest molecule. Panel (**A**) shows the DESP in the plane defined by C12, C14, and C16. Panel (**B**) shows the DESP in the plane defined by C13, C15, and C17. Positive contour lines are plotted in steps of 0.042 e/Å, which corresponds to ½$\sigma[\Delta V(\mathbf{r})]$ of the dynamical refinement. The orientation of a second DABCO molecule, which was not included in the refinement, is shown in transparent colours. In the dynamical refinement (left), five of the six carbon sites are discernible between the carbon atoms indicating the presence of a second DABCO orientation rotated by approximately 60° relative to the predominant orientation. (**C**) DESP map from dynamical (left) and kinematical (right) refinement of mordenite at z = 0.25 with overlaid network structure. Contour lines are plotted in steps of 0.14 e/Å, which corresponds to ½$\sigma[\Delta V(\mathbf{r})]$ of the kinematical refinement. Atom colour codes: carbon: brown; nitrogen: light blue; silicon: blue; oxygen: red

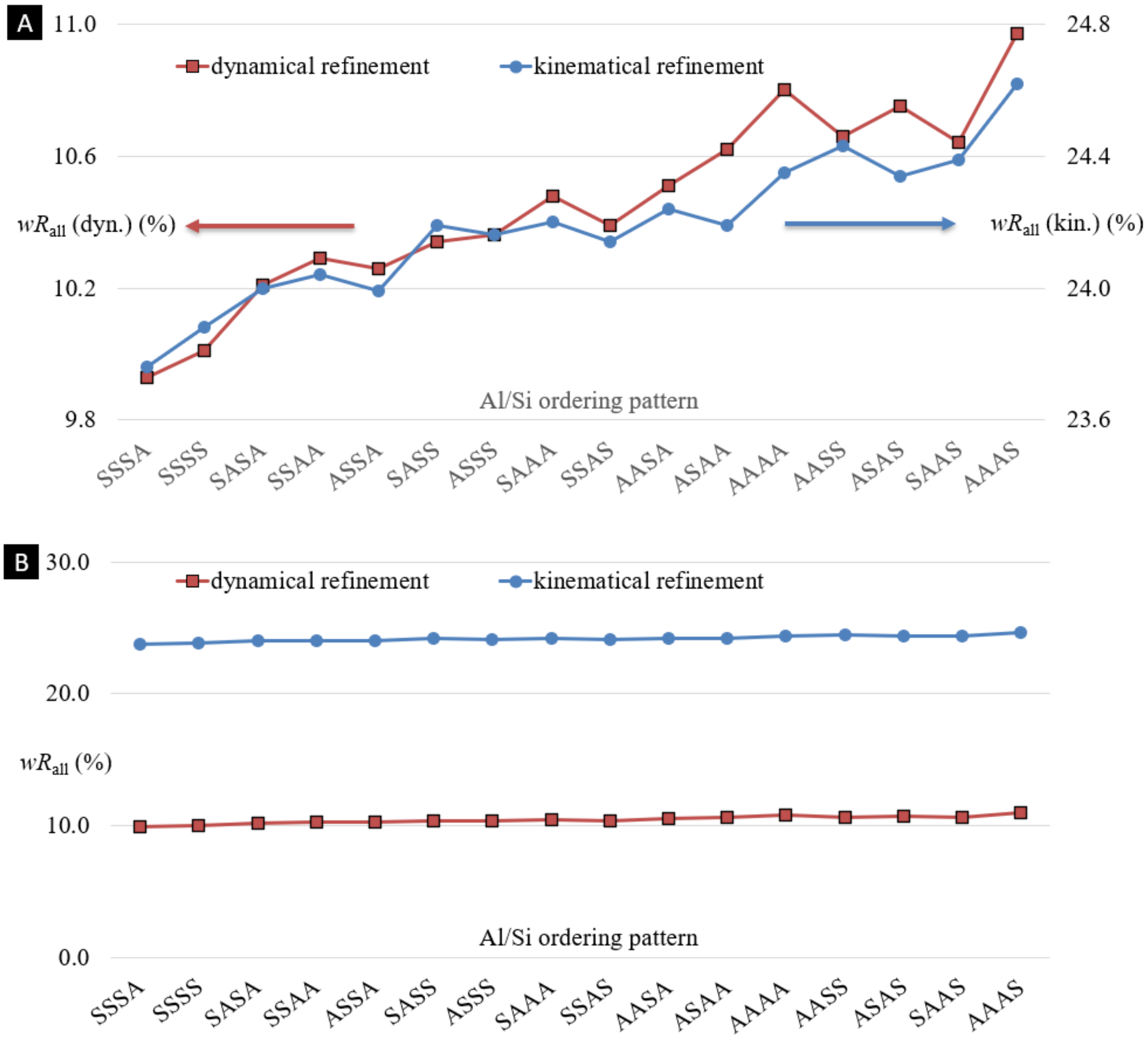

Fig. S10. Identification of Al/Si distribution on tetrahedral sites in albite from *R*-factors.
R-factors of refinements of all 16 Al/Si distributions obtained by dynamical and kinematical refinements. (**A**) wR_{all} of dynamical and kinematical refinement overlaid on different scales. (**B**) wR_{all} of dynamical and kinematical refinement on a common scale.

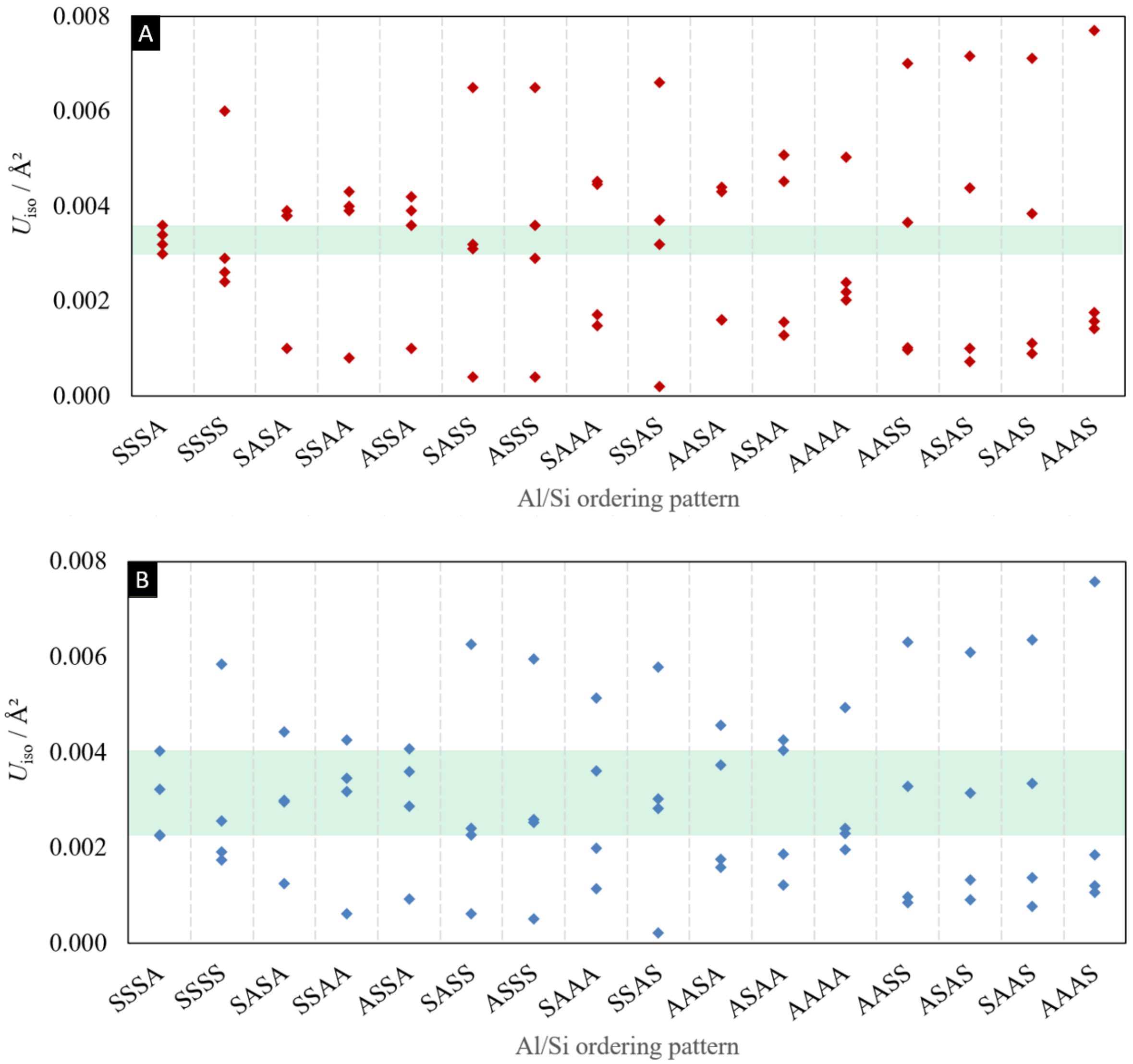

Fig. S11. Identification of Al/Si distribution on tetrahedral sites in albite from U_{iso}.
Refined isotropic displacement parameters U_{iso} of the four tetrahedral sites of the 16 Al/Si distributions obtained by (**A**) dynamical and (**B**) kinematical refinement. In the dynamical refinement, the correct Al/Si assignment (SSSA) is easily identified from the U_{iso} because it is the only refinement where it is almost identical for all four sites (0.0030, 0.0032, 0.0034, 0.0036 Å²). The range of displacement parameters with the same Al/Si distribution is much larger for the kinematical refinement (0.0022, 0.0023, 0.0032, 0.0040 Å²) with a ratio of the largest to smallest U_{iso} of 1.77.

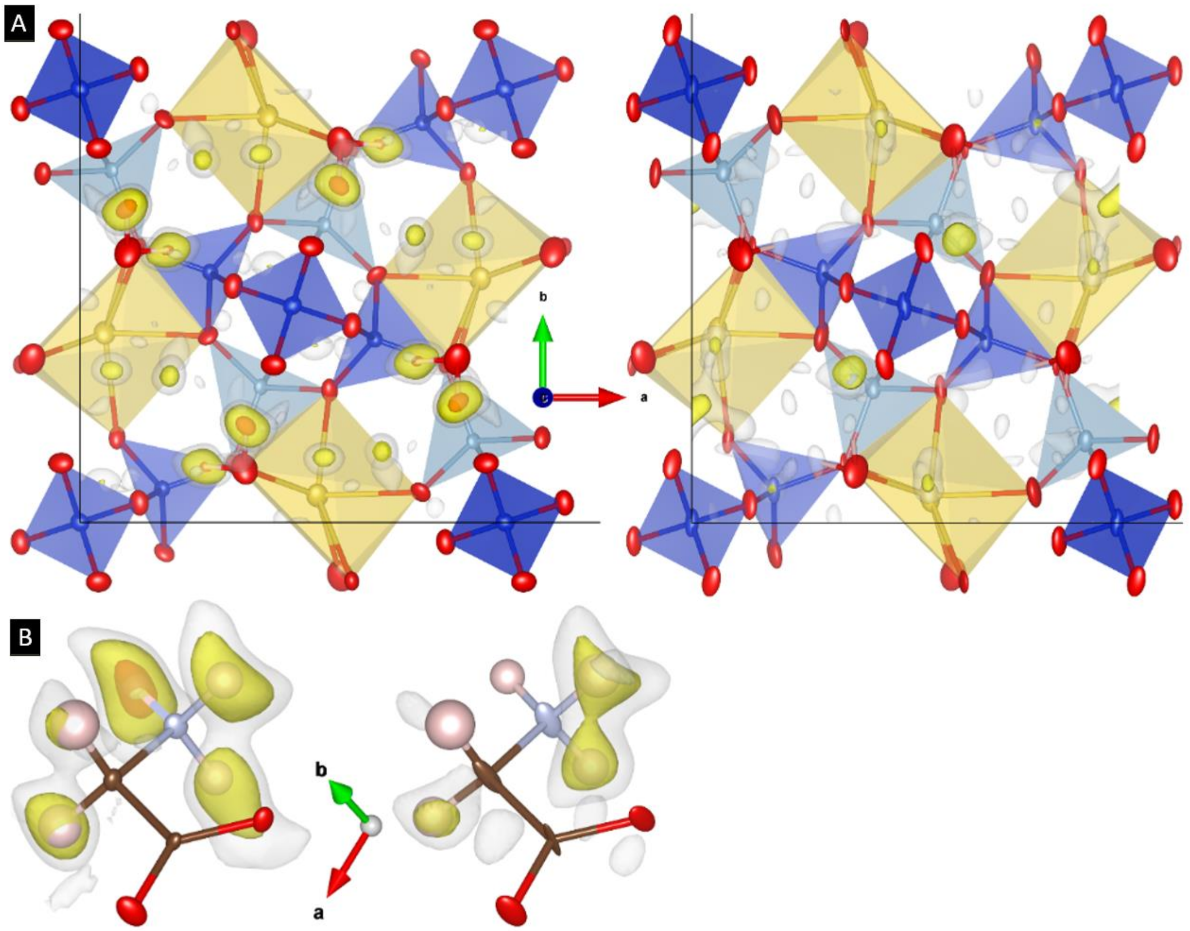

Fig. S12. Detection of hydrogen atoms in difference Fourier maps.
Comparison of difference Fourier maps based on final models without hydrogen atoms shown with overlaid model. The 2σ, 3σ and $5\sigma[\Delta V(\mathbf{r})]$ isosurfaces are shown in grey, yellow and orange, respectively. (**A**) Natrolite: Al, Si, Na and O atoms are represented by light blue, dark blue, yellow, and red ellipsoids, respectively. With the dynamical refinement (left), there are 2 clearly dominating peaks corresponding to the 2 hydrogen sites (indicated by lines originating from O site). With the kinematical refinement (right), the hydrogen sites are among the weakest peaks close to $2\sigma[\Delta V(\mathbf{r})]$ level and the strongest peaks are noise peaks. (**B**) α-glycine: With the dynamical refinement (left), there are 5 clearly dominating peaks corresponding to the 5 hydrogen sites. With the kinematical refinement (right), there is no significant peak stronger than $2\sigma[\Delta V(\mathbf{r})]$ close to the expected positions of 2 hydrogen sites. Also note the more realistic shape of the ADP ellipsoids of the dynamical refinements. Atom colour codes: carbon: brown; nitrogen: light blue; hydrogen: pink; silicon: blue; oxygen: red; sodium yellow

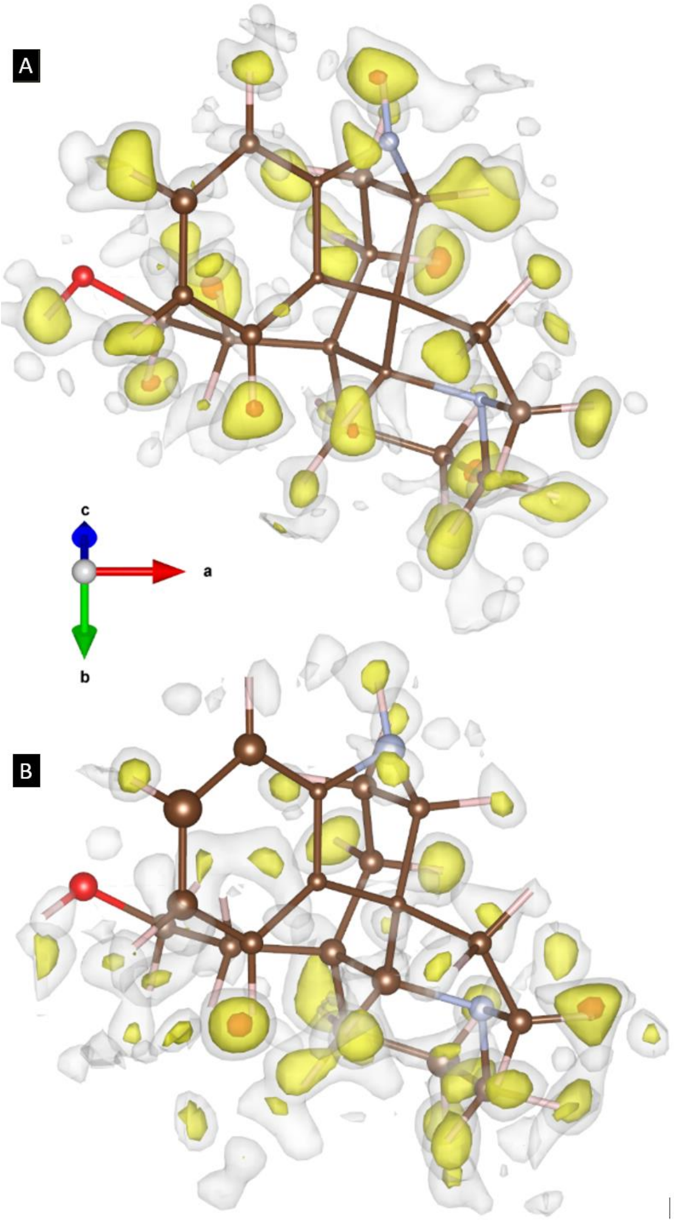

Fig. S13. Detection of hydrogen atoms in difference Fourier maps of (+)-limaspermidine. Comparison of difference Fourier maps of (+)-limaspermidine based on final models without hydrogen atoms shown with overlaid model. The 2σ, 3σ and $5\sigma[\Delta V(\mathbf{r})]$ isosurfaces are shown in grey, yellow and red, respectively. (**A**) The difference Fourier map from the dynamical refinement confirms all of the 26 expected hydrogen sites above the 3σ-level. (**B**) The difference Fourier map from the kinematical refinement confirms 19 expected hydrogen sites above the 3σ-level and further 3 hydrogen sites above the 2σ-level. Atom colour codes: carbon: brown; nitrogen: light blue; hydrogen: pink

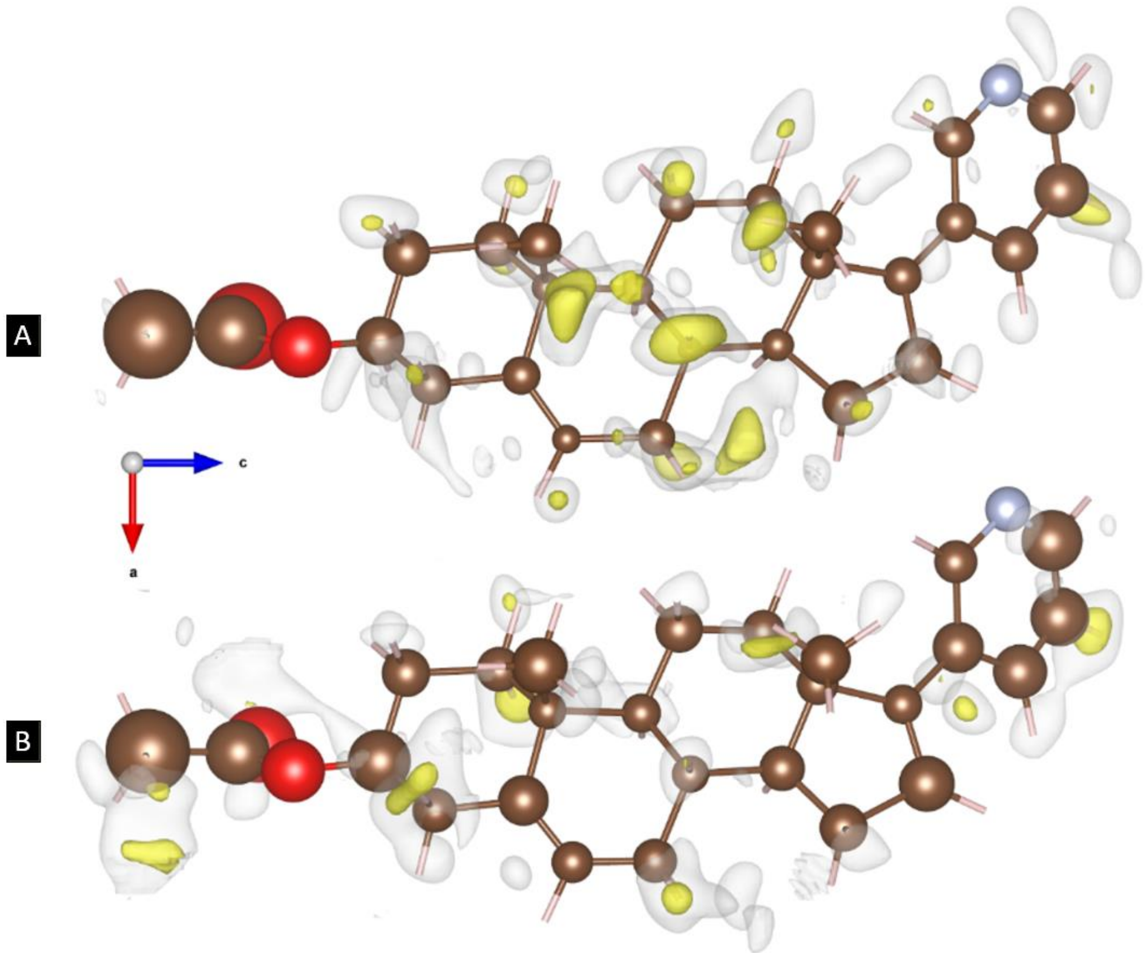

Fig. S14. Detection of hydrogen atoms in difference Fourier maps of abiraterone acetate. Comparison of difference Fourier maps of abiraterone acetate based on final models without hydrogen atoms shown with overlaid model. The 2σ and $3\sigma[\Delta V(\mathbf{r})]$ isosurfaces are shown in grey and yellow, respectively. (**A**) The difference Fourier map from the dynamical refinement confirms 25 of 33 expected hydrogen sites. (**B**) The difference Fourier map from the kinematical refinement confirms 14 of 33 expected hydrogen sites. Atom colour codes: carbon: brown; nitrogen: light blue; hydrogen: pink. Hydrogen positions shown only with the pink-coloured sticks indicating the bond between the carbon and hydrogen atoms.

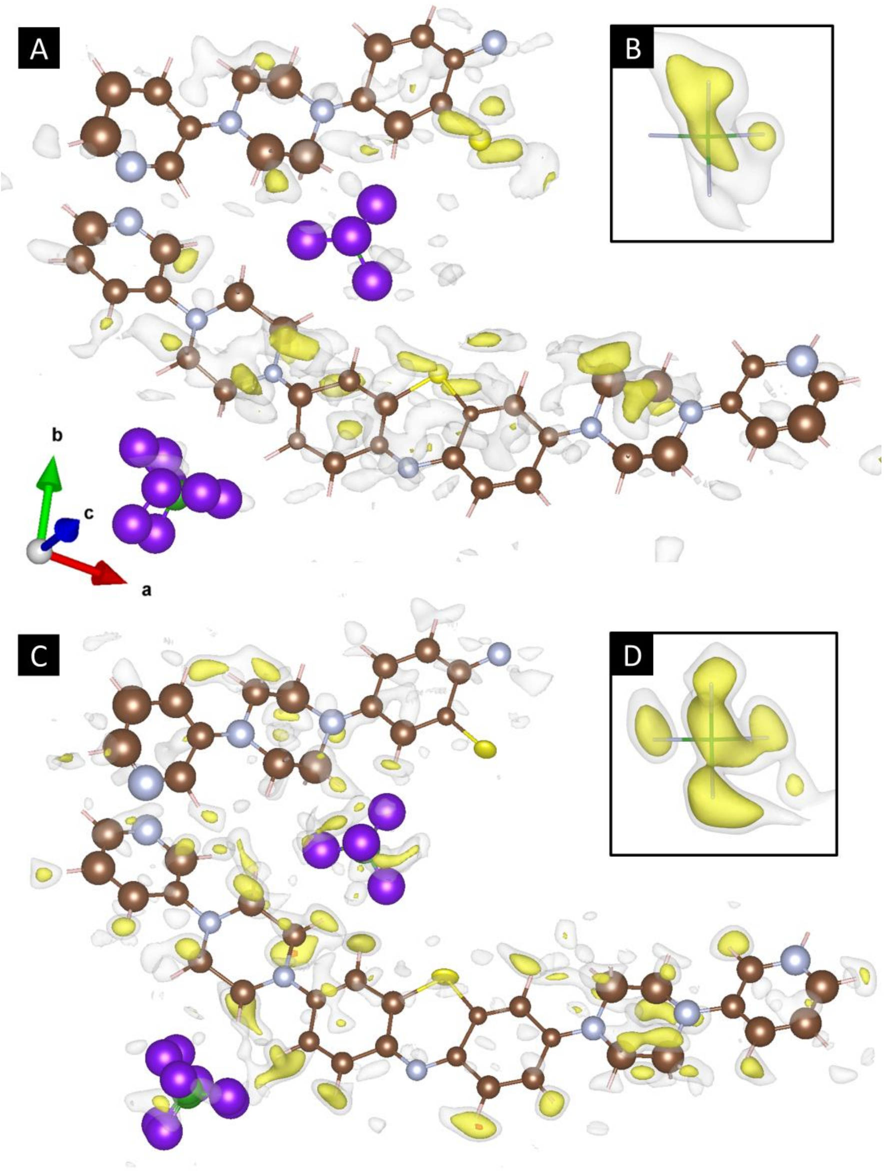

Fig. S15. Difference Fourier maps of MBBF4 from dynamical and kinematical refinement. Comparison of difference Fourier maps of MBBF4 based on final models without hydrogen atoms shown with overlaid model. The 2σ, 3σ and $5\sigma[\Delta V(\mathbf{r})]$ isosurfaces are shown in grey, yellow and orange, respectively. (**A**) The difference Fourier map from the dynamical refinement confirms 30 of 46 expected hydrogen sites. (**B**) The difference Fourier map from the kinematical refinement confirms only 6 of 46 expected hydrogen sites. Insets (**C**) and (**D**) show the difference Fourier maps of the final model calculated without the disordered BF_4 counterion (removed molecule overlaid). The molecule geometry and orientation is unambiguously identified with the dynamical refinement (**C**). Atom colour codes: carbon: brown; nitrogen: light blue; hydrogen: pink; boron: dark green, fluorine: purple. Hydrogen positions shown only with the pink-coloured sticks indicating the bond between the carbon and hydrogen atoms.

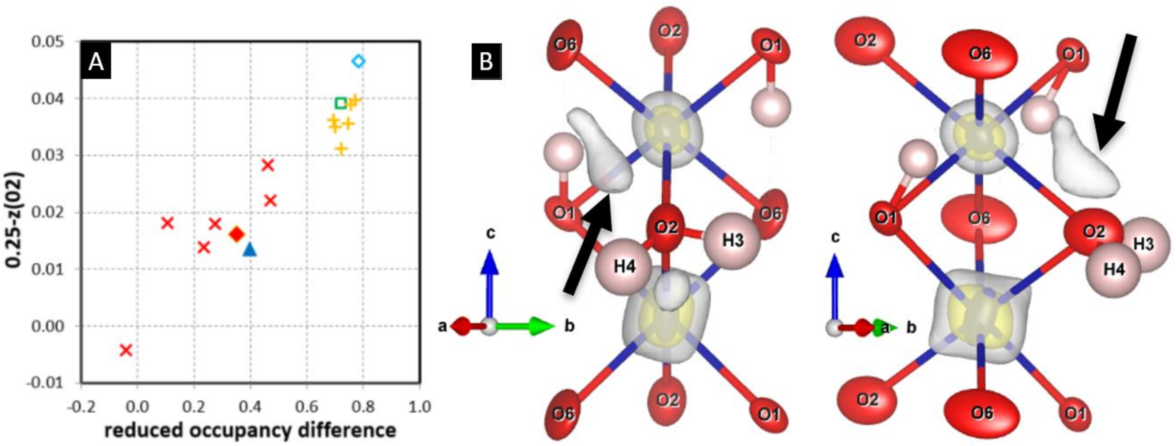

Fig. S16. Beam induced disorder in CAP.
(**A**) Linear relationship between reduced occupancy difference of Co1 and Co2 based on refined model of dynamical refinement (red diamond) and kinematical refinement (blue triangle). Other data points taken from Fig. S4b in reference[35]. (**B**) Two different views of the cobalt environment in the final model determined by dynamical refinement shown together with the final difference Fourier map. Grey isosurfaces correspond to $2\sigma[\Delta V(\mathbf{r})]$, which is 0.253 e/Å. Hydrogen atoms bonded to O2 at the top and bottom of the figures are omitted. Black arrows point to a maximum in the potential map at the expected coordinates of the H4' site with an expected occupancy of about 28%. Atom colour codes: cobalt: dark blue; oxygen: red; hydrogen: pink.

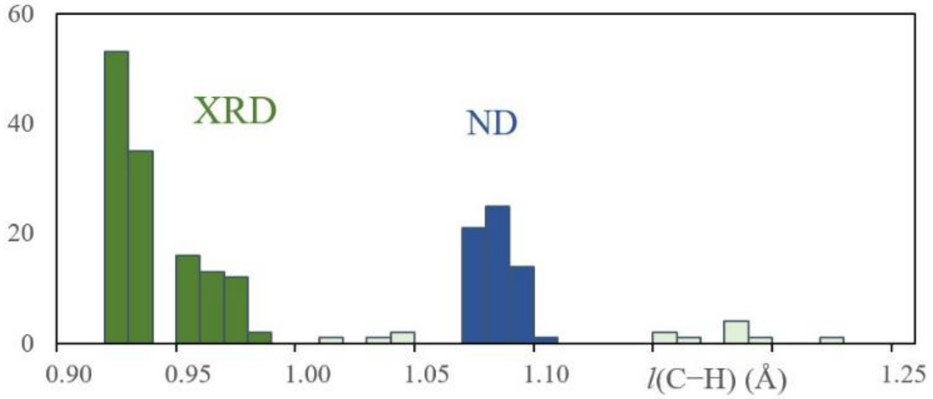

Fig. S17. Commonly used constraints on C−H distances.
Histogram of C−H distances *l*(C−H) in structures deposited in the CSD with the keyword "electron diffraction". The bin width is 0.01 Å. The two dominating distances can be assigned to the commonly used constraints in XRD and ND-based models.

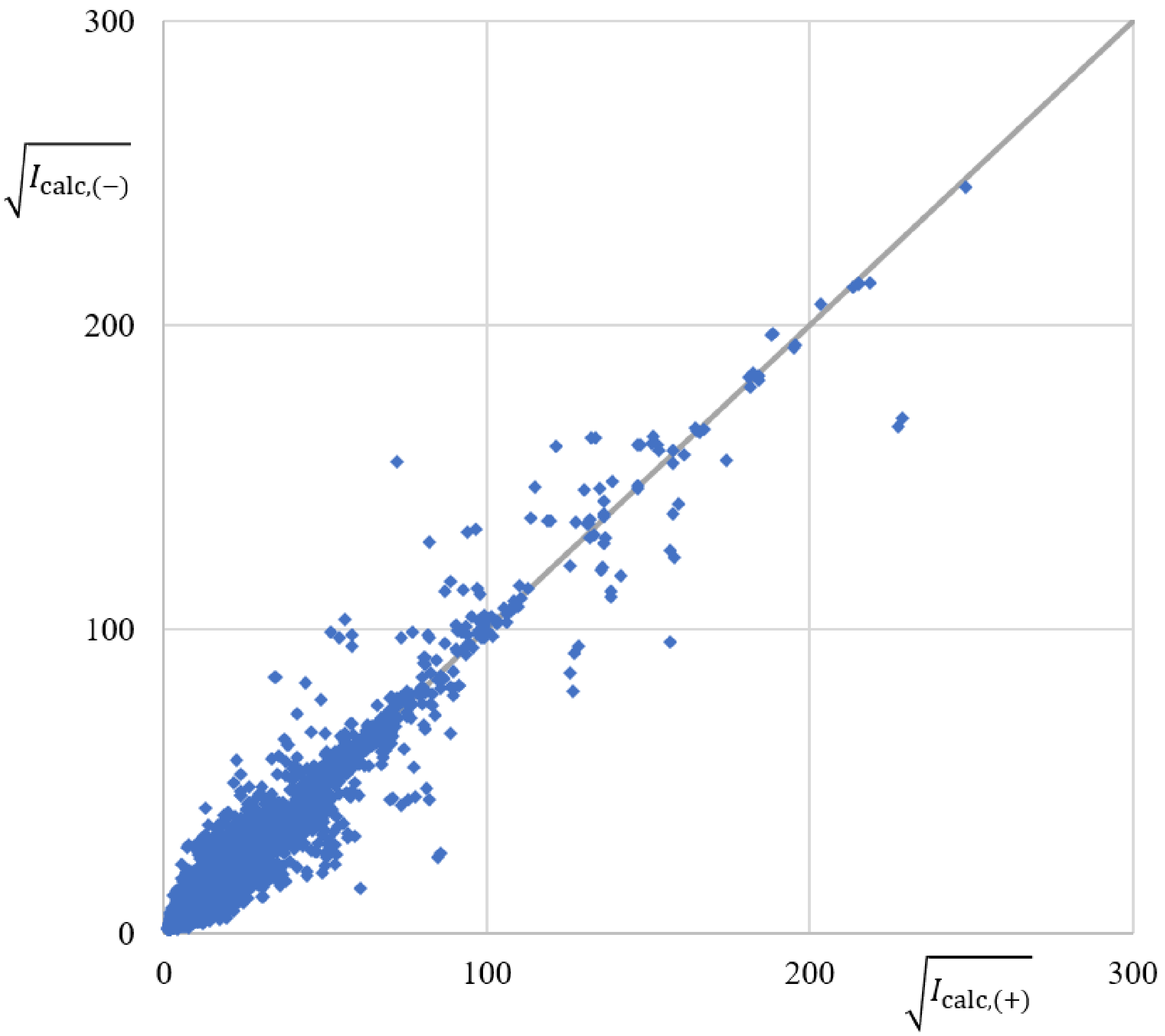

Fig. S18 Chirality-dependent intensities of limaspermidine.
Comparison of 4612 calculated integrated intensities using the diffraction geometry of the first 59 OVFs of the measurement described in Table S15. A thickness of 1000 Å was used for the calculations. The structure models of (+)-limaspermidine and (−)-limaspermidine used for the calculation of the intensities are related by exact inversion. Calculated intensities within the kinematical approximation are thus identical for (+)-limaspermidine and (−)-limaspermidine.

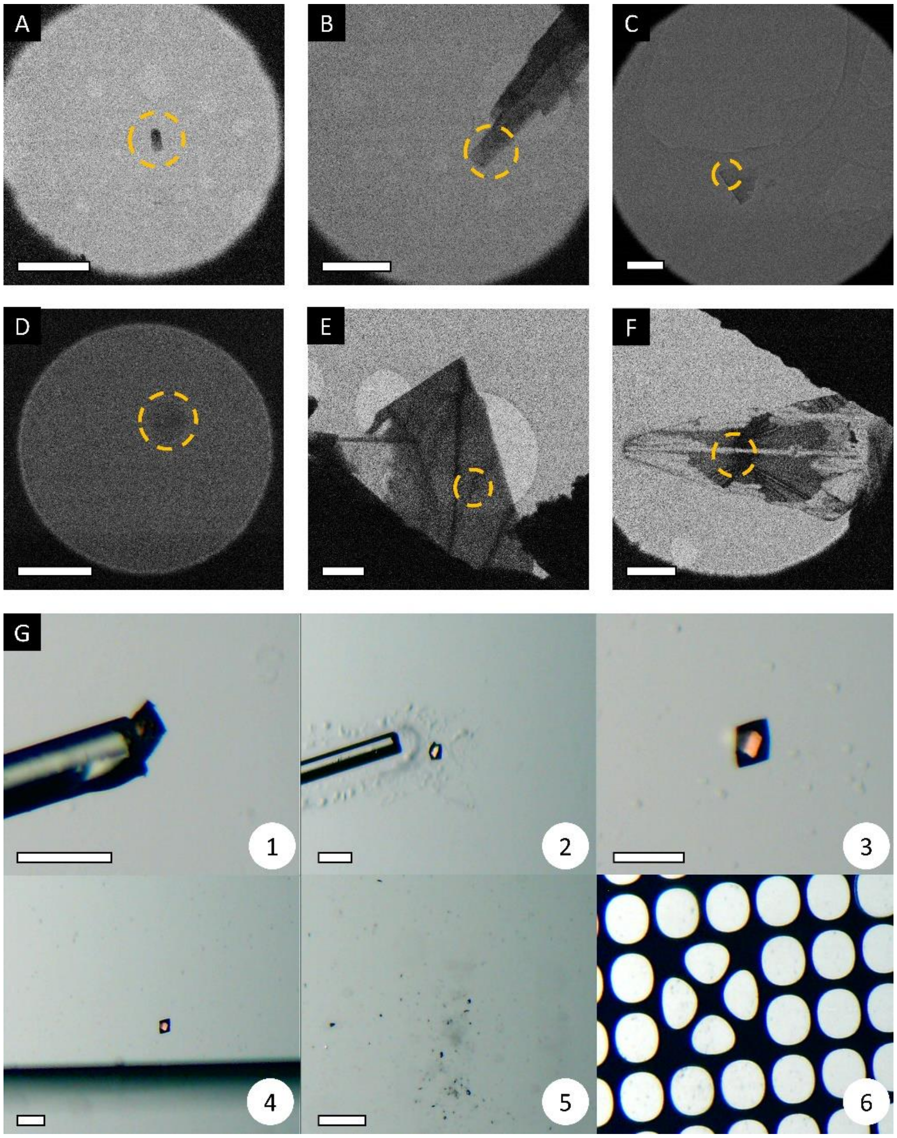

Fig. S19. TEM images of the measured crystallites.
Scale bar width of TEM images (A-F) is 1 µm. The dashed circle indicates the illuminated area during data collection. Scale bar width of light microscope images (G, steps 1-5) is about 100 µm. (**A**) Crystal of α-quartz used for the continuous-rotation and precession-assisted 3D ED measurements. The quasi-parallel beam illuminating the sample during the measurement had a diameter of about 750 nm (dashed circle). (**B**) Part of a natrolite crystal used for the continuous-rotation and precession-assisted 3D ED measurements. Beam diameter: 750 nm. (**C**), (**D**) Two crystals of CAP were measured with an experimental setup similar to the original ADT (automated diffraction tomography) data acquisition in fine steps of $\Delta\alpha = 0.1°$, but without operating in scanning TEM mode. Beam diameter: 900 nm. (**E**), (**F**) TEM images of crystals #3 and #1 of abiraterone acetate that were crystallised *in situ* on the grid. Beam diameter: 850 nm. The pronounced mosaicity was a challenge during the data reduction, but the absolute structure could still be unambiguously determined. (**G**) Micrographs of the single crystal (diameter ~50 µm) of STW_HPM-1. The procedure is shown how the crystal was dismounted (after the single-crystal XRD measurement), crushed, and transferred to a holey carbon film for 3D ED measurements.

Supplementary Tables

Tab. S1. Dependence of refinement on virtual frame parameters of α-quartz.

Refinements of α-quartz with different choices of virtual frame parameters. All coordinates, anisotropic displacement parameters of non-hydrogen atoms and isotropic displacement parameters of hydrogen atoms were freely refined without any constraints or restraints, except for symmetry constraints on the silicon site. Meaning of used symbols is the same like in other sections except for RMSD, which here refers to the root-mean square deviation of the atom positions from XRD-based reference positions [62]. Lines marked with an asterisk (*) indicate that the refinement converged with at least one non-positive definite ADP tensor.

N_F	N_o	$\Delta\alpha_v$ (deg.)	$\Delta\alpha_o$ (deg.)	N_{OVF}	N_{par}	N_{obs}	N_{all}	R_{obs}	wR_{all}	RMSD (Å)
1	0	1.0	0.0	100	115	370	674	0.067	0.075	0.007
2	0	2.0	0.0	50	65	476	815	0.075	0.083	0.008
2	1	2.0	1.0	99	114	937	1607	0.072	0.083	0.006
3	0	3.0	0.0	33	48	488	843	0.072	0.082	0.009
3	1	3.0	1.0	50	65	732	1247	0.073	0.084	0.005
3	2	3.0	2.0	98	113	1429	2439	0.075	0.087	0.007
4	0	4.0	0.0	25	40	485	826	0.078	0.094	0.012
4	1	4.0	1.0	33	48	635	1086	0.075	0.088	0.005
4	2	4.0	2.0	49	64	952	1622	0.077	0.089	0.010
4	3	4.0	3.0	97	112	1894	3235	0.077	0.088	0.009
8	1	8.0	1.0	14	29	671	1532	0.085	0.095	0.009
16	4	16.0	4.0	8	23	634	1306	0.086	0.095	0.012 *
25	25	25.0	25.0	4	19	420	758	0.093	0.102	0.015 *
50	50	50.0	50.0	2	17	237	405	0.099	0.111	0.023 *

Tab. S2. Dependence of refinement on virtual frame parameters of α-glycine.

Refinements of α-glycine with different choices of virtual frame parameters. Description like in previous table. XRD-based reference coordinates were taken from [63]. Lines marked with an asterisk (*) indicate that the refinement converged with one non-positive definite ADP tensor.

N_F	N_o	$\Delta\alpha_v$ (deg.)	$\Delta\alpha_o$ (deg.)	N_{OVF}	N_{par}	N_{obs}	N_{all}	R_{obs}	wR_{all}	RMSD (Å) (non-H)	RMSD (Å) (all atoms)
1	0	0.36	0.00	151	217	778	942	0.085	0.107	0.022	0.142
3	0	1.07	0.00	45	111	527	649	0.083	0.100	0.019	0.123
3	1	1.07	0.36	69	135	806	995	0.081	0.099	0.020	0.105
3	2	1.07	0.71	135	201	1569	1947	0.083	0.102	0.019	0.093
7	0	2.49	0.00	15	81	401	490	0.069	0.085	0.018 *	0.128 *
7	1	2.49	0.36	19	85	516	630	0.079	0.098	0.019	0.151
7	2	2.49	0.71	24	90	656	800	0.078	0.098	0.017	0.114
7	3	2.49	1.07	31	97	861	1041	0.079	0.097	0.018	0.118
7	4	2.49	1.42	35	101	963	1169	0.080	0.100	0.018	0.112
7	5	2.49	1.78	55	121	1506	1836	0.078	0.098	0.018	0.115
7	6	2.49	2.13	103	169	2814	3427	0.079	0.099	0.018 *	0.106 *
19	−1	6.76	−0.36	7	73	698	820	0.078	0.096	0.017	0.114

Tab. S3. Comparison of XDS/SHELXL and PETS/Jana2006.
Kinematical refinements based on the data sets used in this study. P and X in the label refer to the data reduction with PETS2 and XDS, respectively. J and S refer to the refinement with Jana2006 and SHELXL, respectively. P+J* indicates the kinematical refinement used in the main article. *Compl.* is the completeness, N_{par} is the number of refinement parameters. The data reduction of data set #1 (Fig. S11F) of abiraterone acetate failed so that only 4 out of 5 data sets were used for the respective refinements. Anisotropic displacement parameters (ADPs) in abiraterone acetate and MBBF4 were not refined with Jana2006. Using restraints on the displacement parameters in abiraterone acetate, a refinement with ADPs was performed with SHELXL. For the sake of completeness, the parameters extracted from the previously published kinematical refinements were included in the table. Note that only in the case of mordenite the diffraction data used in this study are identical to the data on which the published model from reference[20] is based.

Label	Compound	g_{max}^{ref} (Å^{-1})	d_{min} (Å)	*Compl.* (%)	N_{obs}	N_{all}	N_{par}	R_{obs}	wR_{all}	$wR2_{all}$
	Refinements based on the data sets as described in this study:									
P+J*	α-quartz	1.60	0.63	100	208	318	16	0.106	0.130	
P+J		1.66	0.60	100	215	363	16	0.106	0.131	
X+J		1.66	0.60	98.2	222	349	16	0.101	0.143	
X+S		1.66	0.60	98.5	248	349	16	0.161		0.359
P+J*	natrolite	1.50	0.67	98.4	1135	1979	93	0.119	0.133	
X+J		1.50	0.67	92.2	1010	1908	93	0.102	0.147	
X+S		1.54	0.65	95.2	1175	2038	96	0.112		0.303
P+J*	mordenite	1.40	0.71	92.3	1079	2199	95	0.168	0.197	
P+J		1.28	0.78	95.8	908	1551	95	0.166	0.189	
X+S		1.28	0.78	92.5	1140	1585	96	0.160		0.4480
P+J*	CAP	1.40	0.71	92.4	1111	1864	153	0.165	0.176	
X+J		1.34	0.75	90.5	1067	1621	147	0.169	0.231	
X+S		1.34	0.75	89.1	1188	1621	133	0.176		0.4797
P+J*	α-glycine	1.70	0.59	40.0	474	619	67	0.136	0.159	
P+J		1.42	0.70	50.0	383	468	67	0.133	0.151	
X+S		1.42	0.70	41.8	366	456	52	0.175		0.497
P+J*	abiraterone acetate	0.96	1.04	91.5	932	1902	118	0.163	0.188	
X+J		0.96	1.04	87.6	926	1820	118	0.158	0.218	
X+S		1.00	1.00	86.6	1221	2074	244	0.140		0.408
P+J*	MBBF4	1.00	1.00	84.3	1979	3934	277	0.294	0.332	
	Previously published refinements based on a superset of data sets[21–23]:									
X+S	α-glycine	1.42	0.70	85.0	561	850	51	0.219		0.518
X+S	carbamazepine	1.00	1.00	88.3	978	1018	164	0.192		0.390
X+S	(+)-limaspermidine	1.30	0.77	93.0	1669	3430	90	0.242		0.431
X+S	MBBF4	1.12	0.89	86.1	3774	5649	652	0.296		0.560

Tab. S4. Duration of dynamical refinement cycles.
The elapsed real time t is given for a single dynamical refinement cycle in minutes. t_R is the elapsed real time of a single cycle of dynamical calculations without refining any parameters. The latter thus represents the time to only determine I_{calc} and R-factors. Refinements were performed on a computer with octa-core CPU (Intel Core i9-9900K) and 32 GB main memory.

Compound	V_P (Å³)	g_{max}^{ref} (Å^{-1})	g_{max}^{BW} (Å^{-1})	N_{int}	N_{all}	$N_{parameters}$	N_{frames}	t_R (min.)	t (min.)
α-quartz	113	1.60	2.00	64	1801	114	99	< 1	< 1
α-quartz (precession)	113	1.60	2.00	128	2613	116	101	< 1	< 1
mordenite	1425	1.30	1.42	42	6285	177	82	1	4
natrolite	563	1.50	1.60	46	5175	253	161	< 1	3
natrolite (precession)	563	1.50	1.60	96	5747	253	161	< 1	5
CAP	712	1.40	1.60	64	8889	391	237	2	8
α-glycine	305	1.70	1.90	42	1540	126	60	< 1	< 1
carbamazepine	1145	1.28	1.30	42	11583	407	231	2	9
(+)-limaspermidine	1594	1.20	1.25	46	11683	212	116	1	16
abiraterone acetate #03	2186	1.10	1.10	38	5535	197	80	1	11
abiraterone acetate #06	2186	1.10	1.10	38	9924	281	164	2	20
abiraterone acetate	2186	0.96	1.00	42	10960	370	247	2	18
MBBF4 #4	4487	1.00	1.12	32	2305	325	40	1	23
MBBF4 #4	4487	1.00	1.00	32	2305	325	40	1	14
MBBF4	4487	1.00	1.00	30	14629	537	245	6	83
MBBF4	4487	1.00	1.12	21	14629	537	245	9	99
MBBF4	4487	1.00	1.12	32	14629	537	245	9	126
MBBF4	4487	1.00	1.12	54	14629	537	245	9	187
MBBF4	4487	1.00	1.12	64	14629	537	245	9	242

Tab. S5. Frame-based kinematical refinement.

R-values for dynamical, frame-based kinematical and normal kinematical refinements of several structures. Results based on data reduction with PETS2 and refinement with JANA2006. Note that the number of refinement parameters is identical for the dynamical and frame-based kinematical refinement.

Compound	MwR_{all} dynamical	MwR_{all} frame-based kinematical	wR_{all} normal kinematical
α-quartz	0.061	0.168	0.130
mordenite	0.105	0.206	0.207
natrolite	0.072	0.122	0.133
CAP	0.101	0.230	0.176
α-glycine	0.092	0.161	0.159
carbamazepine	0.112	0.248	0.187
(+)-limaspermidine	0.116	0.201	0.160
abiraterone acetate	0.089	0.179	0.188
MBBF4	0.133	0.281	0.332

Tab. S6. α-quartz.

Relevant parameters of the measurement, data reduction and refinement results of α-quartz. One crystal was measured in a quasi-simultaneous way using precession-assisted and (stepwise) continuous-rotation 3D ED so that the refinement results are directly comparable (see Methods). Reference atomic distances for the calculation of RMSD were taken from reference[64], CCDC code 1471429. Atom colour codes: silicon: inside the blue tetrahedron; oxygen: red

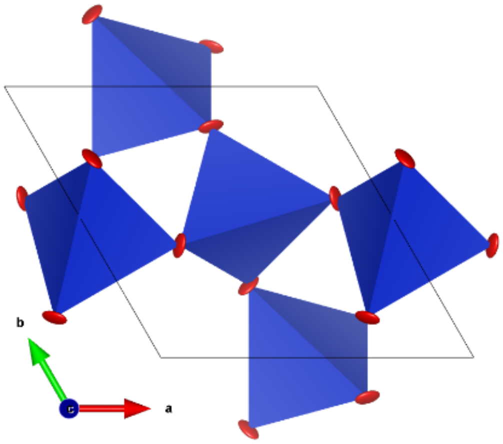

**Measurement of α-quartz**				
Microscope	FEI Tecnai G2 20			
Detector (type)	Olympus SIS Veleta (CCD)			
3D ED data sets	1 continuous-rotation<br>1 precession-assisted			
λ (Å)	0.02508			
T (K)	293			
α_{min}, α_{max}, $\Delta\alpha$ (deg.)	−50.0, 50.0, 1.0			
η_{exp}, η_{gap} (deg.)	1.0, 0.0			
**Crystal**				
Empirical formula	SiO_2			
Z	3			
Space group	$P3_221$			
a, b, c (Å)	4.9226, 4.9226, 5.4003			
α, β, γ (deg.)	90, 90, 120			
V (Å³)	113.33			
Mosaicity (deg.)	0.05			
Completeness	100.0 %			
**Dynamical refinement setup for continuous-rotation 3D ED**			**Precession-assisted 3D ED**	
$\Delta\alpha_v$, $\Delta\alpha_o$ (deg.)	2.0, 1.0		φ (deg.)	0.92
N_F, N_O, N_{OVF}	2, 1, 99		N_{frames}	101
R_{Sg}^{max}, D_{Sg}^{min}, g_{max}^{BW}	0.9, 0.0015 Å^{-1}, 1.6 Å^{-1}		R_{Sg}^{max}, D_{Sg}^{min}, g_{max}^{BW}	0.9, 0.0015 Å^{-1}, 1.6 Å^{-1}
**Refinement**	**dynamical**	**kinematical**	**dynamical**	**kinematical**
g_{max}^{ref} (Å^{-1})	1.60	1.60	1.60	1.60
$\sin(\theta_{max})/\lambda$ (Å^{-1})	0.80	0.80	0.80	0.80
d_{min} (Å)	0.63	0.63	0.63	0.63
N_{obs}, N_{all}	994, 1710	208, 318	1019, 2613	191, 317
Parameters	114	16	116	13
R_{obs}, MR_{obs}	0.057, 0.057	0.106, 0.106	0.054, 0.056	0.107, 0.107
R_{all}, MR_{all}	0.105, 0.079	0.156, 0.156	0.173, 0.104	0.168, 0.168
wR_{all}, MwR_{all}	0.066, 0.061	0.130, 0.130	0.065, 0.056	0.113, 0.113
$\sigma[\Delta V(\mathbf{r})]$ (e/Å)	0.097	0.193	0.090	0.225
$\min[\Delta V(\mathbf{r})]$, $\max[\Delta V(\mathbf{r})]$	−0.272, 0.292	−0.615, 0.562	−0.220, 0.318	−0.685, 0.519
RMSD (Å)	0.001	0.012	0.008	0.004

Tab. S7. Albite.

Relevant parameters of the measurement, data reduction and refinement results of albite. 16 data sets were obtained from 9 individual crystals. Only 3 were selected for the dynamical refinement, whereas the kinematical refinement is based on the published HKL file[19]. The data sets used for the dynamical refinement are labelled "00", "02" and "14" by the data set authors. Reference atomic distances for the calculation of RMSD were taken from reference[65], CCDC code 77421. Atom colour codes: silicon: blue; sodium: yellow; oxygen: red

**Measurement of albite**		
Microscope	Phillips CM200	
Detector (type)	PSI JUNGFRAU (HPD)	
3D ED data sets	3 (16) continuous-rotation	
λ (Å)	0.02508	
T (K)	293	
α_{min}, α_{max}, $\Delta\alpha$ (deg.)	−40.0, 40.0, 0.001	
η_{exp}, η_{gap} (deg.)*	0.001, ~0.0	
**Crystal**		
Empirical formula	$NaAlSi_3O_8$	
Z	2	
Space group	P−1	
a, b, c (Å)	7.13, 7.38, 7.64	
α, β, γ (deg.)	115.17, 107.2, 100.6	
V (Å³)	325	
Mosaicity (deg.)	0.058, 0.068, 0.15	
**Dynamical refinement setup for continuous-rotation 3D ED**		
$\Delta\alpha_v$, $\Delta\alpha_o$ (deg.) #1	2.06, 0.92	
$\Delta\alpha_v$, $\Delta\alpha_o$ (deg.) #2	2.2, 0.88	
$\Delta\alpha_v$, $\Delta\alpha_o$ (deg.) #3	2.28, 0.91	
N_F, N_O, N_{OVF}, #1	72, 32, 35	
N_F, N_O, N_{OVF}, #2	25, 10, 58	
N_F, N_O, N_{OVF}, #3	50, 20, 57	
R_{Sg}^{max}, D_{Sg}^{min}, g_{max}^{BW}	0.75, 0.0015 Å^{-1}, 1.9 Å^{-1}	
**Refinement**	**dynamical**	**kinematical**
g_{max}^{ref} (Å^{-1})	1.70	1.52
$\sin(\theta_{max})/\lambda$ (Å^{-1})	0.85	0.76
d_{min} (Å)	0.59	0.66
Completeness	65.8%	98.1%
N_{obs}, N_{all}	3278, 7210	852, 2434
Parameters	205	118
R_{obs}, MR_{obs}	0.093, 0.098	0.148, 0.148
R_{all}, MR_{all}	0.159, 0.149	0.231, 0.231
wR_{all}, MwR_{all}	0.099, 0.092	0.217, 0.217
$\sigma[\Delta V(\mathbf{r})]$ (e/Å)	0.117	0.420
$\min[\Delta V(\mathbf{r})]$, $\max[\Delta V(\mathbf{r})]$	−0.436, 0.483	−1.771, 1.701
RMSD (Å)	0.014	0.021

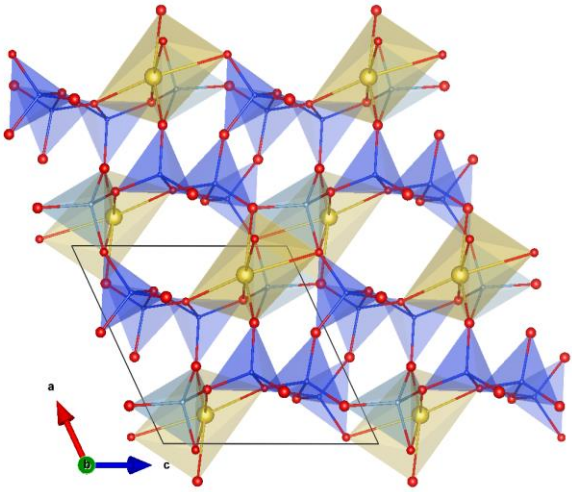

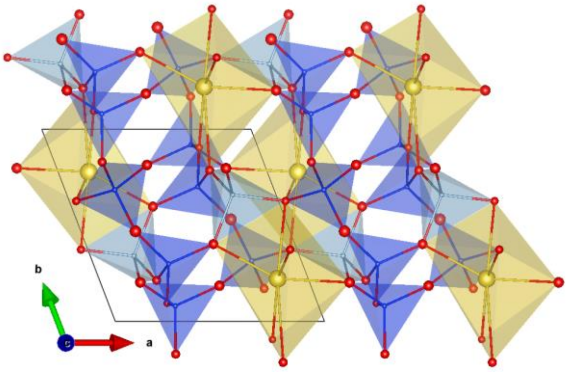

*Detector was operated at 1000 Hz, but 50 frames were merged before the data reduction step[19].

Tab. S8. Natrolite.

Relevant parameters of the measurement, data reduction and refinement results of natrolite. One crystal was measured in a quasi-simultaneous way using precession-assisted and (stepwise) continuous-rotation 3D ED so that the refinement results are directly comparable (see Methods). Reference atomic distances for the calculation of RMSD were taken from reference[66], CCDC code 417178. Atom colour codes: silicon: blue; sodium: yellow; oxygen: red; hydrogen: pink

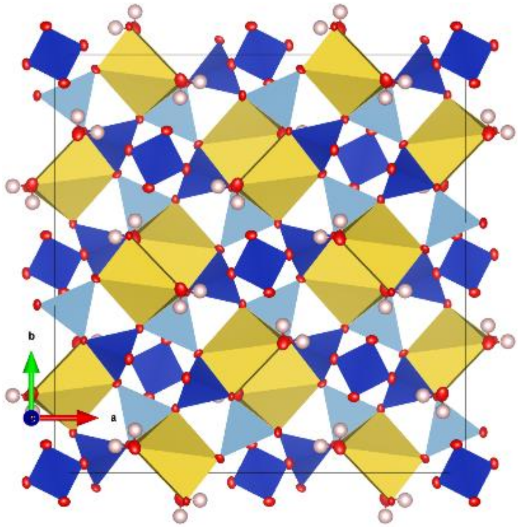

**Measurement of natrolite**				
Microscope	FEI Tecnai G2 20			
Detector (type)	Olympus SIS Veleta (CCD)			
3D ED data sets	1 continuous-rotation<br>1 precession-assisted			
λ (Å)	0.02508			
T (K)	293			
α_{min}, α_{max}, $\Delta\alpha$ (deg.)	−50.0, 50.0, 0.6			
η_{exp}, η_{gap} (deg.)	0.6, 0.0			
**Crystal**				
Empirical formula	$Na_2(Al_2Si_3O_{10})(H_2O)_2$			
Z	8			
Space group	*Fdd*2			
a, b, c (Å)	18.2682, 18.6357, 6.6142			
α, β, γ (deg.)	90, 90, 90			
V (Å^3)	2251.74			
Mosaicity (deg.)	0.18			
Completeness	98.5 %			
**Dynamical refinement setup for continuous-rotation 3D ED**			**Precession-assisted 3D ED**	
$\Delta\alpha_v$, $\Delta\alpha_o$ (deg.)	1.2, 0.6		φ (deg.)	0.54
N_F, N_O, N_{OVF}	2, 1, 161		N_{frames}	101
R_{Sg}^{max}, D_{Sg}^{min}, g_{max}^{BW}	0.75, 0.0025 Å^{-1}, 1.8 Å^{-1}		R_{Sg}^{max}, D_{Sg}^{min}, g_{max}^{BW}	0.75, 0.0025 Å^{-1}, 1.8 Å^{-1}
**Refinement**	**dynamical**	**kinematical**	**dynamical**	**kinematical**
g_{max}^{ref} (Å^{-1})	1.50	1.50	1.50	1.50
$\sin(\theta_{max})/\lambda$ (Å^{-1})	0.75	0.75	0.75	0.75
d_{min} (Å)	0.67	0.67	0.67	0.67
N_{obs}, N_{all}	2478, 5175	1135, 1979	4119, 8857	1065, 2408
Parameters	253	93	259	93
R_{obs}, MR_{obs}	0.085, 0.071	0.119, 0.119	0.075, 0.059	0.124, 0.124
R_{all}, MR_{all}	0.173, 0.119	0.176, 0.176	0.158, 0.103	0.235, 0.235
wR_{all}, MwR_{all}	0.083, 0.072	0.133, 0.133	0.084, 0.063	0.134, 0.134
$\sigma[\Delta V(\mathbf{r})]$ (e/Å)	0.067	0.170	0.061	0.186
$\min[\Delta V(\mathbf{r})]$, $\max[\Delta V(\mathbf{r})]$	−0.282, 0.236	−0.630, 0.719	−0.245, 0.219	−0.861, 0.699
RMSD (Å)	0.014	0.016	0.015	0.027
Detected H sites	2	2	2	2

Tab. S9. Mordenite.

Relevant parameters of the measurement, data reduction and refinement results of mordenite. The data reduction and refinement are based on a measurement used in a previous publication, which is described as "Data set 2" in Table 1 in reference[20]. The structure model reported therein has the CCDC code 1875577. As an independent XRD-based reference structure was not available, a reference structure was determined by geometrical optimisation using force-field calculations as implemented in GULP[67] with typical Buckingham potentials tested for a range of zeolites[68]. Atom colour codes: silicon: blue; oxygen: red

**Measurement of mordenite**		
Microscope	JEOL JEM-2100-Lab6	
Detector (type)	ASI Timepix (HPD)	
3D ED data sets	1 continuous-rotation	
λ (Å)	0.02508	
T (K)	293	
α_{min}, α_{max}, $\Delta\alpha$ (deg.)	−43.9, 58.7, 0.23	
η_{exp}, η_{gap} (deg.)	0.2281, 0.0055	
**Crystal**		
Empirical formula	SiO_2	
Z	48	
Space group	*Cmcm*	
a, b, c (Å)	18.4813, 20.1816, 7.6423	
α, β, γ (deg.)	90, 90, 90	
V (Å³)	2850.44	
Mosaicity (deg.)	0.23	
Completeness	92.1 %	
**Dynamical refinement setup**		
$\Delta\alpha_v$, $\Delta\alpha_o$ (deg.)	1.638, 0.9372	
N_F, N_O, N_{OVF}	7, 4, 82	
R_{Sg}^{max}, D_{Sg}^{min}, g_{max}^{BW}	0.6, 0.002 Å^{-1}, 1.42 Å^{-1}	
**Refinement**	**dynamical**	**kinematical**
g_{max}^{ref} (Å^{-1})	1.30	1.30
$\sin(\theta_{max})/\lambda$ (Å^{-1})	0.65	0.65
d_{min} (Å)	0.77	0.77
N_{obs}, N_{all}	3002, 7581	985, 1745
Parameters	177	96
R_{obs}, MR_{obs}	0.096, 0.089	0.173, 0.173
R_{all}, MR_{all}	0.164, 0.124	0.237, 0.237
wR_{all}, MwR_{all}	0.110, 0.105	0.207, 0.207
$\sigma[\Delta V(\mathbf{r})]$ (e/Å)	0.090	0.277
$\min[\Delta V(\mathbf{r})]$, $\max[\Delta V(\mathbf{r})]$	−0.338, 0.553	−1.092, 0.998
RMSD (Å)	0.015	0.020

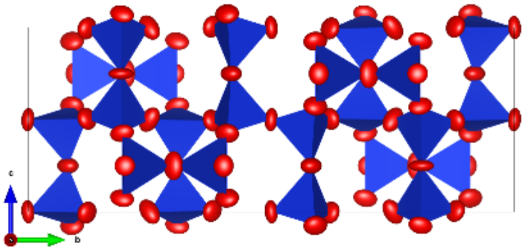

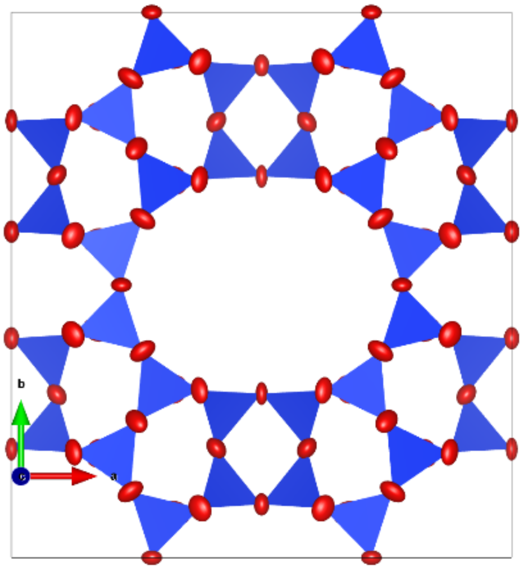

Tab. S10. STW_HPM-1 (T = 100 K).

Relevant parameters of the measurement, data reduction and refinement results of the chiral zeolite STW_HPM-1. For the calculation of RMSD only Si-O distances were considered. As a reference the refined structure from the single crystal XRD measurement was used (Tab. S18), CCDC code 2237211. Atom colour codes: silicon: blue; oxygen: red; carbon: brown; fluorine: light blue; hydrogen: pink. Hydrogen positions shown only with the pink-coloured sticks indicating the bond between the carbon and hydrogen atoms.

**Measurement of STW_HPM-1**		
Microscope	Titan Krios	
Detector (type)	CETA-D (CMOS)	
3D ED data sets	1 continuous-rotation	
λ (Å)	0.0196	
T (K)	100	
α_{min}, α_{max}, $\Delta\alpha$ (deg.)	−45.5, 66.4, 0.3	
η_{exp}, η_{gap} (deg.)	0.3, 0.0	
**Crystal**		
Empirical formula	$(C_8N_2H_{15})F[Si_{10}O_{20}]$	
Z	6	
Space group	$P6_122$	
a, b, c (Å)	12.0226, 12.0226, 29.7711	
α, β, γ (deg.)	90, 90, 120	
V (Å³)	3726.7	
Mosaicity (deg.)	0.0	
Completeness	100.0 %	
**Dynamical refinement setup**		
$\Delta\alpha_v$, $\Delta\alpha_o$ (deg.)	2.1, 0.9	
N_F, N_O, N_{OVF}	7, 3, 92	
R_{Sg}^{max}, D_{Sg}^{min}, g_{max}^{BW}	0.7, 0.0015 Å^{-1}, 1.35 Å^{-1}	
**Refinement**	**dynamical**	**kinematical**
g_{max}^{ref} (Å^{-1})	1.35	1.40
$\sin(\theta_{max})/\lambda$ (Å^{-1})	0.675	0.7
d_{min} (Å)	0.74	0.71
N_{obs}, N_{all}	20995, 28067	1665, 3478
Parameters	280	81
R_{obs}, MR_{obs}	0.109, 0.064	0.158, 0.158
R_{all}, MR_{all}	0.125, 0.064	0.227, 0.227
wR_{all}, MwR_{all}	0.122, 0.077	0.231, 0.231
$\sigma[\Delta V(\mathbf{r})]$ (e/Å)	0.113	0.311
$\min[\Delta V(\mathbf{r})]$, $\max[\Delta V(\mathbf{r})]$	−0.445, 0.742	−1.345, 1.479
RMSD (Å)	0.006	0.019

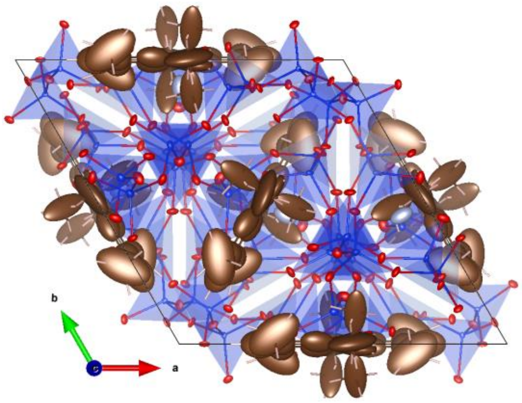

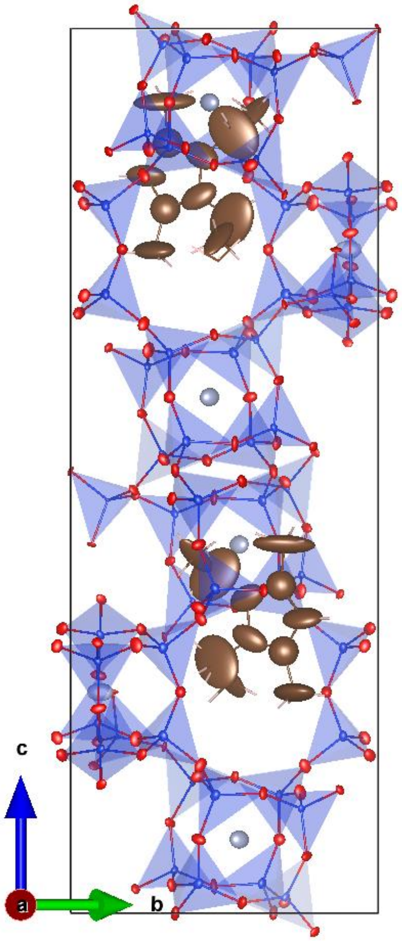

Tab. S11. CAP.

Relevant parameters of the measurement, data reduction and refinement results of CAP. Two crystals were measured with a simplified, static experimental setup. The beam and goniometer were not moving during the exposure of the diffraction patterns. After each frame, the goniometer was tilted by 0.10°. This setup is similar to the original ADT (automated diffraction tomography) data acquisition with finer tilt steps, but without operating in scanning TEM mode. XRD-based reference atomic distances for the calculation of RMSD were taken from[35], CCDC code 1864822. Atom colour codes: cobalt: blue polyhedra; aluminium: blue-grey octahedra; phosphorus: pink tetrahedra; oxygen: red; hydrogen: light pink.

**Measurement of CAP**		
Microscope	FEI Tecnai G2 20	
Detector (type)	Olympus SIS Veleta (CCD)	
3D ED data sets	3 static (from 2 crystals)	
λ (Å)	0.02508	
T (K)	100	
α_{min}, α_{max}, $\Delta\alpha$ (deg.) #1	−45.0, 57.8, 0.1	
α_{min}, α_{max}, $\Delta\alpha$ (deg.) #2	−41.4, −10.7, 0.1	
α_{min}, α_{max}, $\Delta\alpha$ (deg.) #3	−9.2, 22.8, 0.1	
η_{exp}, η_{gap} (deg.)	0, 0.1	
**Crystal**		
Empirical formula	$Co_{1.14}Al_2P_4O_{20}H_{11.72}$	
Z	2	
Space group	$P2_1/n$	
a, b, c (Å)	8.4759, 16.6415, 5.0478	
α, β, γ (deg.)	90, 90.63, 90	
V (Å^3)	711.96	
Mosaicity (deg.)	0.10	
Completeness	90.0 %	
**Dynamical refinement setup**		
$\Delta\alpha_v$, $\Delta\alpha_o$ (deg.)	1.5, 0.8	
N_F, N_O, N_{OVF}	15, 8, 146 + 45 + 46	
R_{Sg}^{max}, D_{Sg}^{min}, g_{max}^{BW}	0.6, 0.0025 Å^{-1}, 1.6 Å^{-1}	
**Refinement**	**dynamical**	**kinematical**
g_{max}^{ref} (Å^{-1})	1.4	1.4
$\sin(\theta_{max})/\lambda$ (Å^{-1})	0.7	0.7
d_{min} (Å)	0.71	0.71
N_{obs}, N_{all}	5934, 8889	1111, 1864
Parameters	391	153
R_{obs}, MR_{obs}	0.111, 0.091	0.165, 0.165
R_{all}, MR_{all}	0.141, 0.102	0.231, 0.231
wR_{all}, MwR_{all}	0.118, 0.101	0.176, 0.176
$\sigma[\Delta V(\mathbf{r})]$ (e/Å)	0.125	0.278
$\min[\Delta V(\mathbf{r})]$, $\max[\Delta V(\mathbf{r})]$	−0.455, 0.692	−0.985, 0.911
RMSD (Å)	0.022	0.032

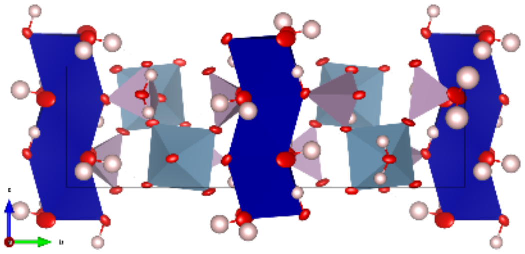

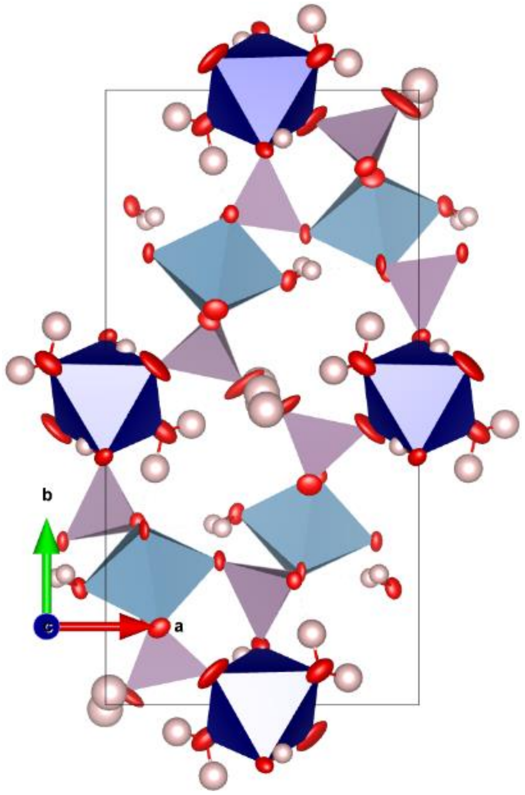

Tab. S12. CAU-36.

Relevant parameters of the measurement, data reduction and refinement results of CAU-36. XRD-based reference atomic distances for the calculation of RMSD were determined using only intermolecular distances involving C and N atoms using the program MOGUL (Molecular geometry library)[69]. A reference structure model based on an independent single crystal XRD measurement was not available, but the model from a previously reported kinematical refinement against 3D ED data has the CCDC code 1865991. Atom colour codes: cobalt: blue; nickel: grey; phosphorus: purple; oxygen: red; carbon: brown; nitrogen: light blue; hydrogen: pink.

**Measurement of CAU-36**		
Microscope	JEOL JEM-2100-Lab6	
Detector (type)	ASI Timepix (HPD)	
3D ED data sets	4 continuous-rotation	
λ (Å)	0.02508	
T (K)	100	
α_{min}, α_{max}, $\Delta\alpha$ (deg.) #1	−43.8, 45.5, 0.23	
α_{min}, α_{max}, $\Delta\alpha$ (deg.) #2	−44.9, 56.2, 0.23	
α_{min}, α_{max}, $\Delta\alpha$ (deg.) #3	−48.5, 38.8, 0.23	
α_{min}, α_{max}, $\Delta\alpha$ (deg.) #4	−48.4, 60.5, 0.23	
η_{exp}, η_{gap} (deg.)	0.23, 0.023	
**Crystal**		
Empirical formula	$C_{56}H_{48}Co_2N_8NiO_{18}P_4$	
Z	8	
Space group	$P{-}4c2$	
a, b, c (Å)	21.6568, 21.6568, 8.9863	
α, β, γ (deg.)	90, 90, 90	
V (Å^3)	4214.7	
Mosaicity #1-#4 (deg.)	0.23, 0.21, 0.09, 0.06	
Completeness	94.2%	
**Dynamical refinement setup**		
$\Delta\alpha_v$, $\Delta\alpha_o$ (deg.)	1.84, 0.69	
N_F, N_O, N_{OVF}	8, 3, 33+11+30+32	
R_{Sg}^{max}, D_{Sg}^{min}, g_{max}^{BW}	0.6, 0.0015 Å^{-1}, 1.05 Å^{-1}	
**Refinement**	**dynamical**	**kinematical**
g_{max}^{ref} (Å^{-1})	1.0	1.0
$\sin(\theta_{max})/\lambda$ (Å^{-1})	0.5	0.5
d_{min} (Å)	1.0	1.0
N_{obs}, N_{all}	4339, 8731	1352, 2087
Parameters	214	107
R_{obs}, MR_{obs}	0.122, 0.106	0.141, 0.141
R_{all}, MR_{all}	0.186, 0.133	0.175, 0.175
wR_{all}, MwR_{all}	0.127, 0.106	0.155, 0.155
$\sigma[\Delta V(\mathbf{r})]$ (e/Å)	0.084	0.134
$\min[\Delta V(\mathbf{r})]$, $\max[\Delta V(\mathbf{r})]$	−0.362, 0.364	−0.454, 0.555
RMSD (Å)	0.020	0.034

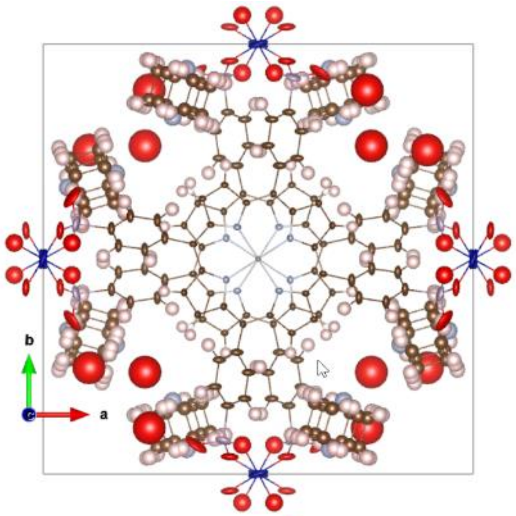

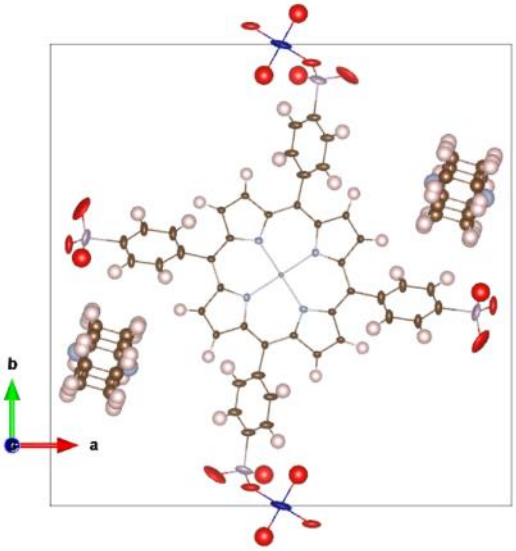

Tab. S13. α-glycine.

Relevant parameters of the measurement, data reduction and refinement results of α-glycine. The data reduction and refinement are based on a measurement used in previous publication, which is described as "Data set 7" in Table S2 in reference [21]. Reference atomic distances for the calculation of RMSD were taken from reference [63], CCDC code 849663. Atom colour codes: carbon: brown; nitrogen: light blue; oxygen: red; hydrogen: pink.

**Measurement of α-glycine**		
Microscope	JEOL JEM-2100-Lab6	
Detector (type)	ASI Timepix (HPD)	
3D ED data sets	1 continuous-rotation	
λ (Å)	0.02508	
T (K)	100	
α_{min}, α_{max}, $\Delta\alpha$ (deg.)	−11.62, 46.73, 0.36	
η_{exp}, η_{gap} (deg.)	0.3421, 0.0137	
**Crystal**		
Empirical formula	$C_2H_5NO_2$	
Z	4	
Space group	$P2_1/n$	
a, b, c (Å)	5.0893, 11.8124, 5.4668	
α, β, γ (deg.)	90, 112.0058, 90	
V (Å^3)	304.7	
Mosaicity (deg.)	0.20	
Completeness	40.0 %	
**Dynamical refinement setup**		
$\Delta\alpha_v$, $\Delta\alpha_o$ (deg.)	1.8, 1.08	
N_F, N_O, N_{OVF}	5, 3, 60	
R_{Sg}^{max}, D_{Sg}^{min}, g_{max}^{BW}	0.7, 0.0015 Å^{-1}, 1.7 Å^{-1}	
**Refinement**	**dynamical**	**kinematical**
g_{max}^{ref} (Å^{-1})	1.70	1.70
$\sin(\theta_{max})/\lambda$ (Å^{-1})	0.85	0.85
d_{min} (Å)	0.59	0.59
N_{obs}, N_{all}	1247, 1540	474, 619
Parameters	126	67
R_{obs}, MR_{obs}	0.068, 0.067	0.136, 0.136
R_{all}, MR_{all}	0.076, 0.073	0.161, 0.161
wR_{all}, MwR_{all}	0.088, 0.092	0.159, 0.159
$\sigma[\Delta V(\mathbf{r})]$ (e/Å)	0.048	0.131
$\min[\Delta V(\mathbf{r})]$, $\max[\Delta V(\mathbf{r})]$	−0.162, 0.197	−0.420, 0.412
RMSD (Å)	0.004	0.005

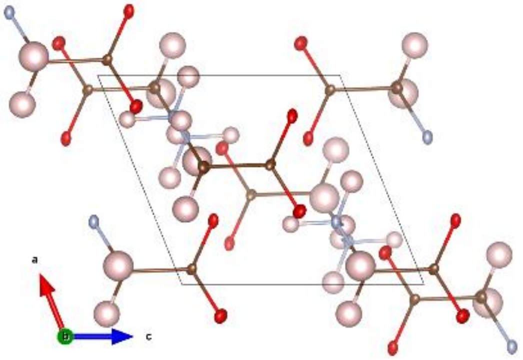

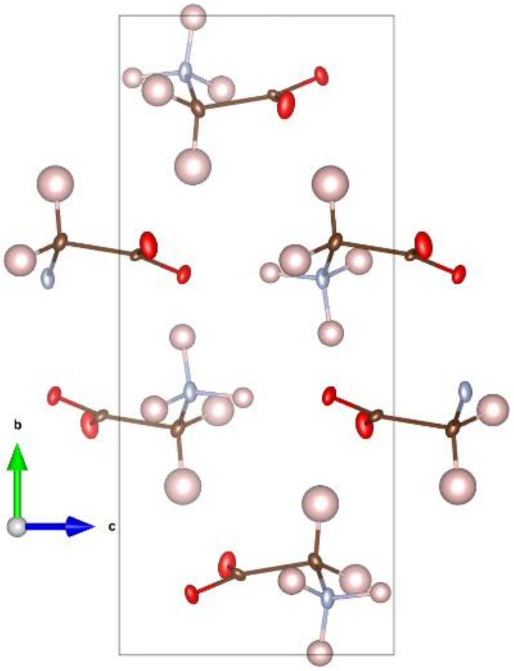

Tab. S14. Carbamazepine.

Relevant parameters of the measurement, data reduction and refinement results of carbamazepine. The data reduction and refinement are based on a subset of measurements used in a publication from 2018[22]. Reference atomic distances for the calculation of RMSD were taken from reference[70], CCDC code 1027345. Atom colour codes: carbon: brown; nitrogen: light blue; oxygen: red; hydrogen: pink.

**Measurement of carbamazepine**		
Microscope	Thermo Fisher Talos Arctica	
Detector (type)	Thermo Fisher Ceta-D (CMOS)	
3D ED data sets	2 continuous-rotation (from 2 crystals)	
λ (Å)	0.02508	
T (K)	100	
α_{min}, α_{max}, $\Delta\alpha$ (deg.) #1	−59.7, 28.2, 0.92	
η_{exp}, η_{gap} (deg.) #1	0.92, 0	
α_{min}, α_{max}, $\Delta\alpha$ (deg.) #2	−38.9, 48.1, 0.62	
η_{exp}, η_{gap} (deg.) #2	0.62, 0	
**Crystal**		
Empirical formula	$C_{15}H_{12}N_2O$	
Z	4	
Space group	$P2_1/n$	
a, b, c (Å)	7.5162, 11.0697, 13.7782	
α, β, γ (deg.)	90, 93.0492, 90	
V (Å³)	1144.75	
Mosaicity #1, #2 (deg.)	0.21, 0.018	
Completeness	77.5 %	
**Dynamical refinement setup**		
$\Delta\alpha_v$, $\Delta\alpha_o$ (deg.) #1	2.76, 1.84	
$\Delta\alpha_v$, $\Delta\alpha_o$ (deg.) #2	1.89, 1.26	
N_F, N_O, N_{OVF}	3, 2, 91 + 140	
R_{Sg}^{max}, D_{Sg}^{min}, g_{max}^{BW}	0.6, 0.002 Å^{-1}, 1.5 Å^{-1}	
**Refinement**	**dynamical**	**kinematical**
g_{max}^{ref} (Å^{-1})	1.28	1.28
sin(θ_{max})/λ (Å^{-1})	0.64	0.64
d_{min} (Å)	0.78	0.78
N_{obs}, N_{all}	7754, 11583	1128, 1978
Parameters	407	176
R_{obs}, MR_{obs}	0.124, 0.100	0.164, 0.164
R_{all}, MR_{all}	0.153, 0.109	0.227, 0.227
wR_{all}, MwR_{all}	0.131, 0.112	0.187, 0.187
$\sigma[\Delta V(\mathbf{r})]$ (e/Å)	0.113	0.162
min[$\Delta V(\mathbf{r})$], max[$\Delta V(\mathbf{r})$]	−0.377, 0.431	−0.753, 0.557
RMSD (Å)	0.012	0.033

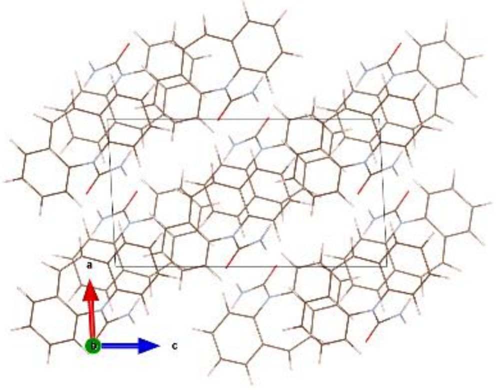

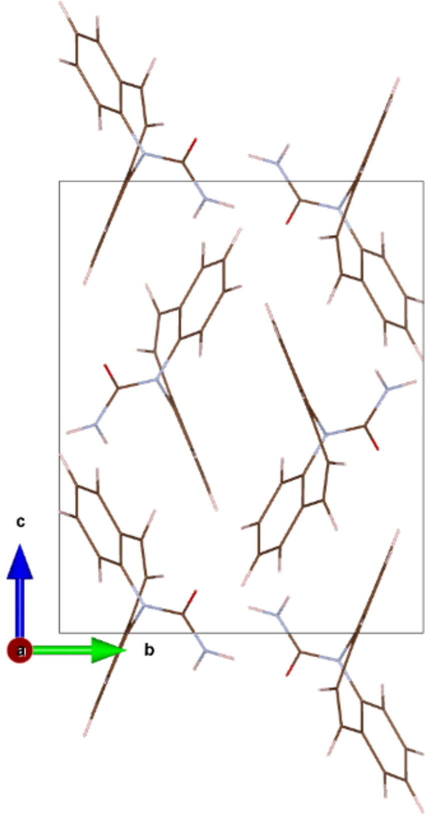

Tab. S15. (+)-limaspermidine.

Relevant parameters of the measurement, data reduction and refinement results of (+)-limaspermidine. The data reduction and refinement are based on a subset of measurements used in a publication from 2018[22]. Reference atomic distances for the calculation of RMSD were taken from reference[71], CCDC code 1049103. Note that the reference structure was measured at room temperature, whereas the data sets used in this study were measured at T = 100 K. Atom colour codes: carbon: brown; nitrogen: light blue; oxygen: red; hydrogen: pink.

**Measurement of (+)-limaspermidine**		
Microscope	Thermo Fisher Talos Arctica	
Detector (type)	Thermo Fisher Ceta-D (CMOS)	
3D ED data sets	2 continuous-rotation (from 1 crystal)	
λ (Å)	0.02508	
T (K)	100	
α_{min}, α_{max}, $\Delta\alpha$ (deg.) #1	−55.9, −2.8, 0.92	
α_{min}, α_{max}, $\Delta\alpha$ (deg.) #2	2.6, 57.4, 0.92	
η_{exp}, η_{gap} (deg.)	0.92, 0.0	
**Crystal**		
Empirical formula	$C_{19}H_{26}N_2O$	
Z	4	
Space group	$P2_12_12_1$	
a, b, c (Å)	7.6339, 13.7753, 15.1617	
α, β, γ (deg.)	90, 90, 90	
V (Å^3)	1594.39	
Mosaicity #1, #2 (deg.)	0.01, 0.02	
Completeness	97.0 %	
**Dynamical refinement setup**		
$\Delta\alpha_v$, $\Delta\alpha_o$ (deg.)	2.7, 1.8	
N_F, N_O, N_{OVF}	3, 2, 59 + 57	
R_{Sg}^{max}, D_{Sg}^{min}, g_{max}^{BW}	0.6, 0.002 Å^{-1}, 1.25 Å^{-1}	
**Refinement**	**dynamical**	**kinematical**
g_{max}^{ref} (Å^{-1})	1.2	1.2
$\sin(\theta_{max})/\lambda$ (Å^{-1})	0.6	0.6
d_{min} (Å)	0.83	0.83
N_{obs}, N_{all}	7745, 11683	1567, 2861
Parameters	212	96
R_{obs}, MR_{obs}	0.140, 0.114	0.142, 0.142
R_{all}, MR_{all}	0.210, 0.159	0.220, 0.220
wR_{all}, MwR_{all}	0.141, 0.116	0.160, 0.160
$\sigma[\Delta V(\mathbf{r})]$ (e/Å)	0.123	0.176
min[$\Delta V(\mathbf{r})$], max[$\Delta V(\mathbf{r})$]	−0.500, 0.494	−0.702, 0.700
RMSD (Å)	0.026	0.047

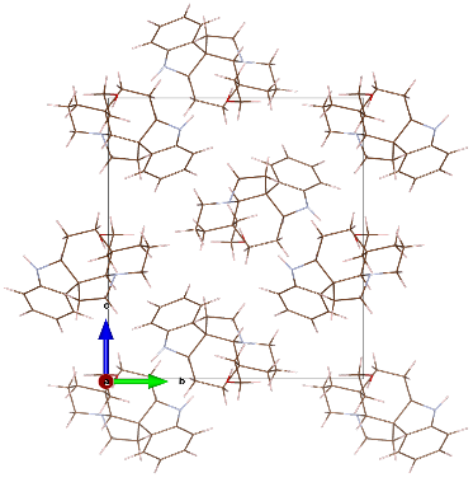

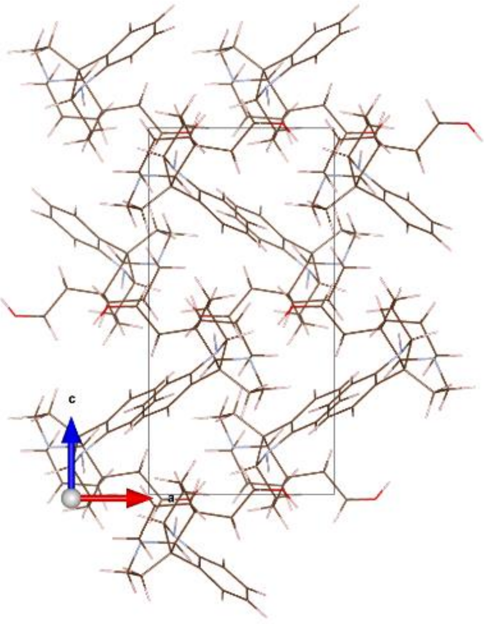

Tab. S16. Abiraterone acetate.

Relevant parameters of the measurement, data reduction and refinement results of abiraterone acetate. Reference atomic distances for the calculation of RMSD were taken from reference[72], CCDC code 2035079. Atom colour codes: carbon: brown; nitrogen: light blue; oxygen: red; hydrogen: pink.

**Measurement of abiraterone acetate**		
Microscope	FEI Tecnai G2 20	
Detector (type)	Olympus SIS Veleta (CCD)	
3D ED data sets	5 continuous-rotation (from 5 crystals)	
λ (Å)	0.02508	
T (K)	100	
α_{min}, α_{max}, $\Delta\alpha$ (deg.) #1	−28.3, 51.8, 0.4	
α_{min}, α_{max}, $\Delta\alpha$ (deg.) #2	−38.6, 48.7, 0.4	
α_{min}, α_{max}, $\Delta\alpha$ (deg.) #3	−30.0, 48.6, 0.4	
α_{min}, α_{max}, $\Delta\alpha$ (deg.) #4	−11.3, 39.4, 0.4	
α_{min}, α_{max}, $\Delta\alpha$ (deg.) #5	−29.0, 25.1, 0.4	
η_{exp}, η_{gap} (deg.)	0.4, 0.0	
**Crystal**		
Empirical formula	$C_{26}H_{33}NO_2$	
Z	4	
Space group	$P2_12_12_1$	
a, b, c (Å)	7.4703, 9.6887, 30.2032	
α, β, γ (deg.)	90, 90, 90	
V (Å^3)	2186.03	
Mosaicity #1−#5 (deg.)	0.16, 0.018, 0.013, 0.13, 0.05	
Completeness	91.7 %	
**Dynamical refinement setup**		
$\Delta\alpha_v$, $\Delta\alpha_o$ (deg.) #1−#3	2.0, 1.2	
$\Delta\alpha_v$, $\Delta\alpha_o$ (deg.) #4, #5	2.0, 0.8	
N_F, N_O, N_{OVF}, #1−#3	5, 3, 35 + 61 + 77	
N_F, N_O, N_{OVF}, #4, #5	5, 2, 33 + 41	
R_{Sg}^{max}, D_{Sg}^{min}, g_{max}^{BW}	0.5, 0.0025 Å^{-1}, 1.0 Å^{-1}	
**Refinement**	**dynamical**	**kinematical**
g_{max}^{ref} (Å^{-1})	0.96	0.96
$\sin(\theta_{max})/\lambda$ (Å^{-1})	0.48	0.48
d_{min} (Å)	1.04	1.04
N_{obs}, N_{all}	4839, 10960	932, 1902
Parameters	370	118
R_{obs}, MR_{obs}	0.112, 0.095	0.163, 0.163
R_{all}, MR_{all}	0.208, 0.169	0.247, 0.247
wR_{all}, MwR_{all}	0.118, 0.089	0.188, 0.188
$\sigma[\Delta V(\mathbf{r})]$ (e/Å)	0.099	0.175
min[$\Delta V(\mathbf{r})$], max[$\Delta V(\mathbf{r})$]	−0.563, 0.459	−0.715, 0.748
RMSD (Å)	0.049	0.071

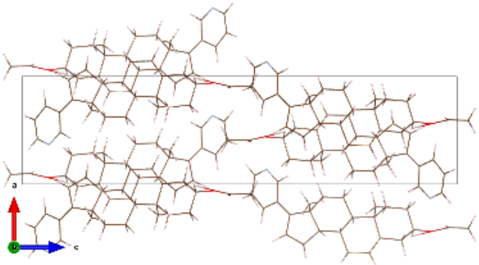

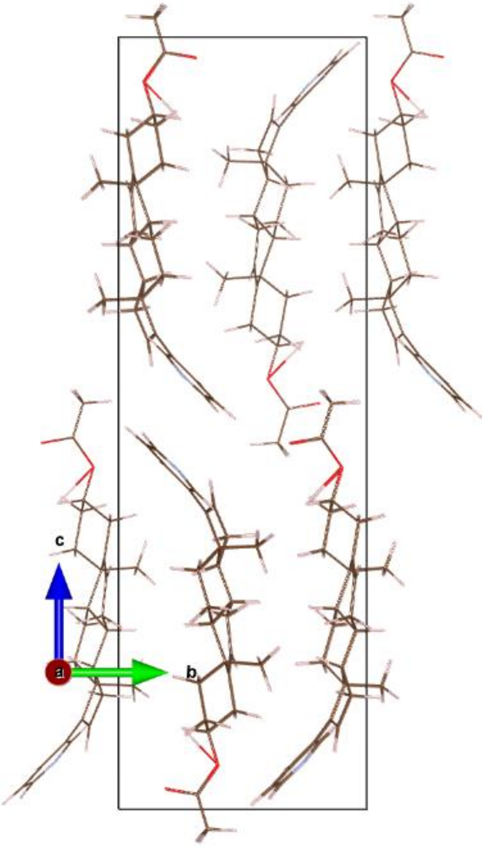

Tab. S17. MBBF4.

Data reduction and refinement based on a subset of measurements used in a publication from 2018 [23]. Used data sets (T = 298 K) are labelled as 9, 10, 11, 12, 13, 14, 17 and 21 in Table S4 in reference[72]. XRD-based reference (T = 80 K) atomic distances for the calculation of RMSD were taken from reference[72], CCDC code 1856585. Atom colour codes: carbon: brown; nitrogen: light blue; oxygen: red; fluorine: dark green; boron: green; hydrogen: pink.

**Measurement of MBBF4**		
Microscope	FEI Tecnai F30	
Detector (type)	DECTRIS EIGER X 1M (HPD)	
3D ED data sets	8 continuous-rotation (from 1 crystal)	
λ (Å)	0.02508	
T (K)	293	
α_{min}, α_{max} (deg.)	−60.0, 72.3	
$\Delta\alpha$ (deg.) #1−#4	0.1435	
$\Delta\alpha$ (deg.) #5−#8	0.0293	
η_{exp}, η_{gap} (deg.) #1−#4	0.1435, < 0.00001	
η_{exp}, η_{gap} (deg.) #5−#8	0.0293, < 0.00001	
**Crystal**		
Empirical formula	$C_{90}H_{91}B_4F_{16}N_{21}S_3$	
Z	4	
Space group	$C2/c$	
a, b, c (Å)	39.8699, 16.5497, 13.7728	
α, β, γ (deg.)	90, 99.114, 90	
V (Å³)	8973.03	
Mosaicity (deg.) #1−#8	0.05, 0.05, 0.05, 0.065, 0.09, 0.09, 0.09, 0.06	
Completeness	80.6 %	
**Dynamical refinement setup**		
$\Delta\alpha_v$, $\Delta\alpha_o$ (deg.) #1−#4	1.14, 0.43	
$\Delta\alpha_v$, $\Delta\alpha_o$ (deg.) #5−#8	1.47, 0.29	
N_F, N_O, N_{OVF} #1−#4	10, 3, 21 + 30 + 22 + 40	
N_F, N_O, N_{OVF} #5−#8	50, 10, 45 + 44 + 19 + 24	
R_{Sg}^{max}, D_{Sg}^{min}, g_{max}^{BW}	0.7, 0.003 Å^{-1}, 1.12 Å^{-1}	
**Refinement**	**dynamical**	**kinematical**
g_{max}^{ref} (Å^{-1})	1.0	1.0
$\sin(\theta_{max})/\lambda$ (Å^{-1})	0.5	0.5
d_{min} (Å)	1.0	1.0
N_{obs}, N_{all}	6820, 14629	1979, 3934
Parameters	537	277
R_{obs}, MR_{obs}	0.159, 0.146	0.294, 0.294
R_{all}, MR_{all}	0.247, 0.183	0.358, 0.358
wR_{all}, MwR_{all}	0.153, 0.133	0.332, 0.332
$\sigma[\Delta V(\mathbf{r})]$ (e/Å)	0.093	0.372
$\min[\Delta V(\mathbf{r})]$, $\max[\Delta V(\mathbf{r})]$	−0.428, 0.469	−1.689, 1.759
RMSD (Å)	0.052	0.075

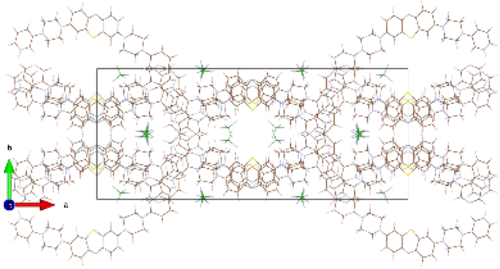

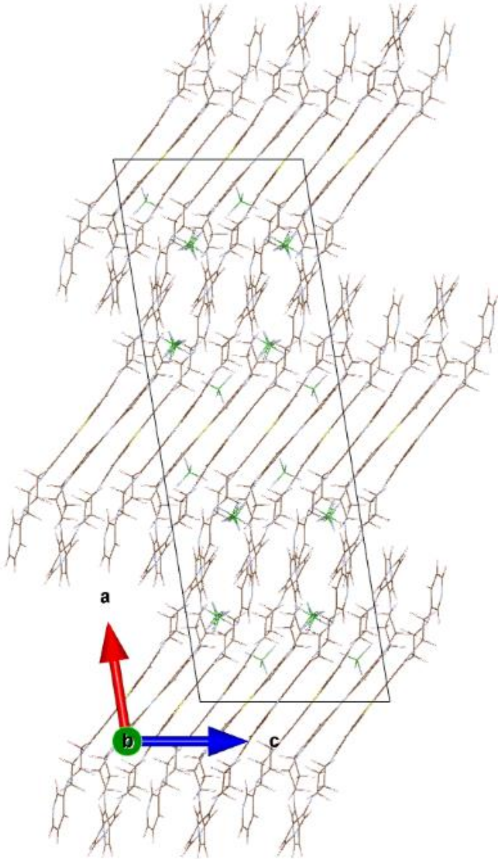

Tab. S18. Absolute structure of STW_HPM-1 by XRD.
Crystal data, details of data collection with single crystal X-ray diffraction and structure refinement of STW_HPM-1. The crystal used for the data collection was crushed after the measurement and prepared for 3D ED measurements. The refined model was deposited at the CCDC with deposition number 2237211.

**Crystal Data**	
Formula	$\|(C_8N_2H_{15})F\|[Si_{10}O_{20}]$
Formula Weight (g/mol)	3604.8
Crystal System	Hexagonal
Space group	$P6_522$
a, b, c (Å)	11.8891(2), 11.8891(2), 29.6735(5)
α, β, γ (°)	90, 90, 120
Volume, Å^3	3632.43(14)
**Data Collection**	
Temperature (K)	296
Radiation, wavelength (Å)	Cu K_α, 1.54178
g_{max}^{ref} (Å^{-1})	1.25
$\sin(\theta_{max})/\lambda$ (Å^{-1})	0.625
d_{min}, d_{max} (Å)	0.8, 10.30
Dataset (h, k, l)	−14→14, −14→14, −37→37
Tot., Uniq. Data, R_{int}	50870, 2484, 0.1134
Completeness (%)	100
$<I/\sigma(I)>$	29.7
R_{meas} (%)	0.117
$CC_{1/2}$	0.999
**Refinement**	
$N_{reflections}$, R_1, wR_2 ($I > 2\sigma(I)$)	2315, 0.0330, 0.0762
$N_{reflections}$, R_1, wR_2 (all data)	2484, 0.0376, 0.0778
$N_{parameters}$, $N_{restraints}$	194, 0
GooF	1.111
Flack parameter	0.06(2)

Tab. S19. Absolute structure of SWT_HPM-1 by dynamical refinement.

Relevant parameters of the measurement, data reduction and refinement results of the chiral zeolite STW_HPM-1 collected at room temperature, used for the absolute structure determination blind test. The structure was refined without modelling the OSDA molecule.

**Absolute structure determination of STW_HPM-1**		
Microscope	JEOL JEM-2100-LaB6	
Detector (type)	ASI Timepix	
3D ED data sets	1 continuous-rotation	
Radiation, λ (Å)	electrons, 0.02508	
T (K)	293	
**Crystal**		
Empirical formula	$Si_{10}O_{20}$	
Z	6	
a, b, c (Å)	11.8945, 11.8945, 29.67	
α, β, γ (deg.)	90, 90, 120	
V (Å^3)	3635.3	
Completeness	100.0 %	
**Dynamical refinement setup**		
$\Delta\alpha_v$, $\Delta\alpha_o$ (deg.)	3.44, 0.9	
N_F, N_O, N_{OVF}	7, 3, 92	
R_{Sg}^{max}, D_{Sg}^{min}, g_{max}^{BW}	0.4, 0.0015 Å^{-1}, 1.35 Å^{-1}	
**Dynamical refinement**	**enantiomorph #1**	**enantiomorph #2**
g_{max}^{ref} (Å^{-1})	1.35	
$\sin(\theta_{max})/\lambda$ (Å^{-1})	0.675	
d_{min} (Å)	0.74	
N_{obs}, N_{all}	9732, 21832	
Parameters	231	
space group	$P6_522$	$P6_122$
R_{obs}	0.171	0.235
R_{all}	0.227	0.294
wR_{all}	0.234	0.307
z-score	38.3σ	−38.3σ

Tab. S20. **Absolute structure determinations.**
All data sets were recorded at low temperature between 80 K and 100 K. Refinement statistics are based on refinements against single data sets from individual crystals. Enantiomorph #1 corresponds to the correct absolute structure, enantiomorph #2 to the wrong absolute structure. *z*-score and *p* are the certainty and probability, respectively, that the absolute structure was correctly assigned. R_{Sg} in all cases set to 0.6, D_{Sg} set to 0.0015 Å^{-1}.

Sample	Biotin_i	Biotin_ii
Data set	provided by H. Yanagisawa *et al.*	provided by J. F. Bruhn & A. Cheng
Raw data DOI	10.5281/zenodo.3366892	10.5281/zenodo.4737864
Composition	$C_{10}H_{16}N_2O_3S$	$C_{10}H_{16}N_2O_3S$
Space group	$P2_12_12_1$	$P2_12_12_1$
Absolute structure reference	CCDC code: 1111310	CCDC code: 1111310

Author label	2044	2130	2143	2149	2154	801406	801574	802003	810542
Data set #	1	2	3	4	5	1	2	3	4
d_{min} (Å)	0.83	0.83	0.83	0.83	0.83	0.83	0.83	0.83	0.83
$\sin(\theta_{max})/\lambda$ (Å^{-1})	0.6	0.6	0.6	0.6	0.6	0.6	0.6	0.6	0.6
Completeness	42%	73%	54%	78%	60%	88%	85%	72%	59%
N_{obs}	597	862	1220	912	694	2664	1647	3099	2386
N_{all}	1059	3013	3243	2303	1739	4052	3349	4497	4169
$N_{parameters}$	88	108	106	105	92	193	172	204	194
R_{obs} (enant. #1)	0.140	0.115	0.126	0.147	0.131	0.163	0.163	0.131	0.161
R_{all} (enant. #1)	0.252	0.239	0.254	0.277	0.243	0.234	0.323	0.200	0.263
wR_{all} (enant. #1)	0.149	0.149	0.133	0.150	0.126	0.165	0.158	0.137	0.163
R_{obs} (enant. #2)	0.148	0.131	0.135	0.161	0.144	0.176	0.189	0.158	0.172
R_{all} (enant. #2)	0.268	0.252	0.264	0.290	0.262	0.249	0.348	0.228	0.281
wR_{all} (enant. #2)	0.150	0.160	0.140	0.163	0.139	0.180	0.186	0.170	0.173
z-score	3.2σ	2.9σ	2.0σ	1.4σ	4.7σ	3.1σ	4.8σ	7.9σ	5.9σ
P	99.9%	99.8%	97.6%	91.9%	100.0%	99.9%	100.0%	100.0%	100.0%

Tab. S20 cont'd. Absolute structure determinations.

Sample	Biotin_iii
Data set	provided by E. Thompson & H. T. Jenkins
Raw data DOI	10.5281/zenodo.4895412
Composition	$C_{10}H_{16}N_2O_3S$
Space group	$P2_12_12_1$
Absolute structure reference	CCDC code: 1111310

Author label	1	2	3	4	5	6
Data set #	1	2	3	4	5	6
d_{min} (Å)	0.83	0.83	0.83	0.83	0.83	0.83
$\sin(\theta_{max})/\lambda$ (Å^{-1})	0.6	0.6	0.6	0.6	0.6	0.6
Completeness	77%	65%	82%	83%	64%	85%
N_{obs}	3780	2232	4538	3576	3293	4473
N_{all}	4788	3089	5776	4652	4718	5186
$N_{parameters}$	163	133	183	163	163	173
R_{obs} (enant. #1)	0.173	0.163	0.146	0.165	0.148	0.155
R_{all} (enant. #1)	0.201	0.230	0.180	0.201	0.195	0.173
wR_{all} (enant. #1)	0.191	0.166	0.159	0.172	0.146	0.174
R_{obs} (enant. #2)	0.185	0.175	0.174	0.176	0.177	0.165
R_{all} (enant. #2)	0.216	0.239	0.211	0.214	0.225	0.183
wR_{all} (enant. #2)	0.206	0.181	0.196	0.187	0.184	0.188
z-score	3.3σ	2.2σ	9.9σ	2.4σ	10.0σ	4.0σ
p	100%	98.6%	100%	99.1%	100%	100%

Tab. S20 cont'd. Absolute structure determinations.

Sample	Biotin_iii
Data set	provided by E. Thompson & H. T. Jenkins
Raw data DOI	10.5281/zenodo.4895412
Composition	$C_{10}H_{16}N_2O_3S$
Space group	$P2_12_12_1$
Absolute structure reference	CCDC code: 1111310

Author label	7	8	9	10	11
Data set #	7	8	9	10	11
d_{min} (Å)	0.83	0.83	0.83	0.83	0.83
$\sin(\theta_{max})/\lambda$ (Å^{-1})	0.6	0.6	0.6	0.6	0.6
Completeness	68%	66%	68%	82%	62%
N_{obs}	4493	2779	4195	3389	3321
N_{all}	6412	4148	5178	4687	4654
$N_{parameters}$	193	153	173	163	163
R_{obs} (enant. #1)	0.134	0.180	0.144	0.171	0.158
R_{all} (enant. #1)	0.182	0.241	0.173	0.222	0.213
wR_{all} (enant. #1)	0.140	0.183	0.155	0.177	0.168
R_{obs} (enant. #2)	0.159	0.193	0.169	0.182	0.175
R_{all} (enant. #2)	0.206	0.255	0.197	0.236	0.231
wR_{all} (enant. #2)	0.172	0.202	0.185	0.198	0.188
z-score	9.6σ	2.2σ	8.2σ	2.6σ	3.7σ
p	100%	98.6%	100%	99.5%	100%

Tab. S20 cont'd. **Absolute structure determinations.**

Sample	Progesterone				Epicorazine A			
Data set	provided by J. F. Bruhn & A. Cheng				provided by M. T. B. Clabbers *et al.*			
Raw data DOI	10.5281/zenodo.3905397				10.5281/zenodo.1407682 (EPICZA)			
Composition	$C_{21}H_{30}O_2$				$C_{18}H_{16}N_2O_6S_2$			
Space group	$P2_12_12_1$				$P2_12_12_1$			
Absolute structure reference	CCDC code: 1238097				CCDC code: 1149960			

Author label	352373	352379	352382	352389	n15_a002	n15_a003	n15_a004	n15_a005
Data set #	1	2	3	4	1	2	3	4
d_{min} (Å)	1.06	1.11	1.0	1.18	1.11	0.77	0.91	1.0
$\sin(\theta_{max})/\lambda$ (Å^{-1})	0.47	0.45	0.5	0.425	0.45	0.65	0.55	0.5
Completeness	62%	86%	95%	78%	49.2%	72.8%	75.7%	59.2%
N_{obs}	1783	1943	3069	1480	698	2955	1652	1313
N_{all}	3211	3634	4489	2516	1020	3455	2681	2097
$N_{parameters}$	185	213	205	192	139	158	154	153
R_{obs} (enant. #1)	0.137	0.173	0.136	0.140	0.099	0.116	0.143	0.152
R_{all} (enant. #1)	0.261	0.335	0.201	0.235	0.130	0.124	0.180	0.194
wR_{all} (enant. #1)	0.139	0.185	0.146	0.148	0.102	0.128	0.137	0.137
R_{obs} (enant. #2)	0.152	0.182	0.148	0.149	0.112	0.171	0.162	0.164
R_{all} (enant. #2)	0.278	0.350	0.216	0.246	0.140	0.178	0.199	0.204
wR_{all} (enant. #2)	0.159	0.195	0.158	0.157	0.110	0.204	0.159	0.147
z-score	3.0σ	3.6σ	5.4σ	2.7σ	1.4σ	15.3σ	4.3σ	3.3σ
p	99.9%	100.0%	100.0%	100.0%	92.2%	100.0%	100.0%	99.9%

Tab. S20 cont'd. **Absolute structure determinations.**

Sample	α,β-dehydrocurvularin			Abiraterone acetate				
Data set	provided by M. T. B. Clabbers *et al.*			recorded for this study				
Raw data DOI	10.5281/zenodo.1407682			10.5281/zenodo.5579792				
Composition	$C_{16}H_{18}O_5$			$C_{26}H_{33}NO_2$				
Space group	$P2_12_12_1$			$P2_12_12_1$				
Absolute structure reference	CCDC code: 1300850			CCDC code: 2035079				
Author label	n14_a004	n14_a006	n14_a009	200811-01	200810-02	200811-03	200720-16	200720-19
Data set #	1	2	3	1	2	3	4	5
d_{min} (Å)	1.11	0.8	0.8	1.0	0.91	0.91	1.0	0.91
$\sin(\theta_{max})/\lambda$ (Å^{-1})	0.45	0.625	0.625	0.5	0.55	0.55	0.5	0.55
Completeness	59.4%	57.9%	54.7%	46.6%	52.6%	63.3%	71.6%	39.3%
N_{obs}	536	1564	1234	742	1185	1673	686	881
N_{all}	1240	1982	1580	1793	6128	5535	2677	2581
$N_{parameters}$	114	117	113	122	207	197	159	163
R_{obs} (enant. #1)	0.109	0.162	0.100	0.117	0.130	0.104	0.130	0.121
R_{all} (enant. #1)	0.189	0.177	0.113	0.236	0.415	0.262	0.338	0.285
wR_{all} (enant. #1)	0.118	0.183	0.105	0.133	0.144	0.112	0.139	0.123
R_{obs} (enant. #2)	0.115	0.178	0.145	0.143	0.156	0.131	0.167	0.150
R_{all} (enant. #2)	0.207	0.196	0.161	0.269	0.461	0.303	0.401	0.326
wR_{all} (enant. #2)	0.127	0.193	0.162	0.151	0.166	0.140	0.168	0.157
z-score	2.4σ	3.9σ	9.8σ	4.7σ	8.7σ	12.1σ	8.8σ	6.5σ
p	99.3%	100.0%	100.0%	100.0%	100.0%	100.0%	100.0%	100.0%

Tab. S21. Determination of the absolute structure of teniposide.
Refinement was performed against a combination of six data sets. Refinement statistics are reported for individual data sets and the combination of the 6 data sets.

Sample	Teniposide
Data set	provided by J. Bruhn *et al.*
Raw data DOI	10.5281/zenodo.3937740
Composition	$C_{32}H_{32}O_{13}S$
Space group	$P2_12_12_1$
Absolute structure reference	CCDC code: 2015361<br>Drugbank Accession Number: DB00444

Author label	951357	951398	951422	951544	954257	954272	
Data set #	1	2	3	4	5	6	combined
d_{min} (Å)	1.09	1.11	1.11	1.09	1.43	1.11	1.09
$\sin(\theta_{max})/\lambda$ (Å^{-1})	0.46	0.45	0.45	0.46	0.35	0.45	0.46
Completeness	71.3%	96.9%	95.8%	85.4%	43.1%	69.8%	99.8%
N_{obs}	645	2823	2234	2666	686	2127	11181
N_{all}	1726	6639	6888	7071	1652	5883	29859
$N_{parameters}$							733
R_{obs} (enant. #1)	0.206	0.200	0.208	0.187	0.217	0.199	0.199
R_{all} (enant. #1)	0.485	0.525	0.621	0.572	0.521	0.536	0.554
wR_{all} (enant. #1)	0.208	0.200	0.212	0.197	0.235	0.204	0.204
R_{obs} (enant. #2)	0.229	0.215	0.221	0.193	0.226	0.205	0.210
R_{all} (enant. #2)	0.517	0.530	0.641	0.576	0.531	0.545	0.565
wR_{all} (enant. #2)	0.228	0.211	0.228	0.202	0.246	0.213	0.215
z-score	3.1σ	2.3σ	1.4σ	0.7σ	1.5σ	1.4σ	3.8σ
p	99.9%	98.8%	91.8%	75.9%	93.7%	92.6%	100.0%

Tab. S22. Determination of the absolute structure of an amyloid peptide fragment.
Refinement was performed against a combination of six data sets. Refinement statistics are reported for individual data sets and the combination of the 6 data sets. As this structure was not reported before, a model with the determined absolute structure was deposited at the Cambridge Crystallographic Data Centre under deposition number 2238735.

Sample	Amyloid peptide fragment
Data set	provided by C. Bortolini *et al.*
Raw data DOI	10.5281/zenodo.5303223
Composition	$C_{37}H_{51}N_6O_7$
Space group	*C*2
Absolute structure reference	Absolute configuration of amino acids
CCDC code	2238735

Author label	2_11	2_13	3_20	4_26	5	6	
Data set #	1	2	3	4	5	6	combined
d_{min} (Å)	1.0	1.0	1.0	1.0	1.0	1.0	1.0
$\sin(\theta_{max})/\lambda$ (Å^{-1})	0.5	0.5	0.5	0.5	0.5	0.5	0.5
Completeness	28.3%	29.1%	15.1%	31.3%	52.6%	45.5%	87.2%
N_{obs}	824	889	522	361	1125	1007	4728
N_{all}	1931	2002	1255	1390	2768	2213	11559
$N_{parameters}$							385
R_{obs} (enant. #1)	0.171	0.163	0.167	0.163	0.168	0.173	0.168
R_{all} (enant. #1)	0.239	0.221	0.235	0.280	0.247	0.250	0.243
wR_{all} (enant. #1)	0.191	0.174	0.175	0.176	0.190	0.186	0.184
R_{obs} (enant. #2)	0.193	0.179	0.187	0.181	0.184	0.195	0.186
R_{all} (enant. #2)	0.266	0.241	0.253	0.300	0.264	0.270	0.263
wR_{all} (enant. #2)	0.210	0.192	0.193	0.194	0.208	0.212	0.203
z-score	6.4σ	4.2σ	2.0σ	2.1σ	4.5σ	3.8σ	9.7σ
p	100.0%	100.0%	97.5%	98.3%	100.0%	100.0%	100.0%

Tab. S23. **Determination of the absolute structure of FPTA (triclinic compound with *Z'* = 2).** Refinement was performed against a combination of 9 data sets. Constrained displacement parameters and distance restraints were applied for the two molecules in the asymmetric unit. Refinement statistics are reported for individual data sets and the combination of the 9 data sets.

Sample	FPTA
IUPAC name	(R)-N-(5-((3-((5-fluoropyrimidin-2-yl)methyl)piperidin-1-yl)methyl)thiazol-2-yl)acetamide
Data set	provided by J. Bruhn *et al.*
Raw data DOI	10.5281/zenodo.5799656
Composition	$C_{16}H_{20}FN_5OS$
Space group	*P*1
Absolute structure reference	CCDC code: 2130868

Author label	*36_3	*37_2	*38_3	*38_4	*38_5	*39_2	*54_3	*56_2	*57_3	
Data set #	1	2	3	4	5	6	7	8	9	comb.
d_{min} (Å)	0.83	0.83	0.83	1.25	0.83	0.83	0.83	1.0	1.0	0.83
$\sin(\theta_{max})/\lambda$ (Å^{-1})	0.6	0.6	0.6	0.4	0.6	0.6	0.6	0.5	0.5	0.6
Completeness	53.1%	54.2%	55.0%	49.0%	53.8%	55.4%	53.8%	62.5%	58.9%	94.7%
N_{obs}	1513	2186	2065	766	1998	2066	2240	1025	1551	15410
N_{all}	2605	3745	3815	1118	3782	3915	3741	2626	2463	27810
$N_{parameters}$										691
R_{obs} (enant. #1)	0.190	0.185	0.156	0.182	0.206	0.186	0.147	0.226	0.189	0.180
R_{all} (enant. #1)	0.283	0.274	0.253	0.254	0.323	0.291	0.247	0.421	0.259	0.282
wR_{all} (enant. #1)	0.197	0.176	0.160	0.183	0.212	0.176	0.145	0.207	0.188	0.182
R_{obs} (enant. #2)	0.202	0.198	0.171	0.185	0.214	0.197	0.162	0.231	0.197	0.191
R_{all} (enant. #2)	0.295	0.289	0.271	0.255	0.332	0.300	0.265	0.428	0.270	0.295
wR_{all} (enant. #2)	0.211	0.191	0.178	0.186	0.219	0.181	0.165	0.212	0.195	0.193
z-score	3.4σ	3.1σ	6.0σ	−0.2σ	3.3σ	3.7σ	5.0σ	2.2σ	2.0σ	13.5σ
p	100%	99.9	100%	41.4%	100%	100%	100%	98.7%	97.9%	100%

Tab. S24. Robustness of the determination of the absolute structure of (+)-limaspermidine. Refinement was performed against two data sets of the same crystal for the cases with $N_{OVF} > 59$, in the other cases only the first data set was used. N_{OVF} is the number of overlapping virtual frames used in the refinements, always starting with the first frame of the first data set.

Sample	(+)-limaspermidine
Data set	provided by Nelson Hosea
Composition	$C_{19}H_{26}N_2O$
Space group	$P2_12_12_1$
Absolute structure reference	CCDC code: 1049103

N_{OVF}	4	8	12	16	30	42	75	89	119
$\Delta\alpha_{max} - \Delta\alpha_{min}$	5.5°	9.2°	12.9°	16.6°	29.4°	40.5°	70.8°	83.7°	111.3°
d_{min} (Å)	0.83	0.83	0.83	0.83	0.83	0.83	0.83	0.83	0.83
$\sin(\theta_{max})/\lambda$ (Å^{-1})	0.6	0.6	0.6	0.6	0.6	0.6	0.6	0.6	0.6
Completeness	8.6%	13.2%	18.8%	24.9%	41.0%	50.3%	73.4%	85.3%	98.8%
N_{obs}	219	460	726	1017	2053	2981	5276	6179	7850
N_{all}	384	786	1190	1600	3028	4252	7571	8979	12016
$N_{parameters}$	77	81	86	90	104	116	150	164	194
R_{obs} (enant. #1)	0.0867	0.1122	0.121	0.124	0.135	0.136	0.138	0.138	0.145
R_{all} (enant. #1)	0.1901	0.2166	0.201	0.192	0.189	0.187	0.191	0.196	0.212
wR_{all} (enant. #1)	0.0909	0.114	0.123	0.125	0.133	0.137	0.140	0.140	0.148
R_{obs} (enant. #2)	0.1237	0.1327	0.154	0.162	0.171	0.172	0.176	0.176	0.182
R_{all} (enant. #2)	0.2479	0.2667	0.251	0.250	0.230	0.225	0.231	0.234	0.249
wR_{all} (enant. #2)	0.1315	0.1433	0.162	0.176	0.178	0.181	0.188	0.187	0.194
z-score	1.7σ	6.1σ	6.7σ	9.7σ	8.4σ	10.8σ	13.8σ	14.7σ	15.1σ
p	88.0%	100.0%	100.0%	100.0%	100.0%	100.0%	100.0%	100.0%	100.0%

References cited in the main text, methods and supplementary materials

1. Sheldrick, G. M. A short history of SHELX. *Acta Cryst A* **64**, 112–122 (2008).
2. Mugnaioli, E., Gorelik, T. & Kolb, U. “Ab initio” structure solution from electron diffraction data obtained by a combination of automated diffraction tomography and precession technique. *Ultramicroscopy* **109**, 758–765 (2009).
3. Palatinus, L. *et al.* Specifics of the data processing of precession electron diffraction tomography data and their implementation in the program PETS2.0. *Acta Crystallographica Section B-Structural Science Crystal Engineering and Materials* **75**, 512–522 (2019).
4. Gruene, T., Holstein, J. J., Clever, G. H. & Keppler, B. Establishing electron diffraction in chemical crystallography. *Nat Rev Chem* 1–9 (2021) doi:10.1038/s41570-021-00302-4.
5. Gemmi, M. *et al.* 3D Electron Diffraction: The Nanocrystallography Revolution. *ACS Central Science* **5**, 1315–1329 (2019).
6. Prince, E. *International Tables for Crystallography Volume C: Mathematical, physical and chemical tables*. (International Union of Crystallography, 2006). doi:10.1107/97809553602060000103.
7. Bethe, H. Theorie der Beugung von Elektronen an Kristallen. *Annalen der Physik* **392**, 55–129 (1928).
8. Zuo, J. M. & Spence, J. C. H. *Electron Microdiffraction*. (Springer US, 1992). doi:10.1007/978-1-4899-2353-0.
9. Own, C. S., Marks, L. D. & Sinkler, W. Precession electron diffraction 1: multislice simulation. *Acta Cryst A* **62**, 434–443 (2006).
10. Oleynikov, P., Hovmöller, S. & Zou, X. D. Precession electron diffraction: Observed and calculated intensities. *Ultramicroscopy* **107**, 523–533 (2007).
11. Spence, J. C. H., Zuo, J. M., O’Keeffe, M., Marthinsen, K. & Hoier, R. On the minimum number of beams needed to distinguish enantiomorphs in X-ray and electron diffraction. *Acta Cryst A* **50**, 647–650 (1994).

12. Inui, H., Fujii, A., Tanaka, K., Sakamoto, H. & Ishizuka, K. New electron diffraction method to identify the chirality of enantiomorphic crystals. *Acta Cryst B* **59**, 802–810 (2003).
13. Ma, Y., Oleynikov, P. & Terasaki, O. Electron crystallography for determining the handedness of a chiral zeolite nanocrystal. *Nature Materials* **16**, 755–759 (2017).
14. Brazda, P., Palatinus, L. & Babor, M. Electron diffraction determines molecular absolute configuration in a pharmaceutical nanocrystal. *Science* **364**, 667–669 (2019).
15. Palatinus, L., Petříček, V. & Corrêa, C. A. Structure refinement using precession electron diffraction tomography and dynamical diffraction: theory and implementation. *Acta Crystallogr A Found Adv* **71**, 235–244 (2015).
16. Wang, B. *et al.* A Porous Cobalt Tetraphosphonate Metal-Organic Framework: Accurate Structure and Guest Molecule Location Determined by Continuous-Rotation Electron Diffraction. *CHEMISTRY-A EUROPEAN JOURNAL* **24**, 17429–17433 (2018).
17. Rojas, A., Arteaga, O., Kahr, B. & Camblor, M. A. Synthesis, Structure, and Optical Activity of HPM-1, a Pure Silica Chiral Zeolite. *J. Am. Chem. Soc.* **135**, 11975–11984 (2013).
18. Tang, L. *et al.* A zeolite family with chiral and achiral structures built from the same building layer. *Nature Mater* **7**, 381–385 (2008).
19. Frojdh, E. *et al.* Discrimination of Aluminum from Silicon by Electron Crystallography with the JUNGFRAU Detector. *CRYSTALS* **10**, (2020).
20. Cichocka, M. O., Angstrom, J., Wang, B., Zou, X. & Smeets, S. High-throughput continuous rotation electron diffraction data acquisition via software automation. *Journal of Applied Crystallography* **51**, 1652–1661 (2018).
21. Broadhurst, E. T. *et al.* Polymorph evolution during crystal growth studied by 3D electron diffraction. *IUCrJ* **7**, 5–9 (2020).
22. Jones, C. G. *et al.* The CryoEM Method MicroED as a Powerful Tool for Small Molecule Structure Determination. *ACS Central Science* **4**, 1587–1592 (2018).

23. Gruene, T. *et al.* Rapid Structure Determination of Microcrystalline Molecular Compounds Using Electron Diffraction. *Angewandte Chemie-International Edition* **57**, 16313–16317 (2018).

24. Nakane, T. *et al.* Single-particle cryo-EM at atomic resolution. *Nature* **587**, 152–156 (2020).

25. Gruza, B., Chodkiewicz, M. L., Krzeszczakowska, J. & Dominiak, P. M. Refinement of organic crystal structures with multipolar electron scattering factors. *ACTA CRYSTALLOGRAPHICA A-FOUNDATION AND ADVANCES* **76**, 92–109 (2020).

26. Allen, F. H. & Bruno, I. J. Bond lengths in organic and metal-organic compounds revisited: X—H bond lengths from neutron diffraction data. *Acta Crystallographica Section B* **66**, 380–386 (2010).

27. Wang, B. *et al.* Absolute configuration determination of pharmaceutical crystalline powders by MicroED via chiral salt formation. *Chemical Communications* **58**, 4711–4714 (2022).

28. Le Page, Y., Gabe, E. J. & Gainsford, G. J. A robust alternative to η refinement for assessing the hand of chiral compounds. *J Appl Cryst* **23**, 406–411 (1990).

29. Dong, Z. & Ma, Y. Atomic-level handedness determination of chiral crystals using aberration-corrected scanning transmission electron microscopy. *Nature Communications* **11**, 1588 (2020).

30. Gemmi, M., La Placa, M. G. I., Galanis, A. S., Rauch, E. F. & Nicolopoulos, S. Fast electron diffraction tomography. *Journal of Applied Crystallography* **48**, 718–727 (2015).

31. Parsons, S., Flack, H. D. & Wagner, T. Use of intensity quotients and differences in absolute structure refinement. *Acta Cryst B* **69**, 249–259 (2013).

32. Escudero-Adán, E. C., Benet-Buchholz, J. & Ballester, P. The use of Mo Kα radiation in the assignment of the absolute configuration of light-atom molecules; the importance of high-resolution data. *Acta Cryst B* **70**, 660–668 (2014).

33. Latychevskaia, T. & Abrahams, J. P. Inelastic scattering and solvent scattering reduce dynamical diffraction in biological crystals. *Acta Crystallographica Section B-Structural Science Crystal Engineering and Materials* **75**, 523–531 (2019).

34. Pastero, L., Turci, F., Leinardi, R., Pavan, C. & Monopoli, M. Synthesis of α-Quartz with Controlled Properties for the Investigation of the Molecular Determinants in Silica Toxicology. *Crystal Growth & Design* **16**, 2394–2403 (2016).

35. Palatinus, L. *et al.* Hydrogen positions in single nanocrystals revealed by electron diffraction. *Science* **355**, 166–169 (2017).

36. Zaarour, M. *et al.* Synthesis of new cobalt aluminophosphate framework by opening a cobalt methylphosphonate layered material. *CrystEngComm* **19**, 5100–5105 (2017).

37. Zhou, H. *et al.* Programming Conventional Electron Microscopes for Solving Ultrahigh-Resolution Structures of Small and Macro-Molecules. *Analytical Chemistry* **91**, 10996–11003 (2019).

38. Plana-Ruiz, S. *et al.* Fast-ADT: A fast and automated electron diffraction tomography setup for structure determination and refinement. *Ultramicroscopy* **211**, (2020).

39. Roslova, M. *et al.* InsteaDMatic: towards cross-platform automated continuous rotation electron diffraction. *Journal of Applied Crystallography* **53**, 1217–1224 (2020).

40. Bortolini, C. *et al.* Atomic Structure of Amyloid Crystals. (2022) doi:10.5281/zenodo.5303223.

41. Yanagisawa, H., Yamashita, K., Nureki, O. & Kikkawa, M. MicroED datasets of hemin and biotin collected on Ceta camera. (2019) doi:10.5281/zenodo.3366892.

42. Thompson, E. & Jenkins, H. T. 3DED/microED datasets of biotin (Glacios/Ceta-D). (2021) doi:10.5281/zenodo.4895412.

43. Bruhn, J. F. *et al.* Small Molecule Microcrystal Electron Diffraction for the Pharmaceutical Industry–Lessons Learned From Examining Over Fifty Samples. *Frontiers in Molecular Biosciences* **8**, 354 (2021).

44. Clabbers, M. T. B., Gruene, T., van Genderen, E. & Abrahams, J. P. Reducing dynamical electron scattering reveals hydrogen atoms. *ACTA CRYSTALLOGRAPHICA A-FOUNDATION AND ADVANCES* **75**, 82–93 (2019).

45. Knudsen, E. B., Sørensen, H. O., Wright, J. P., Goret, G. & Kieffer, J. FabIO: easy access to two-dimensional X-ray detector images in Python. *J Appl Cryst* **46**, 537–539 (2013).

46. Palatinus, L. & Chapuis, G. SUPERFLIP – a computer program for the solution of crystal structures by charge flipping in arbitrary dimensions. *J Appl Cryst* **40**, 786–790 (2007).

47. Petříček, V., Dušek, M. & Palatinus, L. Crystallographic Computing System JANA2006: General features. *Zeitschrift für Kristallographie - Crystalline Materials* **229**, 345–352 (2014).

48. Palatinus, L. *et al.* Structure refinement using precession electron diffraction tomography and dynamical diffraction: tests on experimental data. *Acta Crystallogr B Struct Sci Cryst Eng Mater* **71**, 740–751 (2015).

49. Palatinus, L. *et al.* Structure refinement from precession electron diffraction data. *Acta Crystallographica Section A* **69**, 171–188 (2013).

50. Kolb, U., Gorelik, T. & Otten, M. T. Towards automated diffraction tomography. Part II - Cell parameter determination. *Ultramicroscopy* **108**, 763–772 (2008).

51. Zhang, D., Oleynikov, P., Hovmoller, S. & Zou, X. Collecting 3D electron diffraction data by the rotation method. *Zeitschrift Fur Kristallographie-Crystalline Materials* **225**, 94–102 (2010).

52. Nannenga, B. L., Shi, D., Leslie, A. G. W. & Gonen, T. High-resolution structure determination by continuous-rotation data collection in MicroED. *Nature Methods* **11**, 927–930 (2014).

53. Wang, Y., Yang, T., Xu, H., Zou, X. & Wan, W. On the quality of the continuous rotation electron diffraction data for accurate atomic structure determination of inorganic compounds. *Journal of Applied Crystallography* **51**, 1094–1101 (2018).

54. Kolb, U., Mugnaioli, E. & Gorelik, T. E. Automated electron diffraction tomography - a new tool for nano crystal structure analysis. *Crystal research and technology* **46**, 542–554 (2011).

55. Shi, D., Nannenga, B. L., Iadanza, M. G. & Gonen, T. Three-dimensional electron crystallography of protein microcrystals. *ELIFE* **2**, (2013).

56. Yun, Y. *et al.* Phase identification and structure determination from multiphase crystalline powder samples by rotation electron diffraction. *Journal of applied crystallography* **47**, 2048–2054 (2014).

57. Wan, W., Sun, J., Su, J., Hovmoller, S. & Zou, X. Three-dimensional rotation electron diffraction: software RED for automated data collection and data processing. *Journal of applied crystallography* **46**, 1863–1873 (2013).

58. Kolb, U., Gorelik, T., Kübel, C., Otten, M. T. & Hubert, D. Towards automated diffraction tomography: Part I—Data acquisition. *Ultramicroscopy* **107**, 507–513 (2007).

59. Kabsch, W. XDS. *Acta Cryst D* **66**, 125–132 (2010).

60. Groom, C. R., Bruno, I. J., Lightfoot, M. P. & Ward, S. C. The Cambridge Structural Database. *Acta Cryst B* **72**, 171–179 (2016).

61. Steciuk, G. *et al.* Precession electron diffraction tomography on twinned crystals: application to CaTiO3 thin films. *JOURNAL OF APPLIED CRYSTALLOGRAPHY* **52**, 626–636 (2019).

62. Lignie, A., Granier, D., Armand, P., Haines, J. & Papet, P. Modulation of quartz-like GeO2 structure by Si substitution: an X-ray diffraction study of Ge1−xSixO2 ($0 \leq x < 0.2$) flux-grown single crystals. *J Appl Cryst* **45**, 272–278 (2012).

63. Aree, T. & Bürgi, H.-B. Dynamics and Thermodynamics of Crystalline Polymorphs: α-Glycine, Analysis of Variable-Temperature Atomic Displacement Parameters. *J. Phys. Chem. A* **116**, 8092–8099 (2012).

64. Will, G., Bellotto, M., Parrish, W. & Hart, M. Crystal structures of quartz and magnesium germanate by profile analysis of synchrotron-radiation high-resolution powder data. *Journal of Applied Crystallography* **21**, 182–191 (1988).

65. Downs, R. T., Hazen, R. M. & Finger, L. W. The high-pressure crystal chemistry of low albite and the origin of the pressure dependency of Al-Si ordering. *American Mineralogist* **79**, 1042–1052 (1994).

66. Capitelli, F. & Derebe, M. G. Single Crystal X-ray Diffraction Study of a Pure Natrolite Sample. *J Chem Crystallogr* **37**, 583–586 (2007).

67. Gale, J. D. GULP: Capabilities and prospects. *Zeitschrift für Kristallographie - Crystalline Materials* **220**, 552–554 (2005).

68. Jackson, R. A. & Catlow, C. R. A. Computer Simulation Studies of Zeolite Structure. *Molecular Simulation* **1**, 207–224 (1988).

69. Groom, C. R. & Allen, F. H. The Cambridge Structural Database in Retrospect and Prospect. *Angewandte Chemie International Edition* **53**, 662–671 (2014).

70. Sovago, I. *et al.* Electron density, disorder and polymorphism: high-resolution diffraction studies of the highly polymorphic neuralgic drug carbamazepine. *Acta Cryst B* **72**, 39–50 (2016).

71. Du, J.-Y. *et al.* Asymmetric Total Synthesis of Apocynaceae Hydrocarbazole Alkaloids (+)-Deethylibophyllidine and (+)-Limaspermidine. *J. Am. Chem. Soc.* **137**, 4267–4273 (2015).

72. Korlyukov, A. A., Vologzhanina, A. V., Trzybinski, D., Malinska, M. & Wozniak, K. Charge density analysis of abiraterone acetate. *Acta Cryst B* **76**, 1018–1026 (2020).
